# Supplementary material for: Lineage specification in glioblastoma is regulated by METTL7B
Source: Cell Rep. 2024 Jun 5;43(6):114309. doi: 10.1016/j.celrep.2024.114309 (PMC11220825; doi:10.1016/j.celrep.2024.114309)
Supplement: Document S1. Figures S1‒S10 [file mmc1.pdf]

Cell Reports, Volume *43*

Supplemental information

Lineage specification in glioblastoma is regulated by METTL7B

Myrianni Constantinou, James Nicholson, Xinyu Zhang, Eleni Maniati, Sara Lucchini, Gabriel Rosser, Claire Vinel, Jun Wang, Yau Mun Lim, Sebastian Brandner, Sven Nelander, Sara Badodi, and Silvia Marino

Constantinou et al. Supplementary Figures and Figure Legends

Constantinou et al. Figure S1

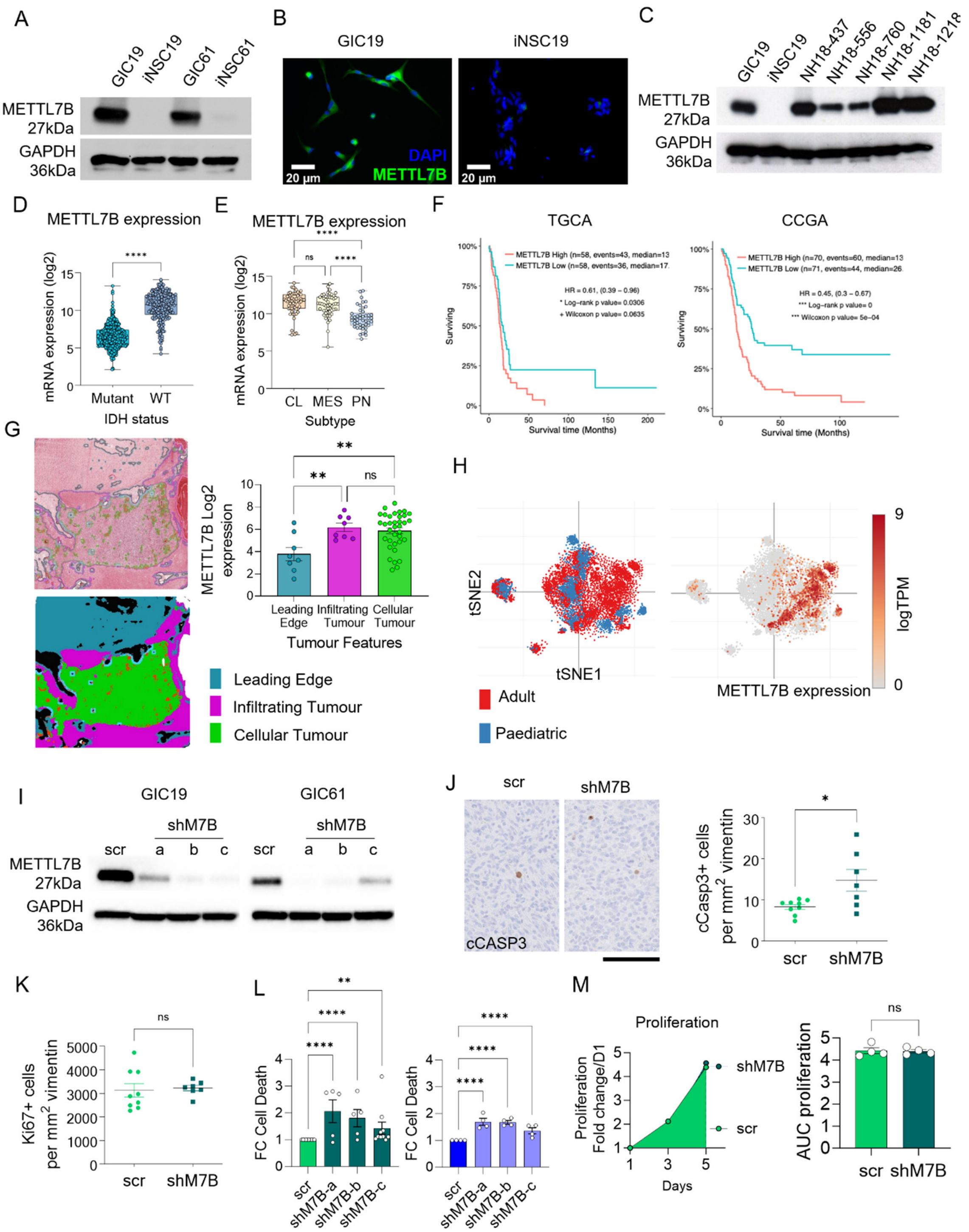

Figure S1 METTL7B is overexpressed in IDH-wildtype glioblastoma. Related to Figure 1

A. Western blot of METTL7B in syngeneic pairs GIC19/iNSC19, GIC61/iNSC61. GAPDH used as a loading control.

B. Immunofluorescence staining of METTL7B (green) in iNSC19 and GIC19 cells. Nuclei were counterstained with DAPI. Scale bar=20µm.

C. Western blot of METTL7B in 5 primary GIC lines from an independent cohort compared to iNSC19 and GIC19. GAPDH used as a loading control.

D. Analysis of *METTL7B* expression in IDH- mutant and wild-type (WT) gliomas. N= 429 mutant and 233 WT IDH independent glioma samples from TCGA. Two-tailed unpaired t-test, ****p<0.0001. Whiskers showing min and max points.

E. Analysis of *METTL7B* expression in glioblastoma subtypes, n= 56 Classical, 51 Mesenchymal and 46 Proneural independent glioma samples from TCGA. 2-way ANOVA, p-value of the pairwise comparisons is shown. ****p-value<0.0001, ns, not significant. Whiskers showing min and max points.

F. Survival curves of glioma, IDH-wildtype patients from TCGA cohort (M7B high n=58, M7B low n=58) and CGGA cohort (M7B high n=70, M7B low n=71).

G. Spatial expression of METTL7B in GBM bulk samples, analysed on Ivy (GAP). Left: example of histological anatomic structure identification in a sub-block. Right: Expression of *METTL7B* in RNAseq data from anatomic structures shown as log2 normalized gene expression. Leading Edge defined as the border of the tumour, where ratio of tumour to normal cells is 1-3 / 100. Infiltrating tumour defined as the intermediate zone between leading edge and cellular tumour, where ratio of tumour to normal cells is 10-20 /100. Cellular tumour defined as tumour core, where tumour to normal cells is 100-500 / 1. Graphs show mean ± SEM. 2-way ANOVA, p-value of the pairwise comparisons is shown. **p-value<0.001.

H. *METTL7B* expression in scRNAseq data of paediatric (blue) and adult (red) glioblastoma samples. (Clusters defined in Neftel et al, Cell 2019 [1], data accessed and visualized on Single Cell Portal of The Broad Institute of MIT and Harvard. METTL7B expression in tSNE, with logTPM expression ranging from light orange to dark.

I. Western blot analysis of protein expression levels of METTL7B upon silencing in GIC19 and GIC61 (shM7B) using three different shRNA (-a, -b, -c), as compared to the scramble control (scr).

J. IHC staining for cleaved-Caspase 3 (cCasp3) in brains of mice orthotopically injected with GICscr and shM7B and quantification of cCasp3-positive cells per mm^2 of tumour. Light green: scr n=9 animals, Dark green: shM7B n=7 animals. Welch's t test, *p-value=0.049. Scale bar = 100 μm.

K. Proliferation assessment of GIC in PDX model as quantitative assessment of Ki67+ cells in the xenografts, Welch's t test, p-value=0.77. Light green: scr n=9 animals. Dark green: shM7B n=7 animals

L. Cell death in GIC19 and GIC61 upon METTL7B silencing (GIC19 shM7B, dark green, GIC61 shM7B, light blue) as compared to control (GIC19 scr, light green, GIC61 scr, dark blue).For GIC19 (scr: N=10, n=3-5, shM7B-a: N=5, n=3-5, shM7B-b: N=5, n=3-5, shM7B-c: N=10, n=3-5). For GIC61 (N=4 for all groups, and n=4-10 technical replicas). Two-way ANOVA, error bars as SEM, p-values: **p-value<0.01, ***p-value<0.001, ****p-value<0.0001.

M. Proliferation rate of GIC19 upon METTL7B silencing (shM7B-c, dark green) as compared to control (scr, light green). Proliferation difference is shown as area under the curve over 5 days. (N=4 biological replicates., Two-tailed unpaired t-test, error bars as SEM, p-values: ***p-value<0.001, ns not significant.

Constantinou et al. Figure S2

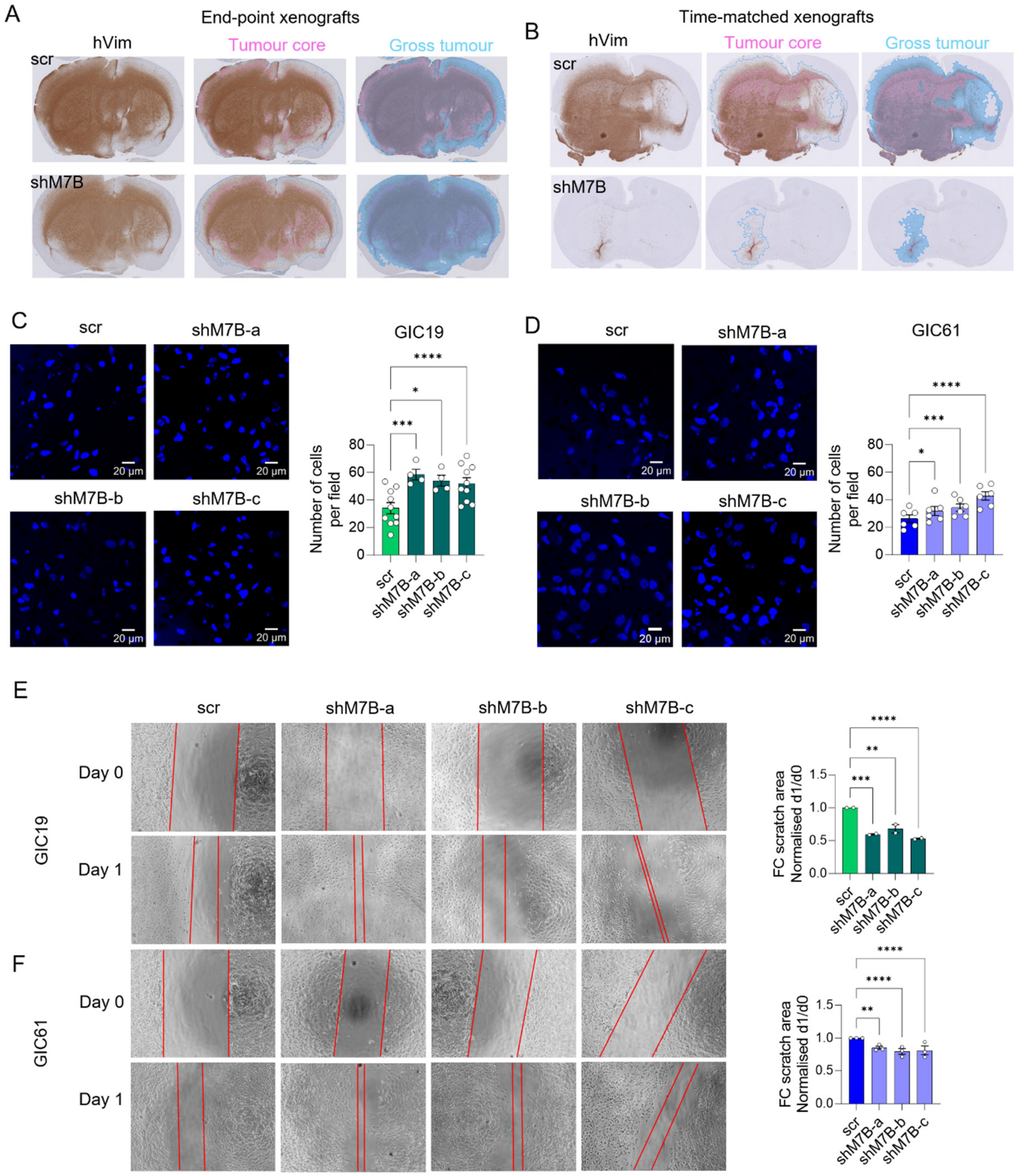

Figure S2 METTL7B silencing in GIC leads to increased invasion. Related to Figure 1

A. Vimentin stained sections of xenografts at end-point overlayed with the masks used to quantify the invasiveness index. The invasiveness index is defined as gross tumour area (blue) /tumour core area (pink).

B. Vimentin stained sections of time-matched xenografts overlayed with the masks used to quantify the invasiveness index. The invasiveness index is defined as gross tumour area (blue) /tumour core area (pink).

C. Transwell assay to assess invasion of GIC19 upon METTL7B silencing with three different shRNA (GIC19 shM7B, dark green) as compared to control (GIC19 scr, light green). Representative images of scr and shM7B captured at 40x magnification (scale bar at 20 µm). Graph shows the number of cells that migrated through the transwelll membrane per image field captured. N=3 independent experiments, 2-3 membranes per replica and 5-7 images per membrane captured at 40x. Two-Way ANOVA, error bars as SEM, p-values: *p-value<0.05, **p-value<0.01, ***p-value<0.001, ****p-value<0.0001.

D. Transwell assay to assess invasion of GIC61 upon METTL7B silencing with three different shRNA (GIC61 shM7B, light blue) as compared to control (GIC61 scr, dark blue). Representative images of scr and shM7B captured at 40x (scale bar at 20 µm). Graph shows the number of cells that migrated through the transwelll membrane per image field captured. N=3 independent experiments, 2-3 membranes per replica and 5-7 images per membrane captured at 40x magnification. Two-way ANOVA, error bars as SEM, p-values: *p-value<0.05, **p-value<0.01, ***p-value<0.001, ****p-value<0.0001.

E. Scratch-wound healing assay to assess migration of GIC19 upon METTL7B silencing with three different shRNA (GIC19 shM7B, dark green) as compared to control (GIC19 scr, light green). Representative images brightfield images of scr and shM7B captured at 10x. Red lines indicate the scratch area on day 0 and day 1. Graph shows fold change of scr and shM7B of the scratch area on day 1 normalised to day 0. N=2 independent experiments, 2-5 technical replicas (wells) per condition. Two-way ANOVA p-values, error bars as SEM, p-values: *p-value<0.05, **p-value<0.01, ***p-value<0.001, ****p-value<0.0001.

F. Scratch-wound healing assay to assess migration of GIC61 upon METTL7B silencing with three different shRNA (GIC61 shM7B, light blue) as compared to control (GIC61 scr, dark blue). Representative images brightfield images of scr and shM7B captured at 10x. Red lines indicate the scratch area on day 0 and day 1. Graph reports fold

change of scr and shM7B of the scratch area on day 1 normalised to day 0. N=3 independent experiments, 2-5 technical replicas (wells) per condition. Two-way ANOVA, error bars as SEM, p-values: *p-value<0.05, **p-value<0.01, ***p-value<0.001, ****p-value<0.0001.

Constantinou et al. Figure S3

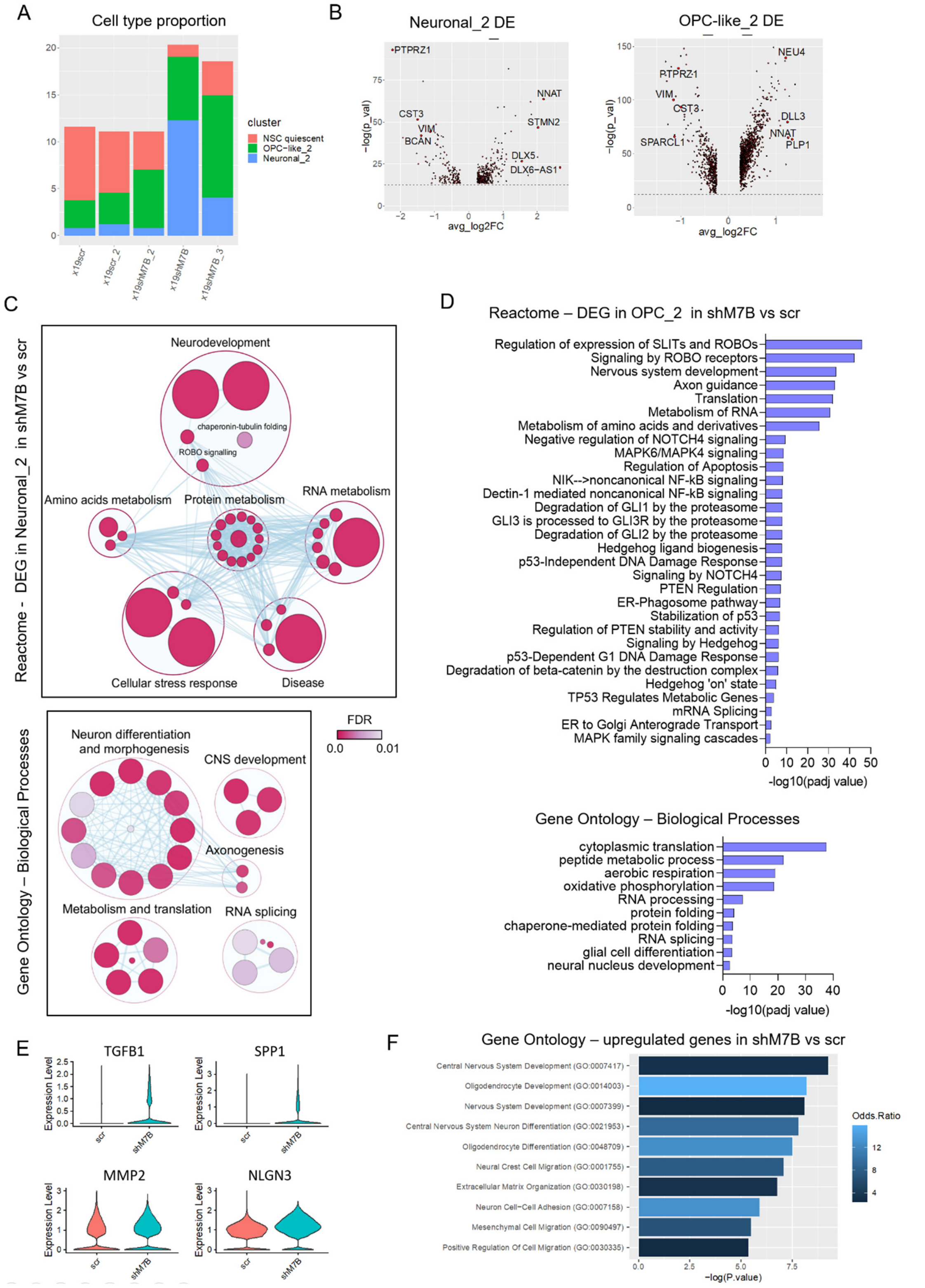

Figure S3 Single-cell RNAseq analysis and DE genes in GICshM7B derived xenografts and controls. Related to Figure 2

A. Cell type proportions of most affected clusters in scr and shM7B Cell cycle classification of NSC/NPC clusters.

B. Volcano plots showing DE genes in GICshM7B as compared to scr in Neuronal 2 (top) and OPC-like 2 (bottom) clusters. X axis: average log2 fold change (avg_log2FC), Y axis: -log(p_val). Upregulated genes in GICshM7B: avg_log2FC>0, Downregulated genes in GICshM7B: avg_log2FC<0.

C. Gene set enrichment analysis of Neuronal_2 DE genes in GICshM7B as compared to control scr, of significantly affected Reactome pathways (left) and gene ontology biological processes (GOBP, right) visualised on Cytoscape. Bubbles are coloured based on FDR values and size is proportional to the number of genes included in each Reactome or GO term.

D. Gene set enrichment analysis of OPC-like_2 DE genes in GICshM7B as compared to control scr, of the 30 most significantly affected Reactome pathways (top) and top 10 GOBP (bottom) visualised on Cytoscape. Bars showing –log10(padj).

E. Violin plots show expression levels of invasion/migration markers in the two groups (scr and shM7B).

F. GO analysis of upregulated genes in shM7B as compared to scr.

Constantinou et al. Figure S4

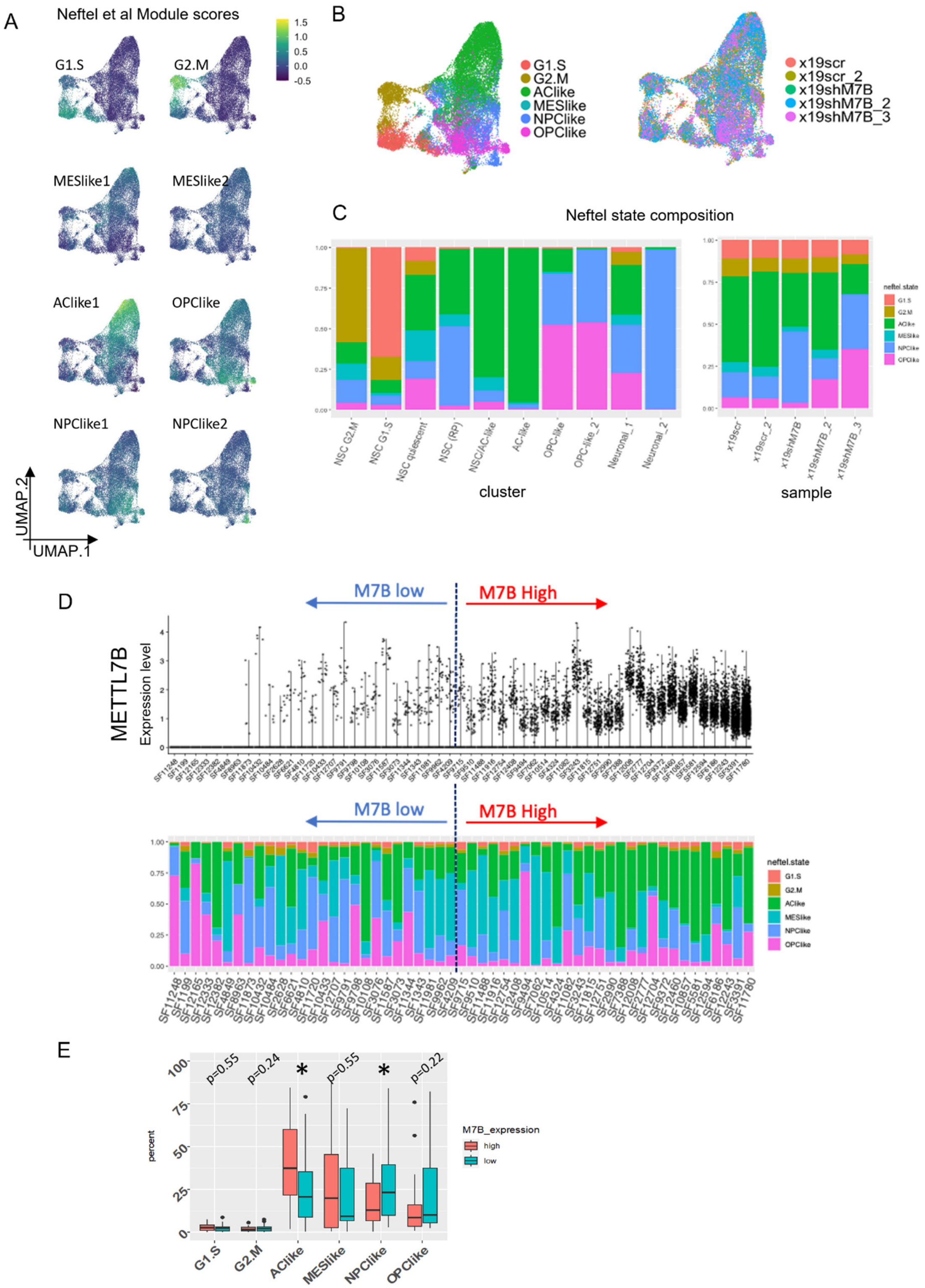

Figure S4 High METTL7B expression is associated with the AC-like state in scRNAseq datasets. Related to Figure 2

A. UMAPs showing single cell expression enrichment of patient-derived GIC cell state signature genes.

B. UMAP showing Neftel cell state identity (assigned by highest signature score) and plot of cell state proportions in each sample.

C. Bar plots showing composition of signature scoring assigned Neftel cell state identities in cell types (left) and samples (right) .

D. Violin plot showing High (n=27) and low (n=27) METTL7B expression groups in a cohort of patient GBM scRNAseq samples (Wang et al., 2022) (top panel), as compared to their signature scoring assigned Neftel cell state compositions (bottom panel)

E. Bar graphs showing the cell state composition in samples with high and low METTL7B expression.

Constantinou et al. Figure S5

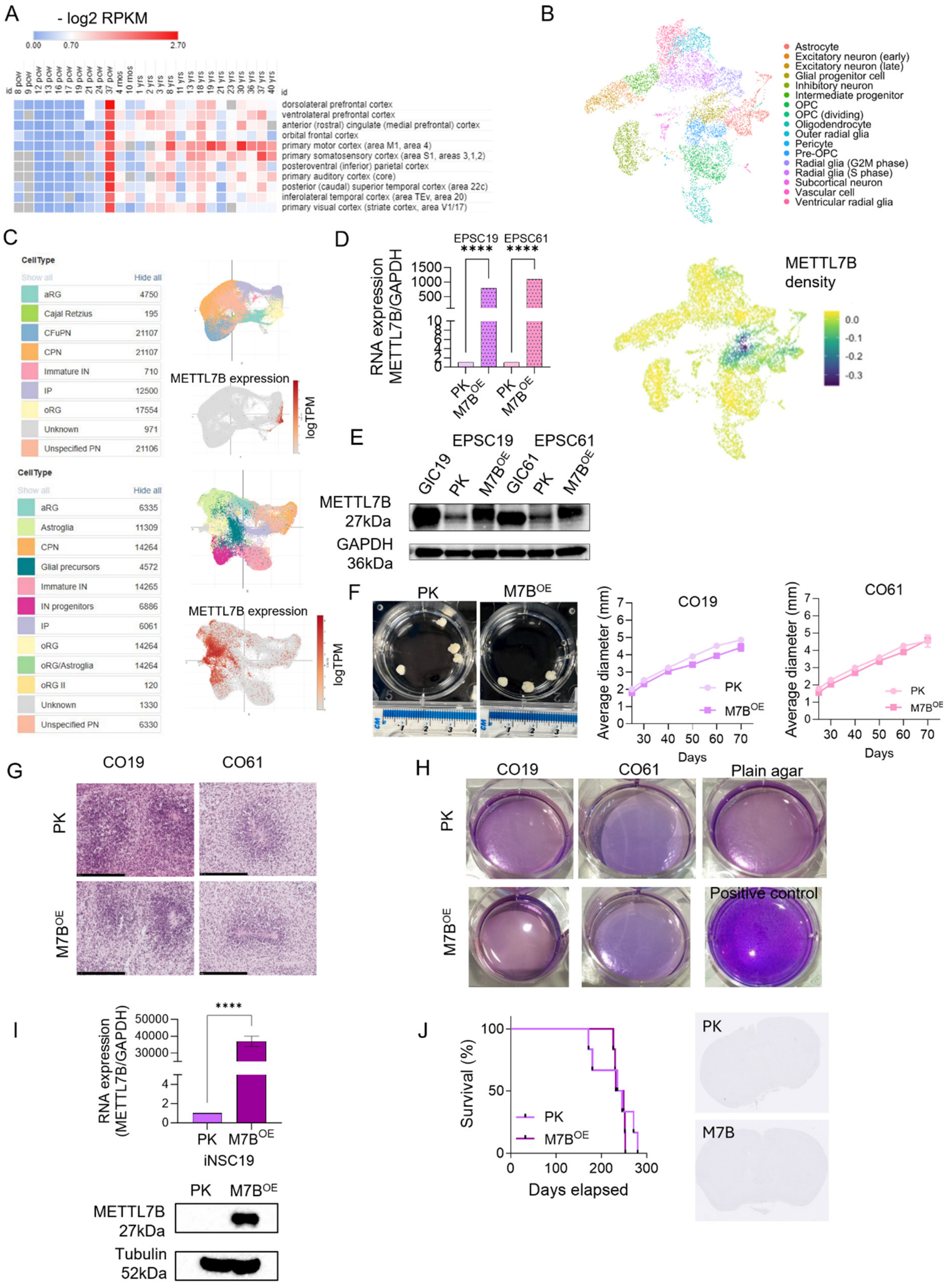

Figure S5 METTL7B is expressed during neurodevelopment. Related to Figure 3

A. Expression of METTL7B in the developing and adult human cortex, from 8pcw to 40 years, extracted from the BrainSpan Atlas. RNA seq data analysed for different developing regions of the developing cortex. Expression is shown as log2 RPKM (reads per kilobase per million) values of n=3 for each time point.

B. Single-cell RNAseq on 17-19GW fetal brain samples. Left shows UMAP of cells colored by cell type, right shows expression density plot of METTL7B.

C. Single-cell RNAseq on 3-month (top) and 6-month (bottom) COs. N=3-4 COs per timepoint. Left: Clusters annotations of UMAP (right top). Right bottom: METTL7B expression counts in the clusters, shown in a 2D plot as logTPM expression ranging from light orange to dark.

D. METTL7B relative RNA expression levels in METTL7B overexpressing EPSC19 and EPSC61 as compared to control (PK) (n=3, biological replicas, n=3 technical replicas). Ordinary one-way ANOVA, ****P<0.0001.

E. METTL7B protein expression levels in METTL7B overexpressing EPSC19 and EPSC61 as compared to control (PK), and syngeneic GIC pairs.

F. Cerebral organoids at day 70 of CO19 PK and M7B. The average diameter in mm was measured from day 25 to 70 of CO19 and CO61 PK and M7B. Quantification of average diameter of 3-4 COs per condition for each line.

G. H&E of day 70 COs PK and M7B in both lines. Scale bar is 250µm.

H. Soft agar assay at 3 weeks post seeding of dissociated PK and M7B COs from both lines. Plain agar: negative control, NSC overexpressing NFIX: positive control. Crystal violet stain at end point.

I. Left: METTL7B RNA expression in iNSC19 upon overexpression of METTL7B ($M7B^{OE}$, dark purple), as compared to the control (PK, light purple). N=4: independent lentiviral transductions. Right: Protein expression levels of METTL7B in iNSC19 upon overexpression of METTL7B, as compared to the PK control. Tubulin was used as a loading control.

J. Survival curves of mice injected with control PK (light purple) and iNSC19 $M7B^{OE}$ (dark purple). (n=6 for each group). Left: hVim immunostaining of mouse brains injected with iNSC19 PK and $M7B^{OE}$

Constantinou et al. Figure S6

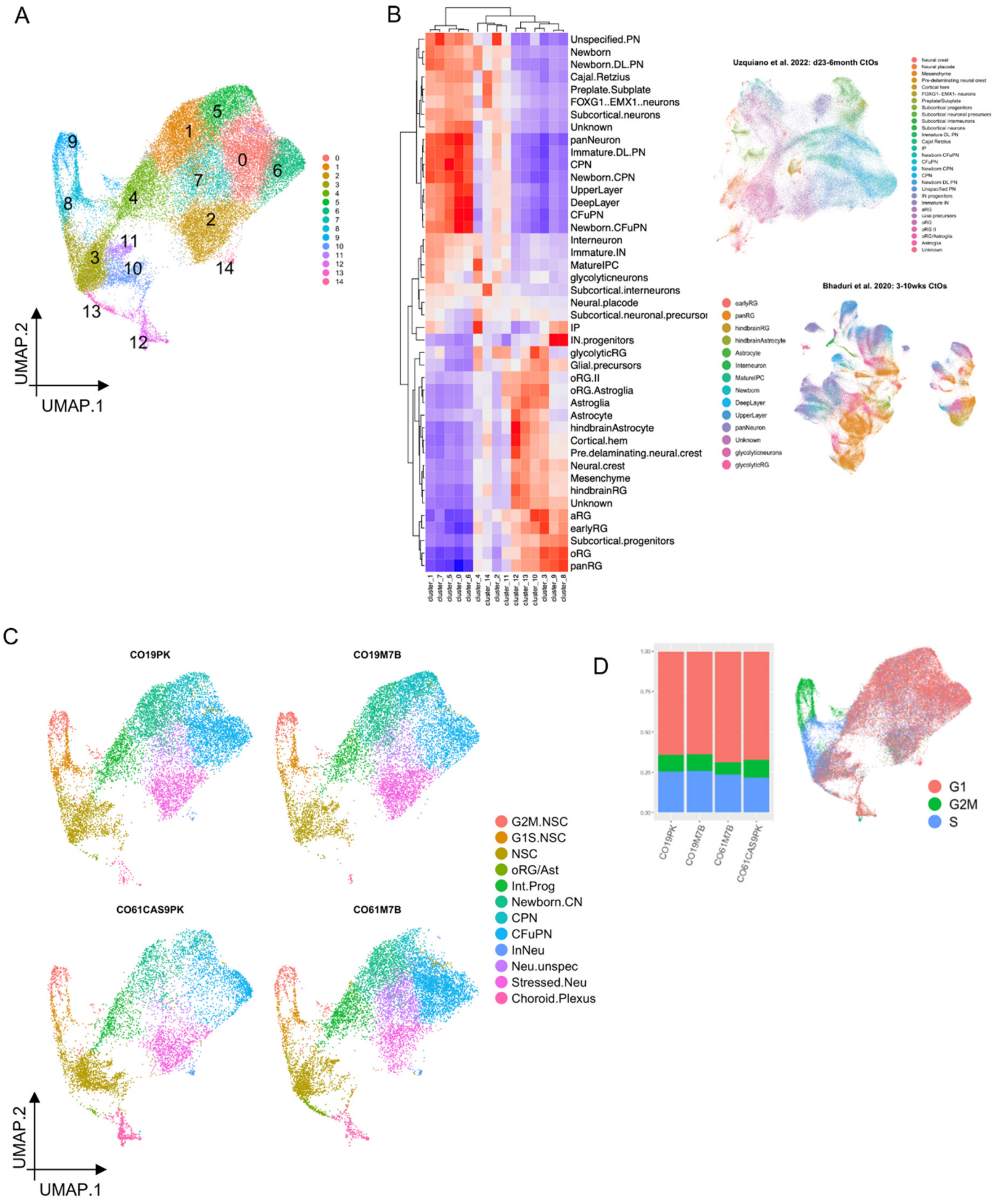

Figure S6 Single-cell RNAseq analysis and cell-type distribution in day70 CO derived from EPSC M7B^{OE}. Related to Figure 3

A. UMAP plot of CO samples coloured by Seurat clusters.
B. Heatmap showing Pearson correlation of cluster marker genes to reference cell type signatures.
C. UMAPs showing cell type annotations split by organoid sample.
D. UMAP and bar plot showing cell cycle state proportions assigned by module scoring.

Constantinou et al. Figure S7

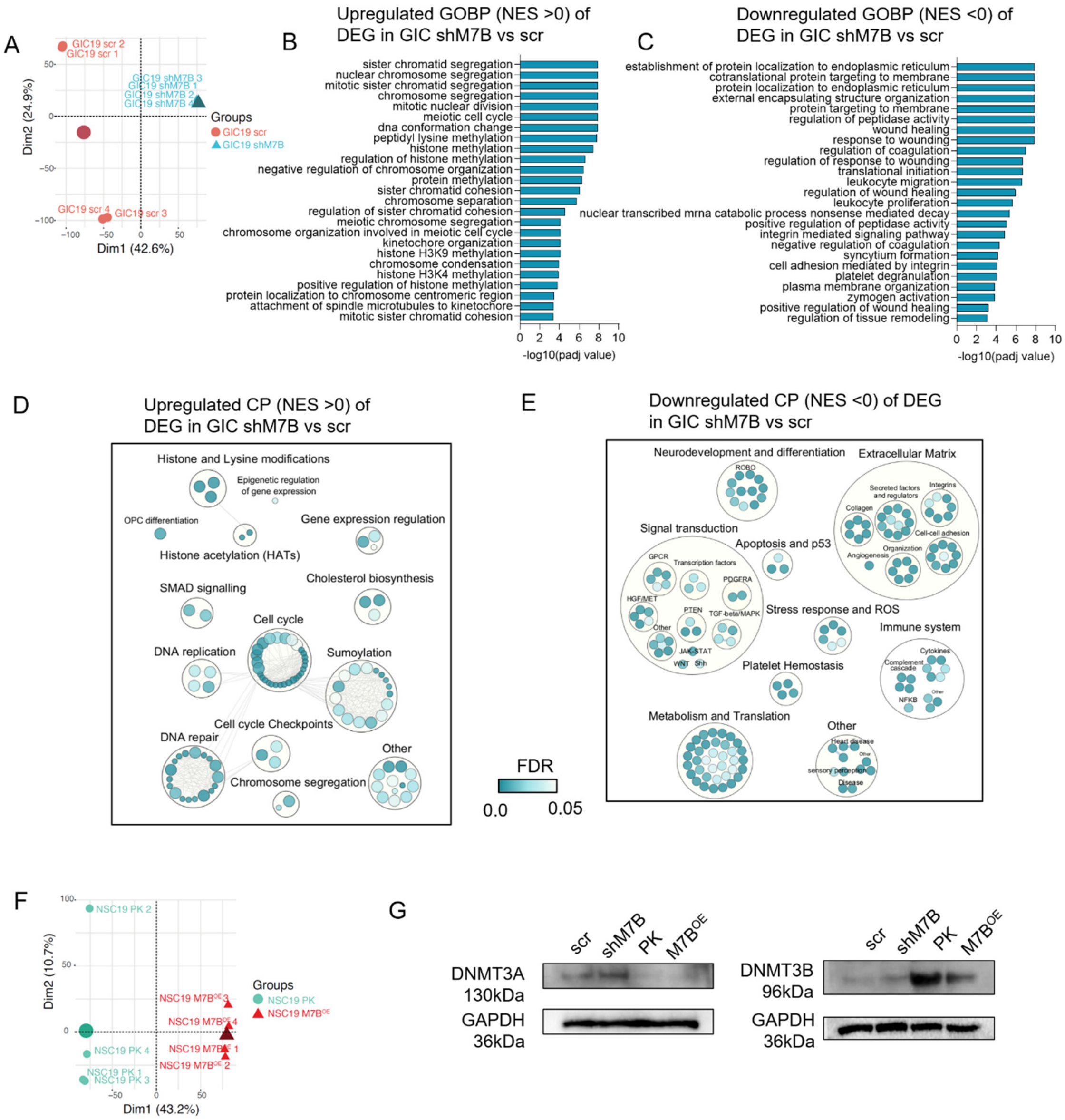

Figure S7 Bulk RNAseq analysis in GIC and NSC upon METTL7B modulation. Related to Figure 4

A. PCA plot of samples using gene expression of all detected genes, using gene expression log2 Reads per kilobase of transcript per Million reads (log2RPKM) of all sufficiently detected genes. Red circle: GIC19 scr replicas (n=4), dark red: centroid of GIC19 scr, Blue triangle: GIC19 shM7B replicas (n=4), dark blue: centroid of GIC shM7B.

B. Top 25 most significantly upregulated gene ontology biological processes (GOBP) (NES>0) of all DE genes identified in GIC19 shM7B as compared to scr. GSEA using differential expression statistic (t-statistic). Bars showing –log10(padj).

C. Top 25 most significantly downregulated gene ontology biological processes (GOBP) (NES<0) of all DE genes identified in GIC19 shM7B as compared to scr. GSEA using differential expression statistic (t-statistic). Bars showing –log10(padj).

D. Cytoscape visualisation of upregulated (NES>0) Canonical pathways (CP) of all DE genes identified in GIC19 shM7B as compared to scr. GSEA using differential expression statistic (t-statistic). Bubbles are coloured based on FDR values and size is proportional to the number of genes included in each CP term.

E. Cytoscape visualisation of downregulated (NES<0) Canonical pathways (CP) of all DE genes identified in GIC19 shM7B as compared to scr. GSEA using differential expression statistic (t-statistic). Bubbles are coloured based on FDR values and size is proportional to the number of genes included in each CP term.

F. PCA plot of samples using gene expression of all detected genes, using gene expression log2RPKM of all sufficiently detected genes. Green circle: iNSC19 PK replicas (n=4), dark green: centroid of iNSC19 PK, Red triangle: iNSC M7B^{OE} replicas (n=4), dark red: centroid of iNSC19 M7B^{OE}.

G. Protein expression levels of DNMT3A and DNMT3B upon silencing of METTL7B in GIC19, and overexpression of METTL7B in iNSC19. 20µg of protein lysate loaded for each sample. Representative images of n=3 replicas.

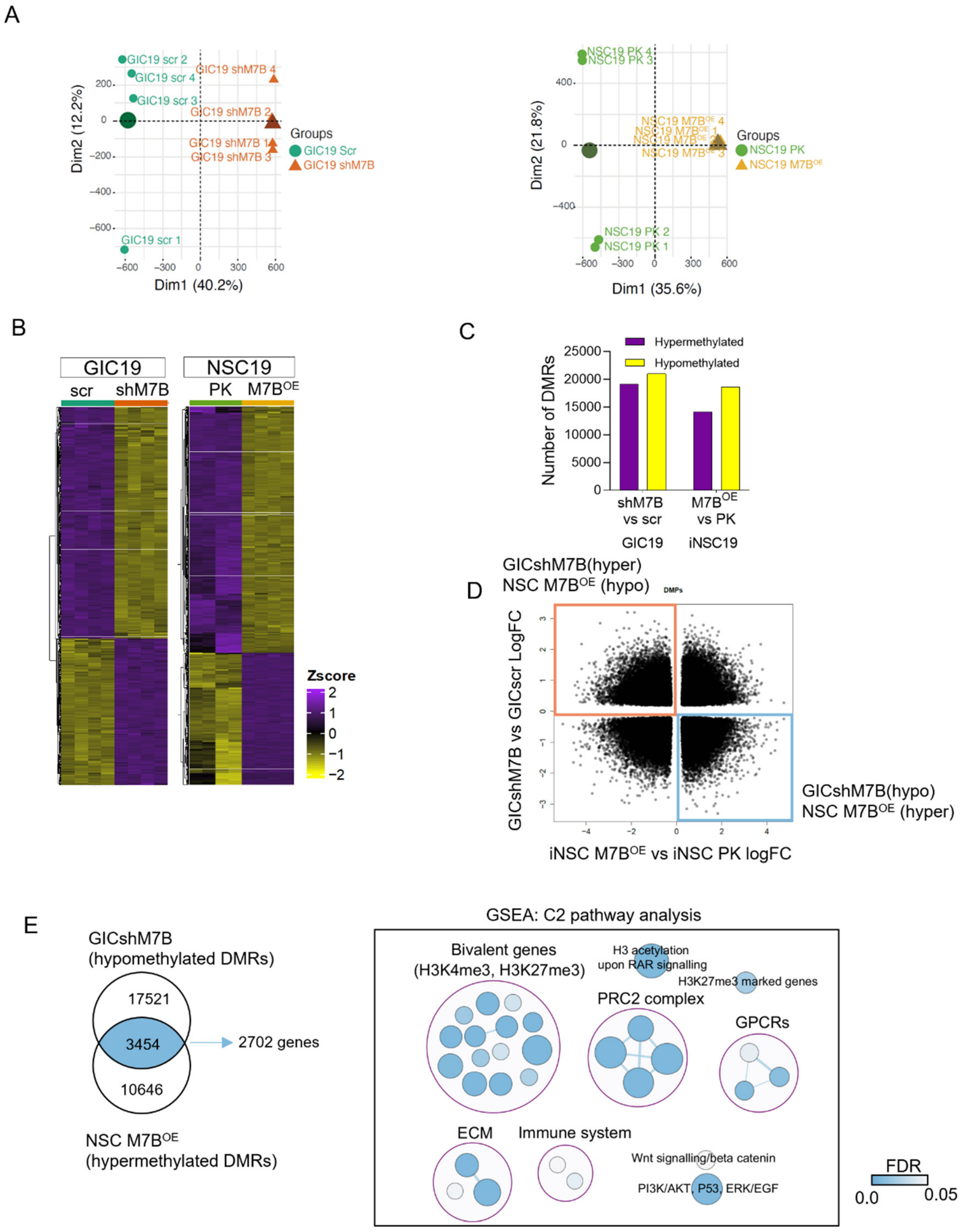
A
GIC19 scr 2
GIC19 scr 4
GIC19 scr 3
GIC19 scr 1
GIC19 shM7B 4
GIC19 shM7B 2
GIC19 shM7B 1
GIC19 shM7B 3
Groups
GIC19 Scr
GIC19 shM7B
Dim2 (12.2%)
Dim1 (40.2%)
NSC19 PK 4
NSC19 PK 3
NSC19 PK 2
NSC19 PK 1
Groups
NSC19 PK
NSC19 M7B^OE
Dim2 (21.8%)
Dim1 (35.6%)
B
GIC19
scr
shM7B
NSC19
PK
M7B^OE
Zscore
C
Hypermethylated
Hypomethylated
Number of DMRs
shM7B vs scr
M7B^OE vs PK
GIC19
iNSC19
D
GICshM7B(hyper)
NSC M7B^OE (hypo)
DMPs
GICshM7B vs GICscr LogFC
iNSC M7B^OE vs iNSC PK logFC
GICshM7B(hypo)
NSC M7B^OE (hyper)
E
GICshM7B
(hypomethylated DMRs)
17521
3454
10646
2702 genes
NSC M7B^OE
(hypermethylated DMRs)
GSEA: C2 pathway analysis
Bivalent genes
(H3K4me3, H3K27me3)
H3 acetylation
upon RAR signalling
H3K27me3 marked genes
PRC2 complex
GPCRs
ECM
Immune system
Wnt signalling/beta catenin
PI3K/AKT, P53, ERK/EGF
FDR
0.0
0.05

Figure S8 DNA methylation analysis in GIC and NSC upon METTL7B modulation. Related to Figure 4

A. PCA plots of samples based on M-values of all sufficiently detected probes. Top: Green circle: GIC19 scr replicas (n=4), dark green: centroid of GIC19 scr, Orange triangle: GIC19 shM7B replicas (n=4), dark orange: centroid of GIC19 shM7B, Bottom: Green circle: iNSC19 PK replicas (n=4), dark green: centroid of iNSC19 PK, Yellow triangle: iNSC19 M7B^{OE} replicas (n=4), dark yellow: centroid of iNSC19 M7B^{OE}

B. Heatmaps of beta-values of significantly affected Differentially Methylated Probes (DMPs) in the indicated contrasts. Left: GIC19 shM7B (green) vs GIC19 scr (dark orange), Right: iNSC19 M7B^{OE} (light green) vs iNSC19 control PK (light orange). DMPs with padj < 0.05 and logFC > |2| are illustrated on the heatmap. Colour range purple to yellow indicates high to low methylation.

C. Number of DMRs upon modulation of METTL7B in GIC19 (shM7B) and iNSC19 (M7B^{OE}) as compared to their respective controls (scr, PK).

D. Scatter plot of LogFCs of DMPs (padj<= 0.05). Intersect highlighted in orange: hypermethylated DMPs in GIC19 shM7B vs GIC19 scr and hypomethylated DMPs in iNSC19 M7B^{OE} vs iNSC19 PK. Intersect highlighted in blue: hypomethylated DMPs in GIC19 shM7B vs GIC19 scr and hypermethylated DMPs in iNSC19 M7B^{OE} vs iNSC19 PK

E. Venn diagram (left) of overlapping DMRs in the blue Intersect; hypomethylated DMRs in GICshM7B vs scr and hypermethylated DMRs in iNSC M7B^{OE} vs NSC PK. For hypermethylated regions: maxdiff > 0 & meandiff > 0 & Fisher <= 0.05; For hypomethylated regions: maxdiff < 0 & meandiff < 0 & Fisher <= 0.05. Right: Cytoscape visualization of GSEA: C2 pathway analysis of DMRs in the Intersect GIC hyper / iNSC hypo. Bubbles are coloured based on FDR values and size is proportional to the number of genes included in each C2 term.

Constantinou et al. Figure S9

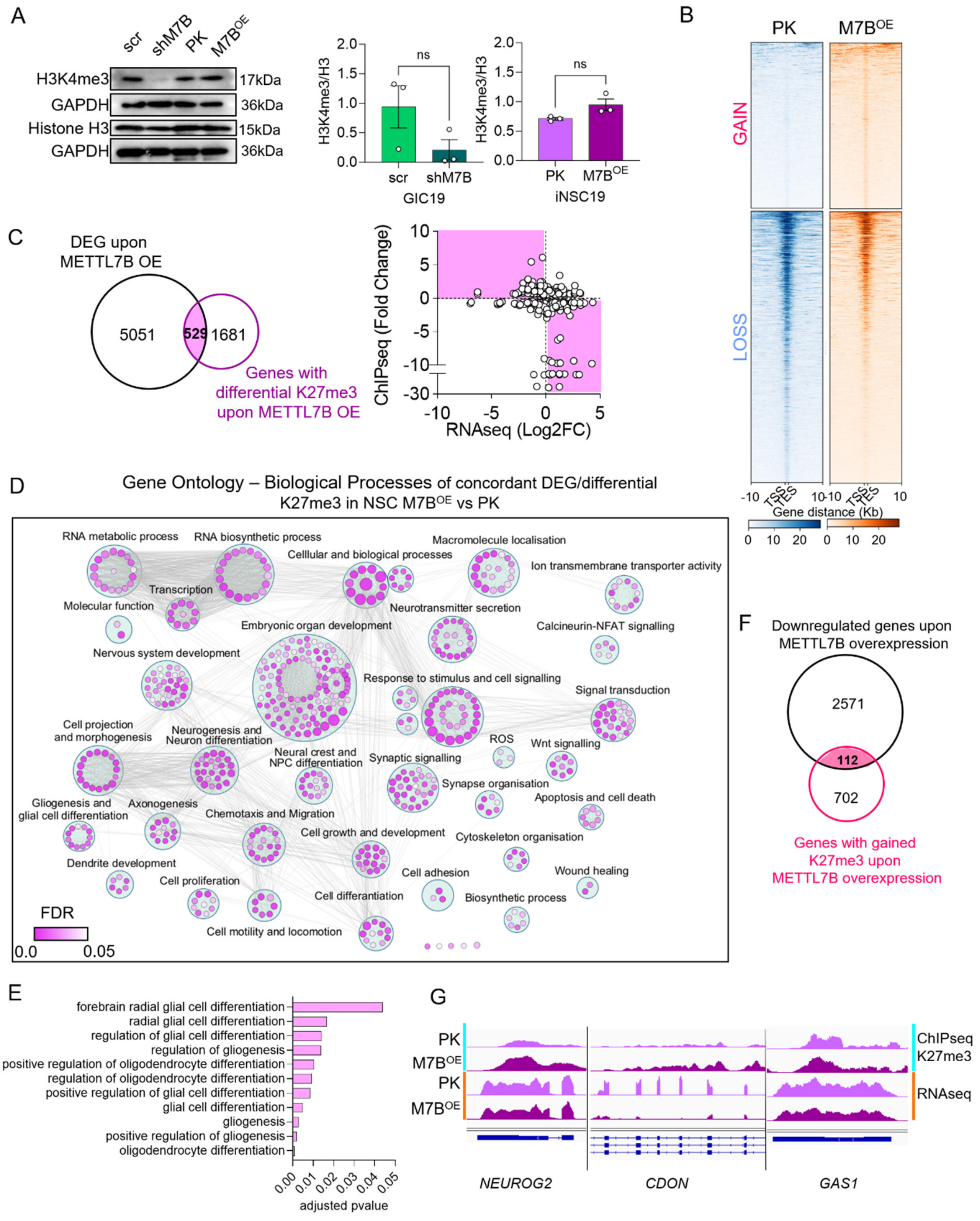

Figure S9 METTL7B epigenetically regulates genes involved in glial cell differentiation in iNSC M7B^{OE}. Related to Figure 5

A. Western blot (left) and quantification (right) of tri-methylation of H3K4/total H3 histone (H3K4me3) in scr (light green) and upon METTL7B silencing (dark green) in GIC19 or in control (PK, light purple) and in METTL7B overexpressing (M7B^{OE}, dark purple) iNSC19. GAPDH immunoreactivity was used to normalise protein loading. n=2 independent experiments, one-way ANOVA. All graphs report mean ± SEM of 3 blots. P values: *P < 0.05, **P < 0.01. Protein expression levels of H3K4me3 and Histone H3 upon silencing of METTL7B in GIC, and overexpression of METTL7B in iNSC
B. Heatmaps of H3K27me3 ChIP-Seq peak enrichments in control (PK, blue) and in METTL7B overexpressing (M7B^{OE}, orange) iNSC19. Heatmaps are centred at transcription start site (TSS) covering +/- 10Kb and ordered by H3K27me3 intensity. Signals were clustered based on the distribution of H3K27me3 surrounding the promoters and divided by genomic loci acquiring (GAIN) or loosing (LOSS) the H3K27me3 mark upon METTL7B overexpression.
C. Venn diagram showing the integration of DE gene from RNAseq and genes presenting differential H3K27me3 peaks from ChIPseq data (left). Volcano plot representing fold changes of expression for DE genes (RNAseq(Log2FC)) and fold change of H3K27me3 peak enrichment (ChIPseq (Fold Change)) upon METTL7B overexpression in iNSC19 (right). Highlighted genes with concordant gain/loss of H3K27me3 peaks and down/upregulation.
D. Bubble plot showing GO biological processes significantly enriched for the 333 concordant genes in iNSC19 upon METTL7B overexpression (highlighted in D, right panel) identified in ChIPseq and RNAseq integration. Bubble are coloured based on FDR values and size is proportional to the number of genes included in each GO term.
E. Histograms showing glial-related GO biological processes significantly enriched for the 333 concordant genes identified in iNSC19 upon METTL7B overexpression in ChIPseq and RNAseq integration.
F. Venn diagram showing the overlap between genes downregulated and with gain of H3K27me3 in iNSC19 upon METTL7B overexpression.
G. Genome browser view showing H3K27me3 ChIP-Seq and RNAseq peaks in NEUROG2, CDON, GAS1 genomic loci in GIC scr (light green) and upon METTL7B silencing (shM7B, dark green) or in iNSC control (PK, light purple) and upon METTL7B overespression (METTL7B^{OE}, dark purple).

Constantinou et al. Figure S10

A

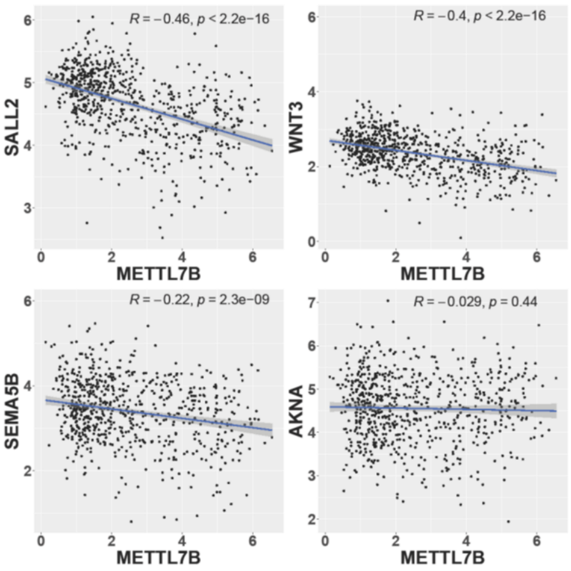

Figure S10 METTL7B expression negatively correlates with neuronal genes in TCGA glioma samples. Related to Figure 5 and 6

A. Correlation of METTL7B and SALL2, SEMA5B, WNT3 and AKNA in TCGA glioma samples.
