# Supplementary material for: Lineage specification in glioblastoma is regulated by METTL7B
Source: Cell Rep. 2024 Jun 5;43(6):114309. doi: 10.1016/j.celrep.2024.114309 (PMC11220825; doi:10.1016/j.celrep.2024.114309)
Supplement: Document S2. Article plus supplemental information [file mmc2.pdf]

Cell Reports

Article

Lineage specification in glioblastoma is regulated by METTL7B

Graphical abstract

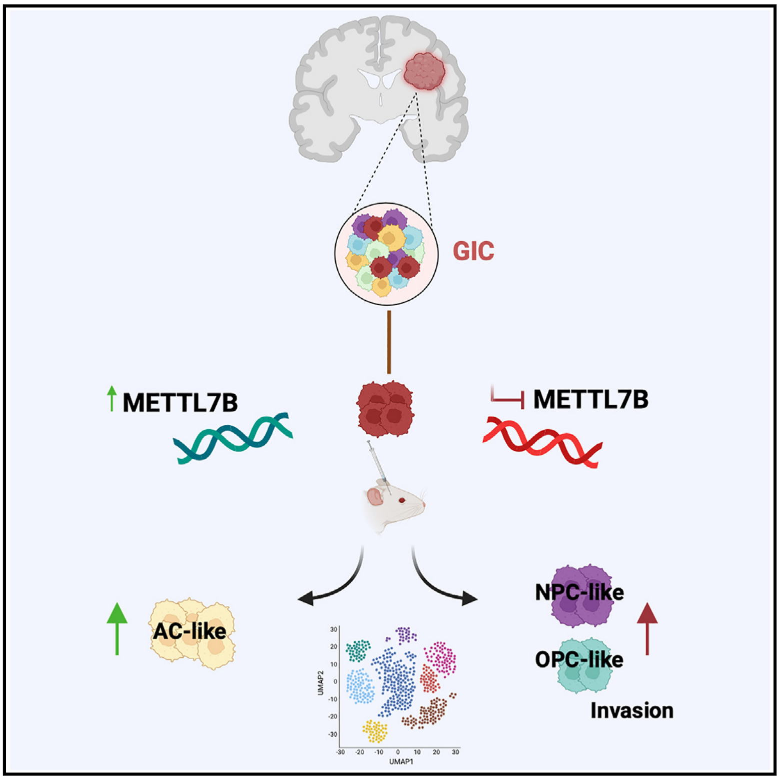

Highlights

- METTL7B is highly expressed in human glioblastoma stem cells
- METTL7B regulates tumor size and invasiveness in an *in vivo* xenograft model
- METTL7B controls the neural stem cell-to-astrocyte differentiation trajectory
- METTL7B regulates SALL2 expression via H3K27me3 modulation

Authors

Myrianni Constantinou, James Nicholson, Xinyu Zhang, ..., Sven Nelander, Sara Badodi, Silvia Marino

Correspondence

s.marino@qmul.ac.uk

In brief

Constantinou et al. identify METTL7B as an essential regulator of lineage specification and a modulator of the expression of the transcription factor SALL2 with wide-ranging impacts on invasion and tumor growth in glioblastoma.

Constantinou et al., 2024, Cell Reports *43*, 114309
June 25, 2024 © 2024 The Authors. Published by Elsevier Inc.
https://doi.org/10.1016/j.celrep.2024.114309

CellPress

Cell Reports

CellPress
OPEN ACCESS

Article

Lineage specification in glioblastoma is regulated by METTL7B

Myrianni Constantinou,[1] James Nicholson,[1] Xinyu Zhang,[1] Eleni Maniati,[2] Sara Lucchini,[1] Gabriel Rosser,[1] Claire Vinel,[1] Jun Wang,[2] Yau Mun Lim,[3] Sebastian Brandner,[3] Sven Nelander,[4] Sara Badodi,[1] and Silvia Marino[1,5,*]

[1]Brain Tumour Research Centre, Blizard Institute, Barts and The London School of Medicine and Dentistry, Queen Mary University London, London, UK

[2]Barts Cancer Institute, Barts and the London School of Medicine and Dentistry, Queen Mary University of London, London EC1M 6AS, UK

[3]Division of Neuropathology, The National Hospital for Neurology and Neurosurgery, University College London Hospitals NHS Foundation Trust, and Department of Neurodegenerative Disease, Queen Square, Institute of Neurology, University College London, Queen Square, London, UK

[4]Department of Immunology Genetics and Pathology, Uppsala University, Uppsala, Sweden

[5]Lead contact

*Correspondence: s.marino@qmul.ac.uk

https://doi.org/10.1016/j.celrep.2024.114309

SUMMARY

Glioblastomas are the most common malignant brain tumors in adults; they are highly aggressive and heterogeneous and show a high degree of plasticity. Here, we show that methyltransferase-like 7B (METTL7B) is an essential regulator of lineage specification in glioblastoma, with an impact on both tumor size and invasiveness. Single-cell transcriptomic analysis of these tumors and of cerebral organoids derived from expanded potential stem cells overexpressing METTL7B reveal a regulatory role for the gene in the neural stem cell-to-astrocyte differentiation trajectory. Mechanistically, METTL7B downregulates the expression of key neuronal differentiation players, including SALL2, via post-translational modifications of histone marks.

INTRODUCTION

Glioblastoma isocitrate dehydrogenase (IDH)-wild type is the most common intrinsic malignant brain tumor in adults; it is characterized by poor prognosis due to the rapid development of resistance to the current therapy. Cells with stem cell properties, known as glioblastoma-initiating or stem cells (here defined GICs), have been shown to contribute to the high intra- and intertumoral heterogeneity as well as the plasticity of these neoplasms, hence providing an interpretative frame for the variable responses of these tumors to therapies and their inevitable recurrence. Epigenetic mechanisms of regulation of gene expression, defined as those hereditable mechanisms impacting gene activity without changes to the DNA sequence, control stem cell functions, including self-renewal and lineage specification/differentiation during embryonic development and maintenance of homeostasis in adult organs. The contribution of deregulation of these mechanisms to glioblastoma growth, mostly via regulating its heterogeneity and plasticity, has been well characterized.[1–3] However, intriguingly, chromatin structure leading to consistent transcriptional regulatory programs across patients with glioblastoma has also been identified in tumor tissues,[4] raising the possibility that these epigenetic regulators could be attractive targets for the development of therapeutic approaches. It is, however, of fundamental importance that we can identify those regulatory programs that distinguish GICs from neural stem cells (NSCs) to enable the selective exploitation of these vulnerabilities and limit toxicities.

The methyltransferase-like (METTL) family of proteins is a subfamily of seven-beta-strand methyltransferases comprising 33 members in vertebrates. They can methylate DNA, RNA, protein residues, or alkyl thiol groups, although it is unclear whether additional, not yet characterized functions also exist. METTL proteins have been implicated in a wide range of biological processes (BPs), including regulating stem cell development and oncogenesis.[5] In glioblastoma, the role of METTL3 in regulating mitophagy,[6] tumor maintenance, and drug resistance[7,8] via RNA m6A methylation[9] has been shown, and one study described a role for METT7B in glioblastoma progression, potentially via the regulation of EGR1.[10]

Here, we identified METTL7B as an essential regulator of lineage specification and an epigenetic modulator of the expression of the transcription factor SALL2 with wide-ranging impact on invasion and tumor growth in glioblastoma.

RESULTS

METTL7B is an influential gene distinguishing neoplastic stem cells from neural stem cells in glioblastoma

We leveraged the transcriptomic data from our SYNGN platform, a collection of 10 GIC and syngeneic expanded potential stem cell (EPSC)-derived NSC (iNSC) pairs,[11] to identify the genes that are most influential in distinguishing GICs from NSCs. Biplots were used to visualize both samples and weightings of the first two components of a principal-component analysis

© 2024 The Authors. Published by Elsevier Inc.
This is an open access article under the CC BY license (http://creativecommons.org/licenses/by/4.0/).

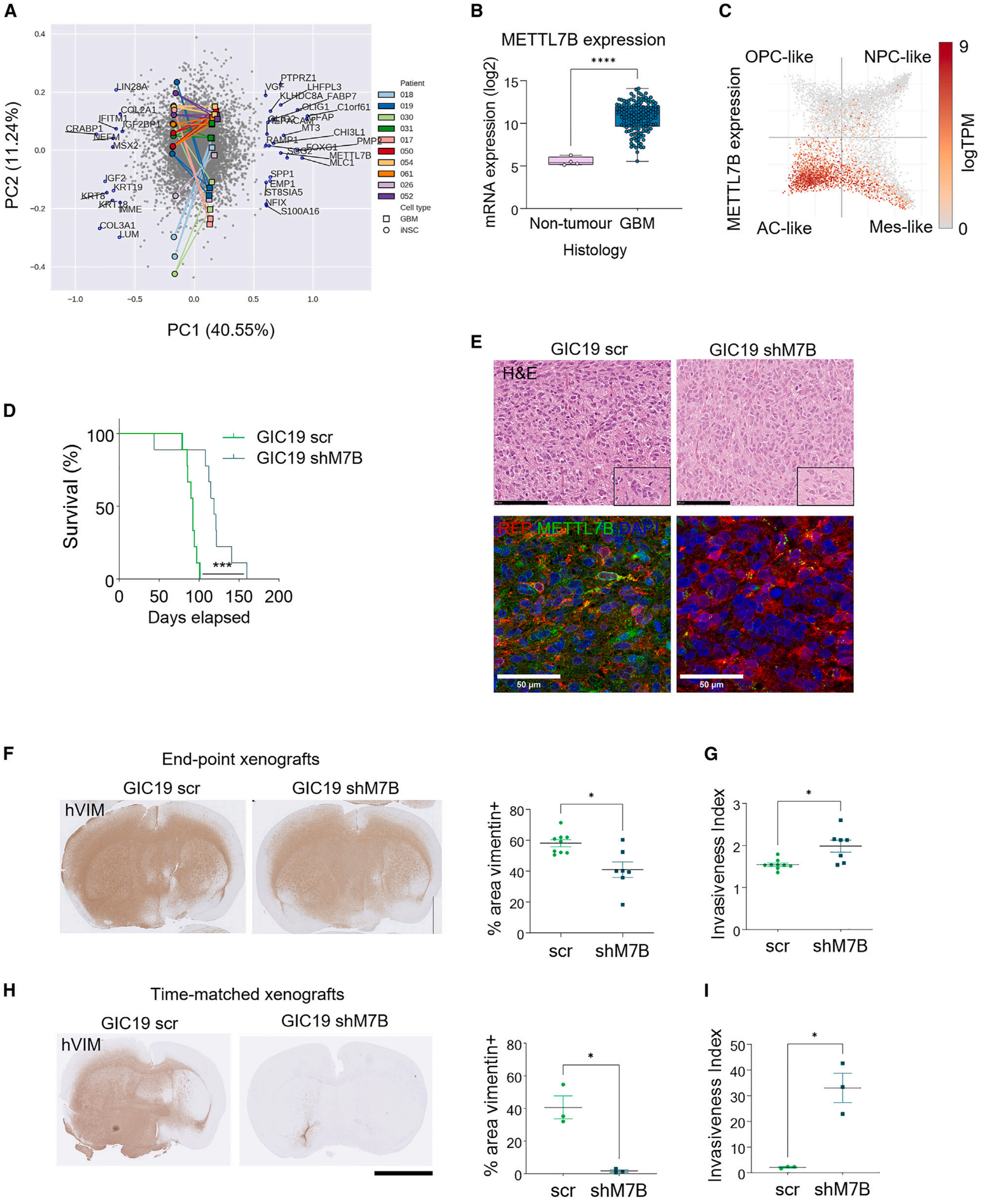

Figure 1. METTL7B is expressed in GICs and contributes to glioblastoma growth in a xenograft model

(A) PCA biplot visualization of gene expression data. x axis: glioblastoma and iNSCs. y axis: individual patients. Genes on the right are upregulated and those on the left are downregulated in all GICs as compared to iNSCs. 10 SYNGN GIC/iNSC pairs, $n = 2$ (independent passages) per cell line.

(legend continued on next page)

(PCA) to identify differentially expressed genes (DEGs) with the highest loading, i.e., those genes pushing the GIC and iNSC clusters apart most strongly (Figure 1A). The 37 most influential genes, of which 23 were upregulated and 14 downregulated in GICs, were further studied, and in particular, their association with glioblastoma, as assessed by literature review, was analyzed to validate our comparative approach. Several well-characterized genes associated with GIC maintenance, MSX2/SOX2[12] and FOXG1,[13] and glioblastoma invasion/progression, MLC1[14] and VGF,[15] were identified.

The approach also identified genes, the role of which was less characterized or unexplored in glioblastoma. METTL7B caught our attention, as it has been shown to be expressed and linked to poor prognosis in low-grade glioma,[17] but its role in glioblastoma and normal brain development is not well characterized. Increased gene expression at the protein level was confirmed in two lines from our SYNGN cohort, GIC19 and GIC61 (see Vinel et al.[11] for clinical and molecular details of these lines) (Figures S1A and S1B). We also confirmed increased expression in unrelated lines from a repository (Figure S1C) and in glioblastoma samples from TCGA as compared to non-tumor tissue (Figure 1B) or IDH-mutant gliomas (Figure S1D). The classical and mesenchymal subtypes expressed significantly higher levels of METTL7B as compared to the proneural subtype (Figure S1E), and METTL7B expression correlated with worse survival in patients with IDH-wild-type glioma in TCGA and CGGA databases (Figure S1F). At the spatial level, METTL7B expression was higher in the infiltrating tumor (intermediate zone between leading edge and cellular tumor, ratio of tumor to normal cells: 10–20/100) and cellular tumor (tumor core) as compared to the leading edge (border of the tumor, ratio of tumor to normal cells: 1–3/100) (Ivy Glioblastoma Atlas Project, Figure S1G). At the single-cell transcriptomic level, higher expression was found in adult glioblastomas (Figure S1H), and within the four states originally identified in these tumors (astrocyte [AC]-like, oligodendrocyte progenitor cell [OPC]-like, mesenchymal [Mes]-like, and neural progenitor cell [NPC]-like), the gene was highly expressed in the AC-like cells, with moderate expression also noted in the Mes-like cells (Figure 1C).

METTL7B expression characterizes neoplastic stem cells as compared to iNSCs, and its highest expression levels are found in the AC-like cell state in glioblastoma.

METT7LB controls tumor size and invasion in an *in vivo* model of glioblastoma, and its silencing leads to enhanced neuronal differentiation

Next, we set out to assess whether silencing of the gene in GICs would have a functional impact on tumor formation and progression. Short hairpin RNA (shRNA)-mediated knockdown of METTL7B (three independent shM7B-a, -b, and -c) was implemented in GIC19 and GIC61 (referred to as GICshM7B) and confirmed at the protein level (Figure S1I), followed by intracerebral injection of GIC19 (scramble [scr] and shM7B-c) into the forebrain of recipient mice, which were then maintained on tumor watch until symptoms arose. Mice xenografted with GIC19shM7B survived significantly longer than controls (Figure 1D). Histological analysis at endpoint revealed highly pleomorphic glial neoplasms with high mitotic activity and vascular proliferation, in keeping with glioblastoma in both control and METTL7B-deficient xenografts (Figure 1E, top). Immunofluorescence staining confirmed the sustained silencing of METTL7B in the xenografts (Figure 1E, bottom). Tumor size, as assessed by the total area occupied by cells immunolabelled against human vimentin (hVIM), was significantly reduced in the xenografts derived from the mutant line (18.3%–60.3% vs. 51.8%–71.4%, Figure 1F). Increased apoptosis, as assessed by cleaved caspase-3 staining, was also observed (Figure S1J), while no significant change in proliferation rate, as assessed by Ki67 staining, was detected (Figure S1K). These findings are in keeping with our observations in both GIC lines cultured in 2D upon METTL7B silencing with independent shRNA (Figures S1L and S1M). Glioblastoma extensively invades the surrounding brain tissue; hence, we set out to assess invasion properties upon METTL7B silencing. We took advantage of an unbiased image analysis method (invasiveness index) which measures the tumor cell dispersion outside of the tumor core as assessed by the ratio of the gross tumor area to the tumor core area.[18] Intriguingly, the mutant tumors showed a higher propensity for invasion into the surrounding brain (1.4–2.1 for shMETTL7B, 1.5–1.8 for scr, Figures 1G and S2A). Since GIC19shM7B patient-derived xenograft (PDX) mice showed significantly longer survival, we reasoned that a comparison of tumors at endpoint might mask the extent of some of the phenotypic changes. Thus, we set up a new cohort of xenografted animals and harvested time-matched mouse brains from asymptomatic GICshM7B mice

(B) *METTL7B* expression in 156 bulk glioblastoma samples as compared to healthy tissue. All graphs report mean ± SEM. Two-tailed unpaired t test, ***$p < 0.001$.
(C) scRNA-seq data from Neftel et al.[16] plotted in a 2D cell state plot. Quadrants: AC-like (bottom left), MES-like (bottom right), OPC-like (top left), and NPC-like (top right). *METTL7B* log transcripts per million (TPM) expression ranging from light orange to dark.
(D) Kaplan-Meier survival curves of mice xenografted with GIC19scr (black) and GIC19shM7B (green) ($n = 9$ animals per group), two-tailed p values determined by log-rank (Mantel-Cox) test.
(E) Top: H&E representative pictures of intracerebral xenografts derived from GIC19 scr and shM7B. Microvascular proliferations are shown in the inset. Scale bar: 100 μm. Bottom: immunostainings (immunohistochemistry [IHC]) with METTL7B antibody in xenografts derived from GIC19scr and GICshM7B. Scale bar: 50 μm.
(F) IHC for human vimentin (hVIM) in brains of mice xenografted with GIC19scr and GICshM7B at endpoint and quantification of tumor area shown as percentage (%) of vimentin-positive cells in the overall area. Scale bar: 5 mm. All graphs report mean ± SEM. Welch's t test, *$p = 0.03$. Light green: scr, $n = 9$ animals; dark green: shM7B, $n = 7$ animals.
(G) Invasiveness index of endpoint xenografts. Welch's t test, *$p = 0.02$. Light green: scr, $n = 9$ animals; dark green: shM7B, $n = 7$ animals.
(H) IHC for hVIM in time-matched GIC19scr and GICshM7B xenografted mouse brains. Light green: scr, $n = 3$ animals; dark green: shM7B, $n = 3$ animals. Scale bar: 5 mm. All graphs report mean ± SEM. Welch's t test, *$p = 0.03$.
(I) Invasiveness index of time-matched xenografts. Light green: scr, $n = 3$ animals; dark green: shM7B, $n = 3$ animals. Welch's t test, *$p = 0.03$.
See also Figures S1 and S2.

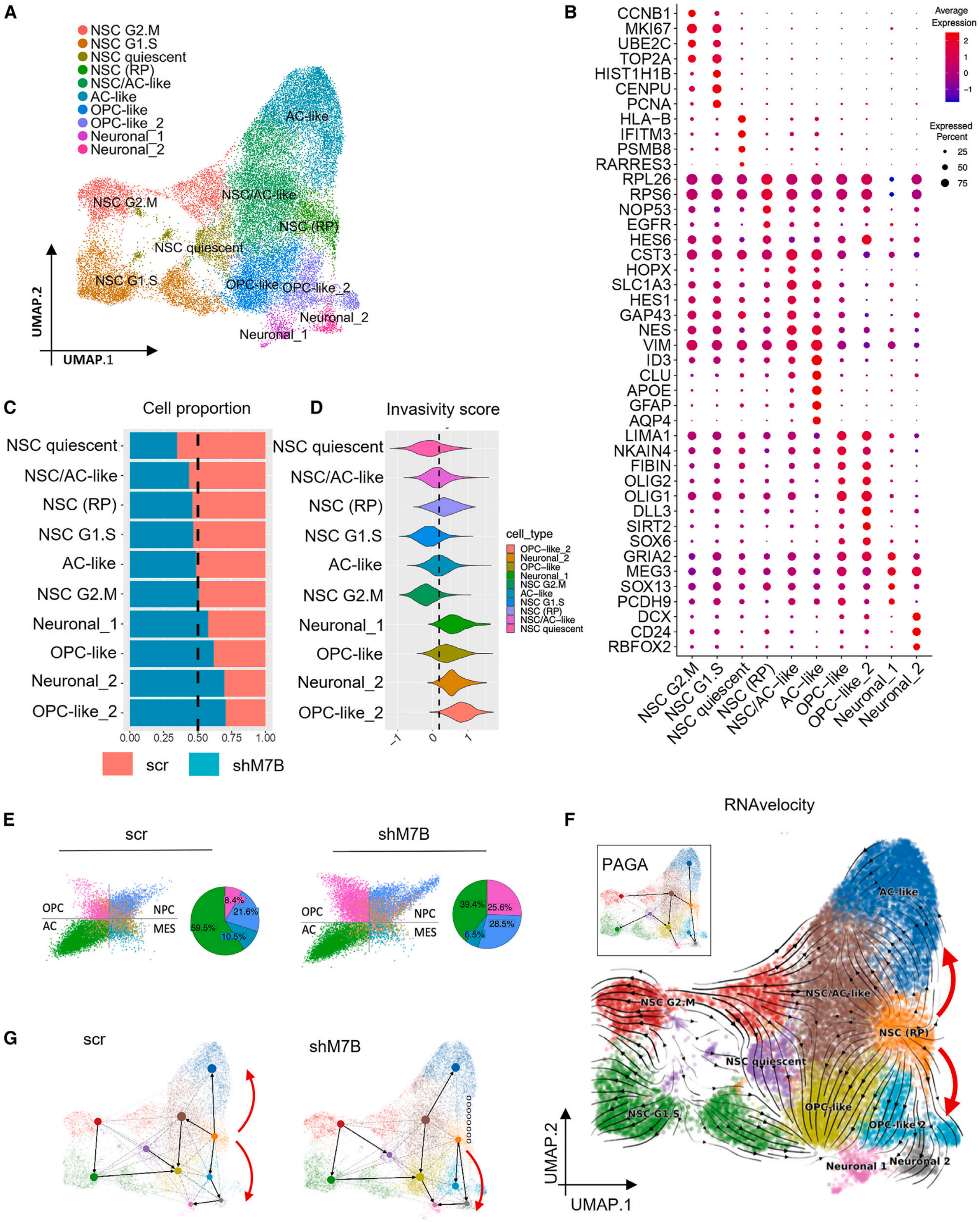

(legend on next page)

when the brains of GICscr mice had to be collected because the mice developed symptoms. Staining for hVIM (Figure 1H) and quantification of the tumor area revealed significantly reduced tumor size (0.73%–3.1%) in the GICshM7B brains when compared to the controls (32.1%–54.7%). Importantly, the invasion of GICshM7B in the surrounding tissue was significantly higher than the controls (22.9–42.6 shM7B, 1.6–2.4 scr, Figures 1I and S2B) also at this early time point and >15-fold higher than endpoint GICshM7B xenografts. To validate our histological finding of increased invasion upon METTL7B silencing, we used two complementary *in vitro* assays for migration/invasion, the "transwell" and "scratch" assays (Figures S2C and S2E). In agreement with the increased invasivity in the shM7B xenografts, shM7B cell lines showed increased invasion in both assays, and importantly, this phenotype was reproduced also in the GIC61 cell line (Figures S2D and S2F).

To characterize the role of METTL7B in glioblastoma xenografts at the cellular and molecular levels, we performed single-cell RNA sequencing (scRNA-seq) on GIC19shM7B (n = 3) and GIC19scr (n = 2) tumors, harvested at the onset of symptoms. After computational exclusion of murine cells and stringent filtration (see STAR Methods), 28,415 high-quality single cells were available for analysis. We performed dimensionality reduction and Louvain clustering before aggregating clusters into cell types based on their shared expression of well-known marker genes (Figures 2A and 2B). This identified cell types reminiscent of the cellular hierarchy of the developing brain, including neural stem cell (NSC)-like clusters (NES, HES1) and AC-like clusters (HOPX, AQP4, glial fibrillary acidic protein [GFAP]), as well as cells with an OPC-like (OLIG1/2) or neuronal (GRIA2/DCX) gene expression pattern. A comparison of the relative cell-type proportions revealed a decrease in NSC quiescence and an increase in OPC-like2/neuronal_2 clusters in the shM7B condition (Figure 2C), a consistent finding in all tumor replicates (Figure S3A). For both enriched populations, we performed differential gene expression analysis between the two conditions (Figure S3B). In each case, there was a strong downregulation of the astrocytic/glial gene expression program (PTPRZ1, CST3, VIM), with the neuronal_2 cells upregulating neuronal-specific genes (STMN2, NNAT, DLX5, DLX6-AS1) and the OPC-like_2 cells upregulating a mixture of genes associated with the oligodendrocytic and neuronal fates (NEU4, DLL3, NNAT, PLP1). Gene set enrichment analysis (GSEA) for the DEGs revealed enrichment for the metabolism of RNA, amino acids, and proteins, as well as nervous system development including axon guidance, Roundabout (ROBO) signaling pathways, and tubulin folding in the neuronal_2 cluster (Figure S3C). Among the most significantly deregulated Reactome pathways and Gene Ontology Biological Processes (GOBPs) in the OPC-like_2 clusters, axon guidance and ROBO signaling, as well as key signaling pathways (p53, Phosphatase And Tensin Homolog [PTEN], mitogen-activated protein kinase [MAPK], NOTCH, Glioma-associated oncogene [GLI], Hedgehog [Hh]) and glial cell differentiation, were affected (Figure S3D).

Recent work has drawn an association between neuronal signaling and invasion in GICs.[19] Consistent with this theory, we found that the shM7B-enriched OPC-like_2 and neuronal_2 clusters showed a significantly increased invasivity score (Figure 2D), suggesting that this population may underpin the increased invasion observed in shM7B xenografts (Figures 1F and 1G). Increased expression levels of migration/invasion-related markers TGFB1,[20,21] SPP1,[22] MMP2,[23] and NLGN3[24] were detected in the PDX-derived shM7B cells (Figure S3E). GO analysis of upregulated genes between the two groups revealed an increase in BPs related to migration, invasivity, and neuronal identity (Figure S3F).

Gene signature scoring revealed that our xenograft-derived glioblastoma cells closely recapitulated the cellular states derived from patient glioblastoma,[16] with noticeable decrease of the AC-like state from 59.5% in scr to 39.4% in shM7B and an increase of the NPC-like state from 21.6% to 28.5% (Figures 2E and S4A–S4C). To analyze how well our PDX-based findings reflected glioblastoma tumors in patients, we used a recently published cohort of scRNA-seq data (Wang et al.[25]). We first separated samples into METTL7B-high and METTL7B-low groups based on median expression and then used signature scoring to evaluate their cell state compositions (Figure S4D). Consistent with our findings, we observed a significant decrease in the proportion of AC-like cells as well as an increase in the proportion of NPC-like cells in the METTL7B-low group (Figure S4E).

We next queried whether alterations in lineage specification governed by METTL7B knockdown were responsible for the increased proportion of invasive neuronal/OPC-like cells observed in shM7B tumors. RNAvelocity analysis is best suited to developmental analyses but can also provide useful information in cancer when cells adopt pseudo-developmental hierarchies.[26,27] Here, we observed two broad vector fields from NSCs suggesting differentiation toward either AC-like or neuronal/OPC-like cells (Figure 2F). Partition-based graph

Figure 2. METTL7B silencing polarizes lineage specification away from the astrocytic fate toward neuronal/oligodendrocytic phenotypes

(A) Uniform manifold approximation and projection (UMAP) plot of xenograft samples (GICshM7B [n = 3], GICscr [n = 2]) with cells colored by annotated cell type.

(B) Dot plot of cluster marker gene expression used for cell-type annotation. x axis: cell clusters; y axis: marker genes. Average gene expression ranges from low (blue) to high (red), and dot size is proportional to the percentage of cells expressing the genes.

(C) Cell-type proportions from GICscr vs. GICshM7B samples. Orange, GICscr; blue, GICshM7B. Dashed line marks 50%.

(D) Patient-derived GIC cell state (OPC-like, AC-like, NPC-like, MES-like) distribution in the scr and shM7B samples (quadrant plot as defined in Neftel et al.[16]). Pie charts depict the percentages of the four cellular states in each group.

(E) Violin plot showing cell-type enrichment of glioma invasiveness gene set.

(F) RNA velocity streamlines projected onto UMAP embeddings shows a split vector field from NSCs (RP) toward either AC-like cells or neuronal/OPC-like cell types.

(G) UMAPs overlaid with PAGA graph abstractions calculated on scr samples (left) or GICshM7B samples. Weighted edges correspond to the connectivity between two clusters, and directionality is RNAvelocity inferred.

See also Figures S3 and S4.

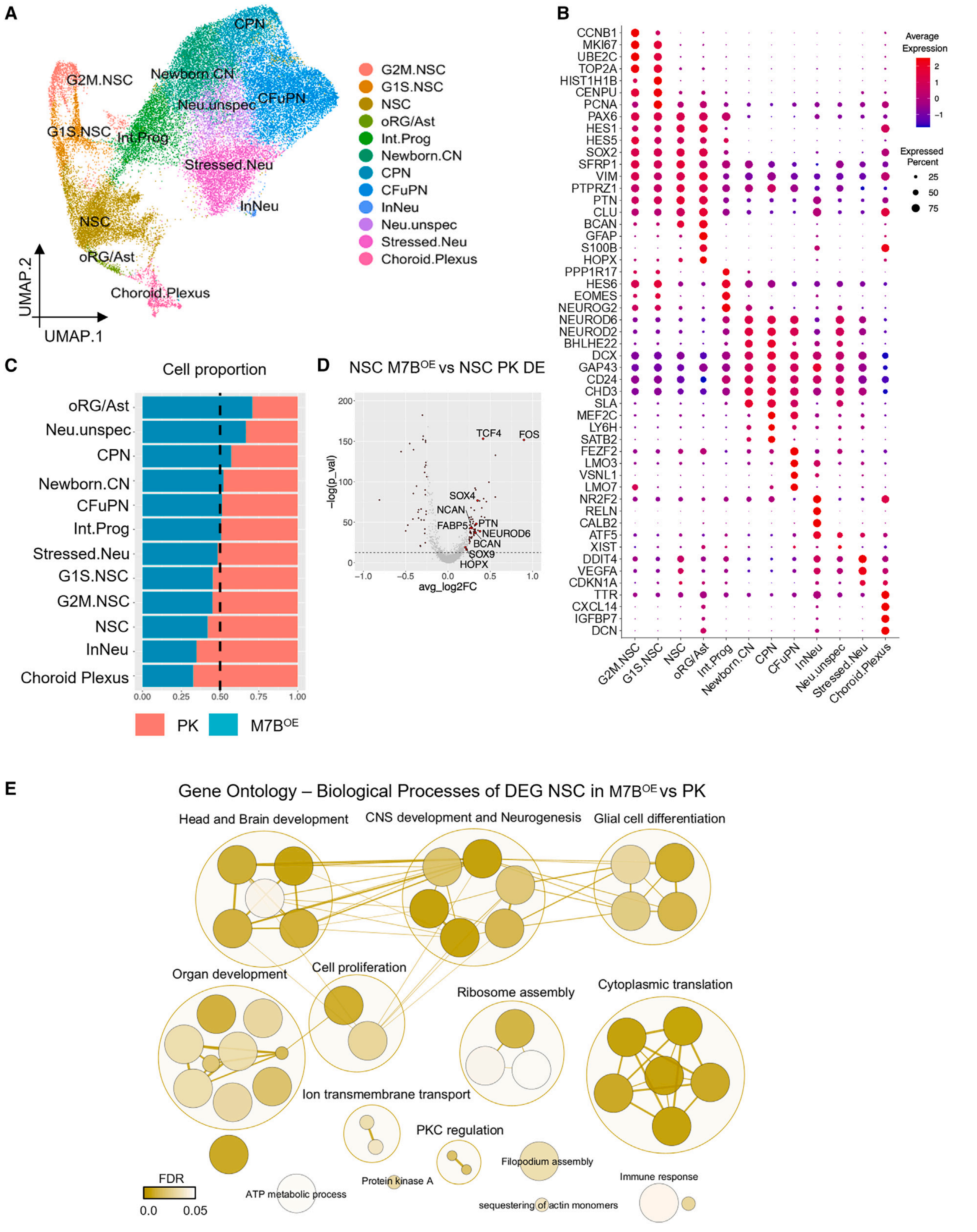

(legend on next page)

abstraction (PAGA)-RNAvelocity analysis predicts probable transitions between clusters/cell types,[28] and analysis on either the full (Figure 2F, inset) or the scr-only cells (Figure 2G) indicates that the NSC ribosomal protein (RP) cluster of NSCs—defined in part by a previously described[29] high ribogenesis signature—can transition either into AC-like or OPC/neuronal-like clusters. In contrast, PAGA analysis on shM7B cells only shows a loss of the NSC (RP)→AC-like trajectory (Figure 2G), suggesting that in the context of METTL7B knockdown, lineage specification is skewed away from AC-like cells toward a more neuronal/OPC lineage.

Taken together, our results indicate that METTL7B silencing leads to smaller but more invasive tumors in xenografts by polarizing linage specification away from the astrocytic fate and toward more invasive neuronal/oligodendrocytic cells.

METT7LB regulates lineage specification during brain development

To further characterize the role of METTL7B in lineage specification, we decided to use the cortical development paradigm, given the strong cellular and molecular links between glioblastoma and various neural stem/progenitor cells that give rise to the adult forebrain. Leveraging the anatomic RNA-seq data on the BrainSpan Atlas, part of the Allen Brain Atlas, we observed that the expression of METTL7B was overall low during early development but increased sharply at 37 post-conception weeks (Figure S5A), when human fetal neurodevelopment is at one of its most crucial phases, as it undergoes cortical patterning, gliogenesis, myelination, and extensive synaptogenesis.[30] Expression after birth and until adulthood fluctuated, with high expression observed mainly in the motor and somatosensory cortex (Figure S5A). We next used a recent scRNA-seq dataset of fetal neurodevelopment to interrogate cell-type-specific METTL7B expression during mid-gestation (gestational weeks 17–19).[31] This identified the expression of METTL7B in outer radial glia cells (oRGs) and astrocytes (ACs), with the highest level of expression in glial-committed progenitors (Figure S5B). In cerebral organoids (COs), expression of METTL7B was found in the oRGs at 3 months,[32] and expression was further increased in this cluster at 6 months, with expression now also detected in the astroglial cell clusters (Figure S5C).

To explore the functional impact of increased METTL7B expression in the developing brain and ask the question of whether it would initiate tumor formation, we engineered EPSC lines to overexpress the gene via lentiviral-mediated transduction and validated them at the RNA and protein levels by RT-qPCR (Figure S5D) and western blot (Figure S5E), respectively. COs were derived from two EPSC lines overexpressing METTL7B (CO19, EPSC19-derived COs and CO61, EPSC61-derived COs) and maintained for up to 70 days post-embryoid formation initiation. The average diameter of the CO was not affected by the overexpression of METTL7B (Figure S5F), and histological analysis revealed neural rosettes with a central lumen and well-defined cortical layering in both wild-type and METTL7B-overexpressing COs (Figure S5G). Tumor growth was not observed, a finding confirmed also in a soft agar assay performed with 70-day-old COs dissociated into single-cell suspension, which did not give rise to colony formation in a crystal violet staining performed 3 weeks after plating (Figure S5H). Intracerebral injection of iNSC19 PKAM (PK) and iNSC19M7B^{OE} (Figure S5I) in recipient NOD SCID mice did not result in tumor formation at up to 8 months of observation, as assessed by histological analysis including immunostaining for hVIM (Figure S5J).

To explore if METTL7B overexpression may cause a more subtle phenotype, scRNA-seq was carried out on COs derived from both METTL7B-overexpressing EPSC lines (M7B^{OE}) and controls (PK). After stringent filtration, 37,575 high-quality cells were retained for analysis. We performed dimensionality reduction and Louvain clustering (Figure S6A) before comparing cluster-defining marker genes to cell-type signatures derived from two large single-cell resources of brain organoid development (Figure S6B[32,33]). Clusters were then aggregated and annotated into cell types on the basis of shared expression of marker genes and correlations to reference datasets (Figures 3A and 3B). A shift in lineage specification was observed (Figure 3C); most notably, the small cluster of outer radial glia/Astrocytic cells (HOPX, GFAP, BCAN, S100B) was enriched for in the M7B^{OE} cells. Other cell populations including NSCs (PAX6, SOX2, HES1), intermediate progenitors (EOMES, NEUROG2), and newborn and more mature neuronal cells (DCX2, neuronal differentiation [NEUROD]2, MEF2C, LMO3/7) remained unchanged. However, there was an increase in the proportion of unspecified neurons, which retained an overall neuronal signature but lacked the expression of markers of more differentiated neuronal types. Instead, among their few marker genes was ATF5, which plays a neuroprotective role during endoplasmic reticulum stress,[34] and XIST, which is upregulated across a wide range of cancers, including glioblastoma.[35] The increase in these unspecified neurons suggests that M7B^{OE} compromises normal CO neuronal development. The two populations that were depleted of M7B^{OE} cells were inhibitory neurons (RELN, CALB2) and the choroid plexus (TTR, CXCL14, IGFBP7), both of which are typically of low and variable abundances in COs.

Figure 3. METTL7B regulates lineage specification in COs toward an oRG/Astrocytic phenotype

(A) UMAP plot of CO samples (CO19PK, CO19M7B^{OE}, CO61CAS9PK, CO61M7B^{OE}) with cells colored by annotated cell type.

(B) Dot plot of cluster marker gene expression used for cell type annotation. x axis: cell clusters; y axis: marker genes. Average gene expression ranges from low (blue) to high (red), and dot size is proportional to the percentage of cells expressing the genes.

(C) Cell proportions in control COs vs. COM7B^{OE}. Orange, CO; blue, COM7B^{OE}. Dashed line marks 50%.

(D) Volcano plot of DEGs in COM7B^{OE} as compared to control in the NSC cluster. x axis: average log2 fold change (avg_log2FC); y axis: $-\log(p\_val)$. Upregulated genes in COM7B^{OE}: avg_log2FC > 0. Downregulated genes in COM7B^{OE}: avg_log2FC < 0.

(E) GSEA for GOBPs of DEGs in the NSC cluster, visualized on Cytoscape. Bubbles are colored based on false discovery rate (FDR) values, and size is proportional to the number of genes included in each GO term.

See also Figures S5 and S6.

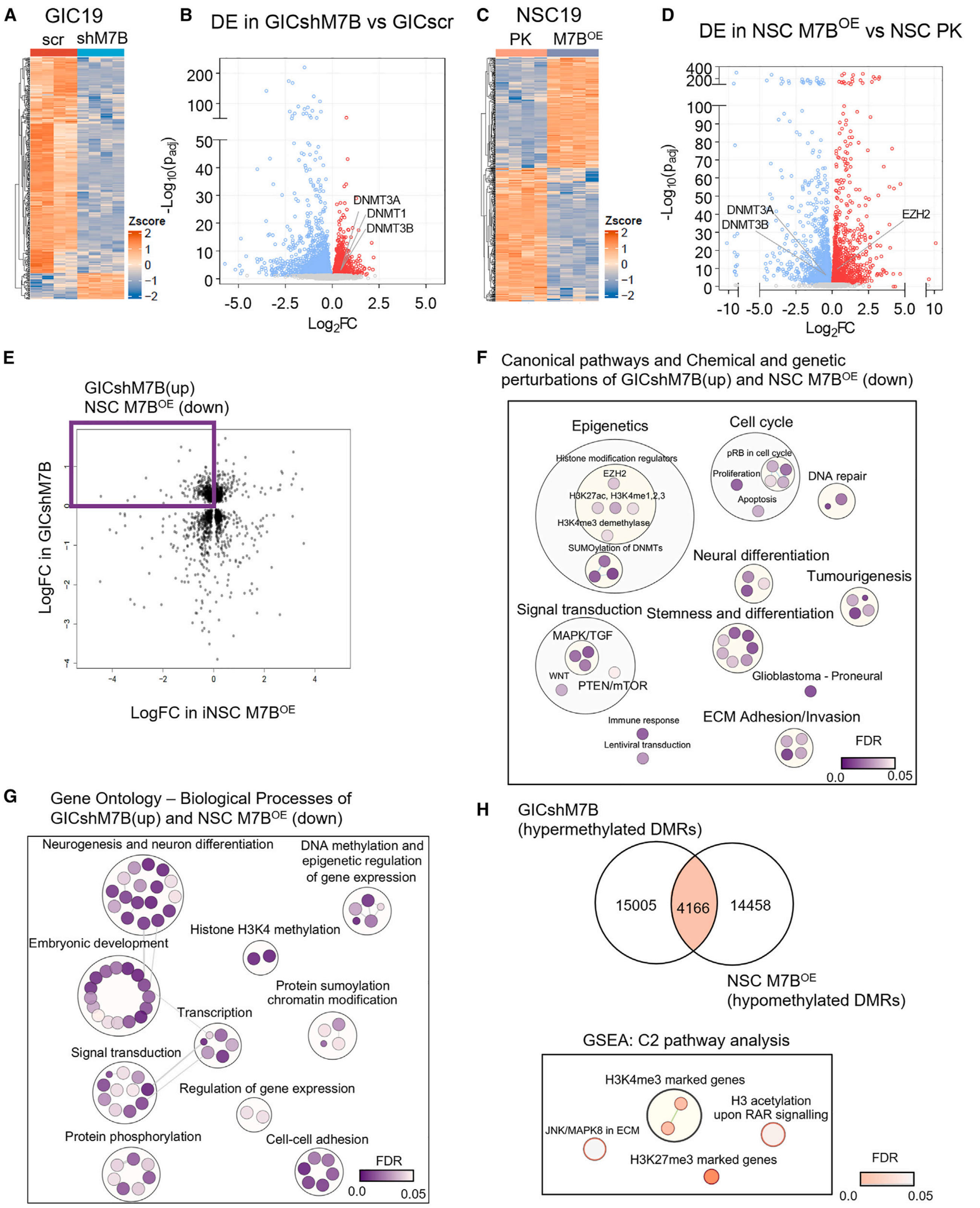

(legend on next page)

Of note, the observed changes in lineage specification and cell-type proportions were greater in EPSC61-derived COs, which may indicate some degree of cell line specificity; however, the direction of cell-type proportion changes remained consistent in EPSC19-derived COs (Figure S6C). In line with our *in vitro* data (Figure S5F), cell cycle scoring did not detect any METTL7B-induced changes to cell proliferation (Figure S6D).

Interestingly, despite the changes to neuronal and glial lineage specification, the relative proportion of NSCs remained largely consistent in both conditions. We therefore reasoned that METTL7B-driven gene expression changes in the NSC population might prime them toward a more glial fate, thus perturbing normal neuronal differentiation. In keeping with this theory, differential expression within the NSC population (Figure 3D) revealed an increase in genes associated with a more glial phenotype (TCF4, PTN, FABP5, BCAN). It is also noteworthy that some genes associated with a more neuronal fate (SOX4, NEUROD6) were also upregulated, suggesting that some M7B^{OE} NSCs might aberrantly express both neuronal and glial programs, and this could potentially explain the observed increase in the cluster of poorly differentiated unspecified neurons. GO enrichment for these genes confirmed deregulated pathways associated with CNS development and neurogenesis, glial differentiation, neuron migration, cell proliferation, and cytoplasmic translation (Figure 3E).

In conclusion, METTL7B is expressed in the outer radial glia, glial-committed progenitors, and ACs during brain development, and its overexpression in CO enhances astroglial differentiation without inducing neoplastic transformation.

METTL7B influences DNA methylation and modulates the expression of methyltransferases

METTL7B silencing in GICs significantly impacted lineage specification and tumor development upon intracerebral injection in mice; scRNA-seq analysis of COs derived from EPSCM7B^{OE} revealed a distinct and complementary lineage specification phenotype. Therefore, we set out to further study the molecular underpinnings of METTL7B role by analyzing the transcriptome of GICshM7B and iNSCM7B^{OE}. We reasoned that this *in vitro* tool would be amenable to a wider range of exploratory mechanistic analyses of the epigenetic regulation of gene expression as well as functional validation in a syngeneic setting, hence excluding the potentially confounding effect of different background genetics.

Unsupervised clustering of GIC19scr and shM7B (n = 4 replicas from independent infections) samples based on global gene expression showed separation of the two groups in PCA plots (Figure S7A), and a total of 3,625 significantly affected DEGs were identified between the two groups (1,653 genes upregulated and 1,972 downregulated, Figure 4A). Among the downregulated genes, *METTL7B* was detected, confirming its successful knockdown in our samples. Among the significantly upregulated genes, other members of the METTL family (*METTL7A*, *METTL3*) and several DNA methyltransferases (*DNMT1*, *DNMT3A*, and *DNMT3B*) caught our attention (Figure 4B). Next, we used GSEA on all genes in the dataset ranked by a differential expression statistic (t-statistic) to identify GOBPs and canonical pathways (CPs) associated with these genes. Among the 50 most significantly (adjusted p value [$p$adj] < 0.05) affected GOBPs (25 upregulated, mean normalized enrichment score [NES] > 0; 25 downregulated, NES < 0), several histone- and lysine-modification-related processes were upregulated, while extracellular matrix (ECM)-related wound-healing processes were downregulated (Figures S7B and S7C). Upregulated CPs included histone and lysine modifications, histone acetylation by histone acetyltransferases (HATs), sumoylation, and epigenetic regulation of gene expression (Figure S7D). Among the downregulated CPs, we identified ECM-related pathways, immune response, and key signal transduction pathways, as well as differentiation and neurogenesis-related pathways (Figure S7E), in keeping with the results of our *in vitro* functional assays and phenotypic characterization of xenografted cells.

Figure 4. METTL7B is an epigenetic regulator of DNA methylation and histone modification marks

(A) Heatmap of normalized gene expression of DEGs in GIC19shM7B (light blue column annotation bar) as compared to GIC19scr (red column annotation bar). 479 DEGs are shown (log2FC |1| and $p$adj ≤ 0.05). Color range from orange to blue indicates high to low gene expression.

(B) Volcano plot of all DEGs in GIC19shM7B as compared to GIC19scr. x axis, log2FC; y axis, −log10($p$adj). Red dots: upregulated genes (log2FC > 0) in GIC19shM7B as compared to scr with $p$adj ≤ 0.05. Blue dots: downregulated genes (log2FC < 0) in GIC19shM7B as compared to scr with $p$adj ≤ 0.05. Gray: non-significant.

(C) Heatmap of normalized gene expression of DEGs in iNSC19M7B^{OE} (light blue column annotation bar) as compared to control PK (orange column annotation bar). 643 DEGs are shown (log2FC |1| and $p$adj ≤ 0.05). Color range from orange to blue indicates high to low gene expression.

(D) Volcano plot of all DEGs in iNSC19M7B^{OE} as compared to iNSC19PK. x axis, log2FC; y axis, −log10($p$adj). Red dots: upregulated genes (log2FC > 0) in GIC19shM7B as compared to scr with $p$adj ≤ 0.05. Blue dots: downregulated genes (log2FC < 0) in GIC19shM7B as compared to scr with $p$adj ≤ 0.05. Gray: non-significant.

(E) Correlation scatterplot indicates genes of inverse log2FCs in the two contrasts. All genes shown in the plot are with $p$adj ≤ 0.05. The intersect is highlighted in purple: GICshM7B(up) (upregulated DEGs in GIC19shM7B vs. GIC19scr) and NSC M7B^{OE} (down) (downregulated DEGs in iNSC19M7B^{OE} vs. iNSC19PK).

(F) Deregulated C2 pathways in GICshM7B(up) and NSCM7B^{OE} (down) identified by enrichment analysis using a hypergeometric test. $p$adj < 0.05, DEGs are upregulated (log2FC > 0) in the GIC comparison (GIC19shM7B vs. GIC19scr) and downregulated (log2FC < 0) in the iNSC comparison (iNSCM7B^{OE} vs. iNSCPK). Bubbles are colored based on FDR values, and size is proportional to the number of genes included in each C2 term.

(G) Deregulated biological processes in GICshM7B(up) and iNSCM7B^{OE} (down). Bubbles are colored based on FDR values, and size is proportional to the number of genes included in each GO term.

(H) Venn diagram (left) of overlapping DMRs in the intersect; hypermethylated DMRs in GICshM7B vs. GICscr and hypomethylated DMRs in iNSCM7B^{OE} vs. NSCPK. For hypermethylated regions: maximum differences (maxdiff) between CpG probes > 0 and mean differences (meandiff) between CpG probes > 0, Fisher ≤ 0.05. For hypomethylated regions: maxdiff < 0 and meandiff < 0, Fisher ≤ 0.05. Right: Cytoscape visualization of GSEA; C2 pathway analysis of DMRs in the intersect GIChyper/iNSChypo. Bubbles are colored based on FDR values, and size is proportional to the number of genes included in each C2 term.

See also Figures S7 and S8.

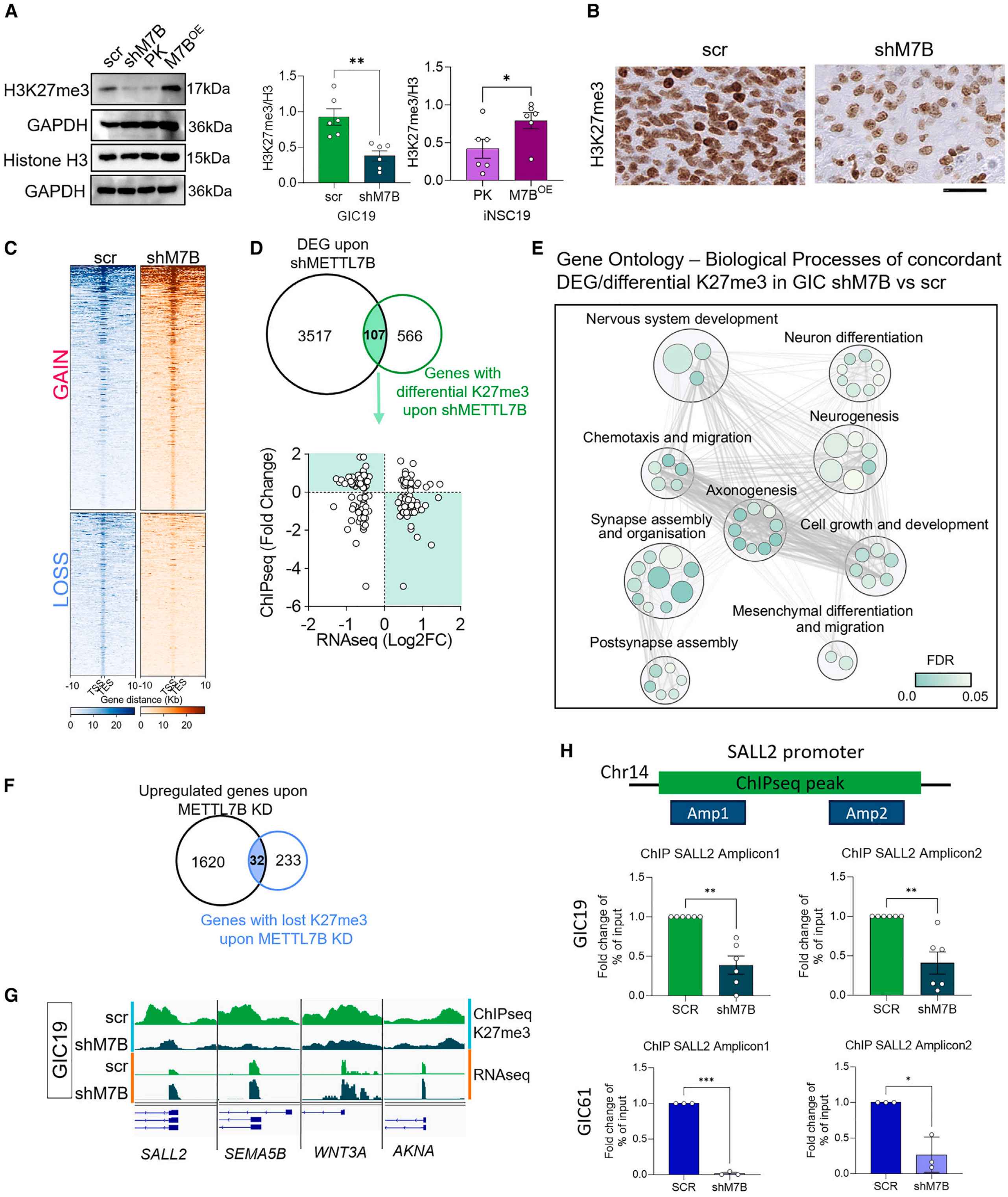

Figure 5. METTL7B epigenetically regulates genes involved in neuronal differentiation in GICs

(A) Western blot (left) and quantification (right) of tri-methylation of H3K27/total H3 histone (H3K27me3) in scr (light green) and upon METTL7B silencing (dark green) in GICs or in control (PK, light purple) and in METTL7B-overexpressing (M7B^{OE}, dark purple) iNSCs. GAPDH immunoreactivity was used to normalize protein loading. $N = 3$ independent experiments, unpaired two-tailed t test. All graphs report mean ± SEM of 6 blots. $^{**}p = 0.0022$ and $^{*}p = 0.0480$.

(B) Representative images of H3K27me3 IHC staining in GICscr- and GICshM7B-derived intracerebral xenografts. Scale bar: 50 μm.

(legend continued on next page)

Next, we took advantage of our syngeneic iNSC pairs edited to overexpress METTL7B to attempt to identify events directly regulated by METTL7B (i.e., those genes inversely regulated in the GICshM7B and iNSCM7B^{OE} contexts). The samples (n = 4 replicas from independent infections) clustered together, and there was a clear segregation of the experimental group in unsupervised clustering shown as a PCA plot (Figure S7F). A total of 5,586 significant DEGs were identified in iNSC19M7B^{OE} as compared to iNSC19PK, of which 2,899 were upregulated (Figure 4C), including *METTL7B*, confirming successful overexpression, and EZH2, and 2,687 were downregulated, which interestingly included *DNMT3A* and *DNMT3B* (Figure 4D). We primarily leveraged the analysis of the transcriptome of iNSCs to tease out genes and pathways directly regulated by METTL7B, in particular those predicted to be repressed by the gene. To this end, we identified 328 significant DEGs that exhibited both upregulation in the GIC19 comparison (GICshM7B vs. GICscr) and downregulation in the iNSC19 comparison (iNSCM7B^{OE} vs. iNSCPK) (Figure 4E). Using the C2 collection including CPs and chemical and genetic perturbation (CGP) pathways, gene sets associated with epigenetic mechanisms (e.g., enhancer marks, EZH2 targets, sumoylation of DNMTs, histone modifications), neurogenesis, stemness, and differentiation, as well as tumorigenesis and cell-cell adhesion, were identified as significantly enriched ($p$adj < 0.05) (Figure 4F). Further enrichment analysis identified significantly impacted GOBPs including cell-cell adhesion, neurogenesis, and neuron differentiation. Interestingly, DNA methylation and epigenetic regulation of gene expression, along with histone H3K4 methylation-, protein sumoylation-, and chromatin modification-related processes, were impacted (Figure 4G). The upregulation of *DNMT3A* and *DNMT3B* in the GIC context upon silencing of METTL7B as well as their downregulation in iNSCs overexpressing *METTL7B* were validated at the protein level when their expression level was sufficient to be detected (Figure S7G), raising the possibility that METTL7B influences DNA methylation by repressing key methyltransferases.

To explore this regulation, we investigated the DNA methylome of GICshM7B and of iNSCM7B^{OE} with the final goal to identify and characterize contrasting intersections. Firstly, each group (n = 4 replicas from independent infections) clustered closely, and there was clear separation of the groups in unsupervised clustering shown in PCA plots (Figure S8A). Significantly differentially methylated probes (DMPs) were identified for each comparison (Figure S8B). The predicted impact at the global level was not observed (Figure S8C); thus, any METTL7B-driven modulation of DNMT expression would have to be active in a region-specific fashion. To gain insight in the potential molecular impact of such a regulation, we identified common DMPs in GICshM7B vs. GICscr and iNSCM7B^{OE} vs. iNSCPK; in particular, we focused on hypermethylated DMPs in GICshM7B (GIChyper) and hypomethylated DMPs in iNSCM7B^{OE} (NSChypo), given the observed upregulation and downregulation of DNMTs in edited GICs and iNSCs, respectively. The probe-wise differential methylation analysis revealed 21,754 DMPs (Figure S8D), yielding 4,166 differentially methylated regions (DMRs), where each DMR comprised 6 or more CpG probes. DMRs were assigned to 3,379 genes, and enrichment analysis for C2 collection including CPs and CGPs on these differentially methylated genes (DMGs) revealed the enrichment of gene sets related to the JNK/MAPK8 pathway in ECM regulation and low-CpG-density promoters marked by H3K4me3 in neural progenitor cells as well as H3K27me3-marked gene sets (Figure 4H). Taking into account that METTL7B is a METTL protein itself, CP and CGP analysis of the 2,702 DMGs corresponding to the 3,454 regions hypomethylated upon knockdown (GIChypo) and hypermethylated upon overexpression (NSChyper) (Figure S8E) revealed the enrichment of gene sets marked by H3K4me3 and H3K27me3 and PRC2-complex-related pathways (Figure S8F).

We show that METTL7B modulates the expression of key methyltransferases, raising the possibility that it exerts its function at least in part by modulating DNA methylation in glioblastoma.

METTL7B epigenetically controls lineage specification in glioblastoma

To further clarify the observed link between METTL7B and mechanisms of epigenetic regulation via histone tail modification, we carried out a western blot for H3K27me3 and H3K4me3 on GICshM7B and in iNSCM7B^{OE}. We observed a striking reduction and increase of global H3K27me3 (Figure 5A),

(C) Heatmaps of H3K27me3 ChIP-seq peak enrichments in scr (blue) and shM7B (orange) GICs. Heatmaps are centered at transcription start site (TSS) covering ±10 Kb and ordered by H3K27me3 intensity. Signals were clustered based on the distribution of H3K27me3 surrounding the promoters and divided by genomic loci acquiring (GAIN) or losing (LOSS) the H3K27me3 mark upon METTL7B silencing.

(D) Venn diagram showing the integration of DEGs from RNA-seq data and genes presenting differential H3K27me3 peaks from ChIP-seq data (top). Volcano plot representing FCs of expression for DEGs (RNA-seq [log2FC]) and FC of H3K27me3 peak enrichment (ChIP-seq [FC]) upon METTL7B silencing in GICs (bottom). Highlighted genes with concordant gain/loss of H3K27me3 peaks and down-/upregulation.

(E) Bubble plot showing GO biological processes significantly enriched for the 63 concordant genes in GICs upon METTL7B silencing (highlighted in D, bottom) identified in ChIP-seq and RNA-seq integration. Bubbles are colored based on FDR values, and size is proportional to the number of genes included in each GO term.

(F) Venn diagram showing the overlap between genes upregulated and with loss of H3K27me3 in GICs upon METTL7B silencing.

(G) Genome browser view showing H3K27me3 ChIP-seq and RNA-seq peaks around SALL2, SEMA5B, WNT3A, and AKNA promoters in GIC scr (light green) and upon METTL7B silencing (shM7B, dark green).

(H) ChIP qPCR for the SALL2 promoter region where the H3K27me3 peak was identified through ChIP-seq (chromosome 14 [chr14]: 22,004,347–22,005,057). Two amplicons covering the region were analyzed in GIC19 (scr, dark green and shM7B, light green) and GIC61 (scr, dark blue and shM7B, light blue). N = 3 independent experiments. Graphs show FC of scr and shM7B normalized to the 1% of chromatin input for each sample. All graphs report mean ± SEM. Two-tailed paired t test. *p < 0.05, **p < 0.01, and ***p < 0.001.

See also Figure S9.

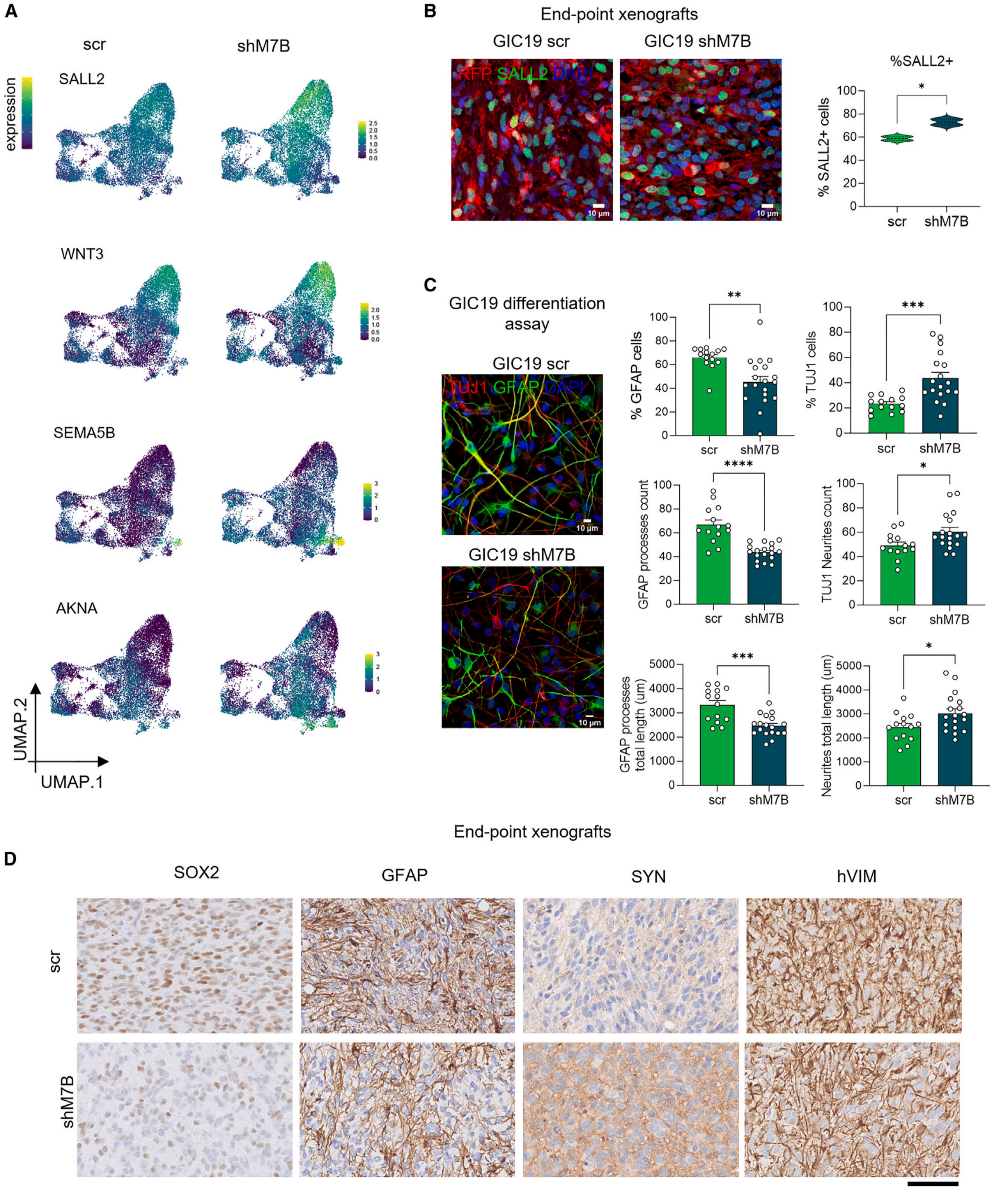

(legend on next page)

respectively, with an impact on H3K4me3 only observed in GICs (Figure S9A). To exclude that the phenotype was limited to *in vitro* culturing conditions and not reflecting the situation *in vivo*, immunohistochemistry for H3K27me3 was carried out on xenografts derived from GICscr and GICshM7B, which confirmed the reduction of global H3K27me3 upon METTL7B silencing (Figure 5B).

To understand the impact of the observed deregulation of H3K27me3 upon METTL7B modulation of expression, we performed a genome-wide chromatin immunoprecipitation sequencing (ChIP-seq) for the histone mark in GIC19 (Figure 5C) and iNSC19 (Figure S9B) and identified a redistribution of the mark, which mostly affected regulatory regions, in particular promoters and distal intergenic regions. Integration with RNA-seq datasets obtained from the same cells (Figure 5D, top) shows 107 genes, from which 63 genes showed concordant K27me3-mediated epigenetic regulation upon METTL7B modulation (i.e., loss of the K27me3 mark with increased expression and gain of the K27me3 mark with decreased expression) (Figure 5D, bottom), and GO analysis confirmed that 4/5 most significantly enriched BPs were related to neuronal differentiation (Figure 5E). Several regulators of neuronal differentiation were identified among the genes that lost the K27me3 mark in their promoter regions and exhibited increased gene expression in GICs upon METTL7B silencing, including SALL2, a key transcription factor regulating neuronal differentiation[36]; SEMA5B, which regulates axon growth during CNS development[37]; WNT3, a regulator of neurogenesis in the hippocampus[38]; and AKNA, a centrosome protein that regulates neurogenesis through microtubule organization[39] (Figures 5F and 5G). Similarly, GIC61 depleted for METTL7B showed significant loss of the H3K27me3 mark at the promoter region of the key neuronal transcription factor SALL2 (Figure 5H).

Of note, GO analysis of genes with concordant K27me3-mediated epigenetic regulation upon METTL7B overexpression in iNSCs (Figure S9C) shows significant enrichment for BPs related to neuronal differentiation too (Figure S9D) and for glial-differentiation-related BPs (Figure S9E). Interestingly, genes related to NSC biology and neurogenesis are found among those with the gained H3K27me3 mark and decreased expression in iNSCs overexpressing METTL7B (Figure S9F), including NEUROG2, CDON, and GAS1 (Figure S9G).

We next sought to validate the link between METTL7B expression and epigenetic regulation of neurogenesis via orthogonal datasets. Single-cell expression analysis of our PDXs confirmed that the inverse relationship between the *in vitro* ChIP-defined neurogenic regulators (SALL2, WNT3, SEMA5B, and AKNA) and METTL7B was maintained in an *in vivo* setting (Figure 6A). Intriguingly, SALL2 and WNT3 were predominantly expressed in AC and NSC/AC cells, raising the possibility that these cells could plastically transition to a more neuronal phenotype. By contrast, SEMA5B and AKNA were expressed most in the two populations that were enriched after METTL7B knockdown: OPC-like_2 and neuronal_2 cells, respectively. Importantly, immunostaining for SALL2 confirmed increased expression of the protein in xenografts derived from GIC19shM7B (Figure 6B). Expanding our analyses to glioma samples in TCGA, we observed significant negative correlations for SALL2, WNT3, and SEMA5B with METTL7B expression (Figure S10A).

At the functional level, *in vitro* differentiation of GIC19 shM7B led to a decreased number of cells expressing the astrocytic marker GFAP, as well as an increased number of cells expressing the neuronal differentiation marker TUJ1, at the end of the differentiation process (Figure 6C), and histological analysis of GICshM7B-derived xenografts confirmed the lower expression of the astrocytic markers SOX2 and GFAP as well as diffuse expression of the neuronal marker SYNAPTOPHYSIN in the METTL7B-deficient tumor cells (Figure 6D).

In conclusion, these data are in keeping with METT7LB regulating lineage specification in glioblastoma via epigenetic modulation of the expression of key players, such as SALL2.

DISCUSSION

We have identified *METTL7B* as an essential regulator of glioblastoma growth, with an impact on both tumor size and invasive properties in xenograft models. Single-cell transcriptomic analysis of these tumors and of COs derived from EPSCs overexpressing METTL7B identified an impact on lineage determination, with METTL7B regulating the NSC-to-AC trajectory in glioblastoma. Mechanistically, METTL7B downregulates the expression of key neuronal differentiation players, including SALL2, via post-translational modifications of histone marks.

Taking advantage of the SYNGN platform, we identified *METTL7B* as one of the genes more significantly differentially expressed between neoplastic and non-neoplastic stem cells in glioblastoma. Leveraging publicly available datasets,[16] we show that it is mainly expressed in AC-like glioblastoma cells. Intracerebral xenografts of *METTL7B*-depleted GICs induces the formation of smaller tumors with higher rates of apoptosis and a striking increase in brain invasion. Overall, the survival of mice bearing these GICs was prolonged by ~25% compared to controls, in keeping

Figure 6. Silencing of METTL7B in GICs drives cells toward a neuronal phenotype via METTL7B epigenetically regulated genes
(A) Expression intensity of concordant ChIP-/RNA-seq targets (SALL2, WNT3, SEMA5B, and AKNA) in our PDX scRNA-seq dataset.
(B) Representative images and quantification of immunostainings with SALL2 in xenografts derived from GIC19 intracerebrally injected with scr and shM7B. Immunostaining with anti-TurboRFP (GIC, in red) and SALL2 (green); nuclei counterstained with DAPI. Scale bar: 10 μm. $N = 2$ mice for each group, and $n = 3$ fields captured at 40×, where all cells were TurboRFP+. Graph reports double-positive cells for DAPI/SALL2 with mean ± SEM. Two-tailed unpaired t test. $^{*}p < 0.05$.
(C) Representative images and quantification of *in vitro* differentiation assay of GIC19scr and GIC19shM7B for 10 days. Immunostainings with GFAP (green) and TUJ1 (red). Nuclei were counterstained with DAPI. Scale bar: 10 μm. Graphs on the left show the quantification as a percentage (%) of GFAP (top graph) or TUJ1 (bottom graph), middle graphs show the quantification of GFAP processes (top) and TUJ1 neurites (bottom) as counts, and right graphs show the total length in μm of GFAP processes (top) and TUJ1 neurites (bottom). All results are based on $N = 2$ independent infections for each group, from scr: $n = 14$ and shM7B: $n = 18$. All graphs report mean ± SEM. Two-tailed unpaired t test. $^{*}p < 0.05$, $^{**}p < 0.01$, $^{***}p < 0.001$, and $^{****}p < 0.0001$.
(D) Representative images for SOX2, GFAP, SYN, and hVIM IHC staining in scr and shM7B GIC19-derived intracerebral xenografts. Scale bar: 100 μm. See also Figures S9 and S10.

with a previous report.[10] However, no impact on proliferation or EGR1 expression was found in our setting using primary patient-derived GICs as opposed to the established glioblastoma lines U87/U251.[10] Single-cell transcriptomic analysis revealed a decrease in NSC quiescence and an increase in OPC-like2/neuronal_2 clusters with the downregulation of the astrocytic/glial gene expression program and the upregulation of neuronal-specific genes and related regulatory pathways in the affected populations. Glioblastoma cell populations exhibiting neuronal phenotypes have been shown to be unconnected to other tumor cells but functionally connect to the surrounding healthy neurons and invade the surrounding brain tissue via *de novo* tumor microtubule (TM) formation.[19] AC/Mes tumor cell populations reside in the viable glioblastoma core in a well-connected tumor cell/AC network that has fewer TMs and less invasion potential. Our findings show higher expression of METTL7B in AC/Mes tumor cell subpopulation located in the tumor core. The increased proportion of cells with neuronal differentiation in xenografts upon silencing of *METTL7B* is in keeping with *METTL7B* regulating the lineage specification of GICs in favor of AC differentiation. Interestingly, we observed an increased invasiveness score in the shM7B-enriched OPC-like and neuronal clusters, which supports the *in vivo* observation of a more invasive nature of GICs lacking *METTL7B* and is in keeping with a more unconnected nature of neuronal differentiated tumor cells. Importantly, we show a cell-intrinsic mechanism of lineage and invasiveness regulation, which complements the current understanding of these glioblastoma properties as being mainly modulated by exposure of the neoplastic cells to the microenvironment.[40]

We observed increased apoptosis in the GICshM7B-derived xenografts. The paradoxical effect of increased invasion upon induction of apoptosis has been previously reported in glioblastoma, for example, upon treatment with the anti-angiogenic drug bevacizumab targeting VEGFA.[41–44] Here, the highly infiltrative phenotype after treatment is attributed to glioblastoma cells regulating ECM components, such as matrix metalloproteinases (MMPs),[45,46] potentially as an adaptive mechanism to evade apoptosis.[47] It is therefore conceivable that a similar mechanism contributes to the phenotype observed here, given the ECM pathway deregulation at the transcriptomic level observed.

Several members of the METTL family have been involved in the differentiation of stem cells, and in the retina, METTL14 directly regulates the expression of MAP2, which in turn binds to NEUROD1, resulting in the regulation of retinal pigment epithelial activity, which is hampered in retinitis pigmentosa.[48] We show that during brain development, *METTL7B* is expressed predominantly in outer radial glia cells and astroglial cells, a pattern recapitulated in COs at 3 and 6 months, respectively. COs derived from EPSC M7B^{OE} showed increased oRGs/AC cells and early neurons as well as depletion of more differentiated, particularly inhibitory, neurons, in keeping with the interpretation that *METTL7B* is a key regulator of lineage specification also during normal development. Interestingly, an increase in the proportion of neurons enriched for cell stress markers was also observed, a further indication of a compromised differentiation program upon the increased expression of METTL7B. As METTL7B^{OE} in COs did not induce tumor formation, it will be interesting to explore whether the gene can collaborate with additional (epi)genetic alterations to initiate tumorigenesis.

Transcriptome analysis of GICshM7B detected a significant increase in the expression of *DNMT1*, *DNMT3A*, and *DNMT3B*, and pathway analysis of DEGs confirmed DNA methylation and histone-modification-related pathways to be significantly affected. To assess whether this could be a direct mechanism of action of METTL7B, we analyzed the transcriptome of iNSCM7B^{OE} and confirmed decreased expression levels of these DNMTs as well as enrichment for pathways linked to epigenetic regulation of gene expression, including DNA methylation. We conclude that METTL7B is a repressor of DNMTs and therefore likely impacts DNA-methylation-mediated epigenetic regulation of gene expression. Importantly, however, we did not see an impact on global methylation in the neoplastic and normal settings, hence raising the possibility that fluctuation of the expression of the DNMTs has an impact only in specific genomic regions. Analysis of DNA methylation in these samples, with a particular focus on hypermethylated DMRs in GIC shM7B and hypomethylated DMRs in iNSCM7B^{OE}, revealed a striking enrichment for pathways involved in histone tail modifications, which regulate lineage commitment during differentiation and dedifferentiation. In fact, pathway analysis of these DMRs showed that the most significant gene sets affected included H3K27me3-marked genes involved in NPC differentiation.[49] Interestingly, an increase in EZH2, the methyltransferase of PRC2 responsible for the trimethylation of H3K27, was also identified. The predicted impact on PRC2 function, a well-characterized epigenetic regulator of neural cell differentiation,[50–52] could provide an interpretative frame for the astrocytic-to-neuronal switch observed in GICs upon METTL7B silencing.

METTLs, including METTL7B, have been involved in the deposition of the most abundant mRNA modifications: N6-methyladenosine (m6A), capable of regulating gene expression by affecting RNA metabolism, translation, and stability.[53,54] Methylation of m6A is known to be involved in crucial signal transduction pathways in cancer including PI3K/AKT, PTEN/mTOR, and Wnt signaling.[55–57] In glioblastoma, the upregulation of *METTL3* upon temozolomide treatment led to increased m6A methylation of histone modifiers, including EZH2 and HDAC2,[8] known to play a role in the development of resistance to the drug.[58] We find modulation of METTL3 expression in our GIC/iNSC setting, and it is therefore conceivable that a similar mechanism contributes to mediating METTL7B's impact on glioblastoma growth and invasion.

SALL2, also known as Spalt-like transcription factor 2, is a member of the SALL family of transcription factors involved in development and conserved through evolution. It has been associated with neuronal differentiation,[59] cell migration,[60] and cancer.[36] In the mouse, Sall2 protein is expressed by neuronal, but not astroglial, cells, and its expression increases during, and correlates with, neuronal differentiation.[59] Epigenetic regulation of SALL2 has been described; hypermethylation of its promoter was found to contribute to the acquisition of tamoxifen resistance in breast cancer.[61] In glioblastoma, SALL2 is a core transcription factor of the regulatory network essential for its growth.[62] We show here that it is epigenetically

regulated by METTL7B via PCR2-mediated modulation of H3K27me3 levels.

In conclusion, we show that METTL7B is an essential regulator of lineage specification in glioblastoma, raising the possibility that it could be exploited as a target for the development of therapeutic approaches specifically influencing plasticity-related glioblastoma features.

Limitations of the study

In this study, we have used one primary GIC line for the *in vivo* work and two lines for the *in vitro* validations. Although in larger human datasets, METTL7B expression correlates with the AC-like and the NPC/neuronal-like states positively and negatively, respectively, in keeping with our experimental data, it would be of interest to study additional xenograft models to further validate these transitions. In addition, we employ RNAvelocity to interrogate changes in lineage trajectories upon METTL7B knockdown. While RNAvelocity can provide valuable insight, fundamental assumptions of uni-directional development and transcriptional kinetics can break down in the context of cancer cells. For this reason, we have validated RNAvelocity-derived findings via orthogonal means. Finally, we propose here that METTL7B acts through the deregulation of *de novo* DNA methyltransferases and histone tail mark redistribution on defined genes; it will be of interest to further elucidate how METTL7B elicits this function.

STAR★METHODS

Detailed methods are provided in the online version of this paper and include the following:

- KEY RESOURCES TABLE
- RESOURCE AVAILABILITY
  - Lead contact
  - Materials availability
  - Data and code availability
- EXPERIMENTAL MODEL AND STUDY PARTICIPANT DETAILS
  - Patient-derived glioblastoma initiating cells (GIC)
  - Expanded potential stem cells (EPSC)
  - Induced neural stem cells (iNSC)
- METHOD DETAILS
  - Cerebral organoid generation
  - Plasmid design and construction
  - Lentiviral packaging
  - Animal procedures (Orthotopic xenografts)
  - RT-qPCR
  - Protein extraction and western blotting
  - Functional assays
  - Image analysis
  - DNA methylation and RNA sequencing
  - ScRNAseq
  - scRNAseq analysis
  - ChIPseq
  - ChIP seq analysis
  - ChIP-qPCR
- QUANTIFICATION AND STATISTICAL ANALYSIS

SUPPLEMENTAL INFORMATION

Supplemental information can be found online at https://doi.org/10.1016/j.celrep.2024.114309.

ACKNOWLEDGMENTS

This work is funded by Brain Tumor Research (Center of Excellence to S.M.), Cancer Research UK (C23985/A29199 to S.M.), Barts Charity (MGU0447 to S.M.), The Brain Tumour Charity (GN-000717 to C.V.), NIHR (UCLH BRC399/NS/RB/101410 and the Department of Health's BRC to S. Brandner), and the Cancer Research UK Centre of Excellence to Barts Cancer Centre (C16420/A18066 to E.M. and J.W.). We thank the staff of our animal facility for help caring for our mouse colony; Imran Uddin and Gordon Beattie, UCL Cancer Institute, for library preparation and preliminary analysis of scRNA-seq data; and Gary Warnes and Maeve McLaughlin for sharing their expertise. We acknowledge the use of data generated by TCGA Research Network: https://www.cancer.gov/tcga.

AUTHOR CONTRIBUTIONS

M.C. designed, performed, and analyzed CO experiments and contributed to cell culture experiments, scRNA-seq, RNA-seq, and DNA methylation, including analysis and interpretation of the results; J.N. analyzed and interpreted all scRNA-seq data; X.Z. contributed to cell culture experiments and carried out all animal experiments; E.M. analyzed ChIP-seq, RNA-seq, and DNA methylation data; S.L. contributed to all animal experiments and scRNA-seq experiments; G.R. carried out analysis of the SYNGN data; C.V. contributed to developing the SYNGN platform; J.W. supervised ChIP-seq, RNA-seq, and DNA methylation data analyses; Y.M.L. performed image analysis to quantify immunohistochemistry results; S. Brandner supervised image analysis; S.N. contributed expertise in the analysis of the SYNGN data; S. Badodi designed and carried out the ChIP-seq data generation and analysis; S.M. conceived the project, secured financial support, supervised the experiments, and wrote the paper with contributions from all authors, in particular M.C., J.N., and S. Badodi.

DECLARATION OF INTERESTS

The authors declare no competing interests.

Received: November 8, 2023
Revised: April 10, 2024
Accepted: May 16, 2024
Published: June 5, 2024

REFERENCES

1. Valor, L.M., and Hervás-Corpión, I. (2020). The Epigenetics of Glioma Stem Cells: A Brief Overview. Front. Oncol. *10*, 602378. https://doi.org/10.3389/fonc.2020.602378.
2. Safa, A.R., Saadatzadeh, M.R., Cohen-Gadol, A.A., Pollok, K.E., and Bijangi-Vishehsaraei, K. (2015). Glioblastoma stem cells (GSCs) epigenetic plasticity and interconversion between differentiated non-GSCs and GSCs. Genes Dis. *2*, 152–163. https://doi.org/10.1016/j.gendis.2015.02.001.
3. Uribe, D., Niechi, I., Rackov, G., Erices, J.I., San Martín, R., and Quezada, C. (2022). Adapt to Persist: Glioblastoma Microenvironment and Epigenetic Regulation on Cell Plasticity. Biology *11*, 313.
4. Raviram, R., Raman, A., Preissl, S., Ning, J., Wu, S., Koga, T., Zhang, K., Brennan, C.W., Zhu, C., Luebeck, J., et al. (2023). Integrated analysis of single-cell chromatin state and transcriptome identified common vulnerability despite glioblastoma heterogeneity. Proc. Natl. Acad. Sci. USA *120*, e2210991120. https://doi.org/10.1073/pnas.2210991120.
5. Tooley, J.G., Catlin, J.P., and Tooley, C.E.S. (2023). METTLing in Stem Cell and Cancer Biology. Stem Cell Rev. Rep. *19*, 76–91. https://doi.org/10.1007/s12015-022-10444-7.
6. Sun, Y., Shen, W., Hu, S., Lyu, Q., Wang, Q., Wei, T., Zhu, W., and Zhang, J. (2023). METTL3 promotes chemoresistance in small cell lung cancer by inducing mitophagy. J. Exp. Clin. Cancer Res. *42*, 65. https://doi.org/10.1186/s13046-023-02638-9.

7. Visvanathan, A., Patil, V., Arora, A., Hegde, A.S., Arivazhagan, A., Santosh, V., and Somasundaram, K. (2018). Essential role of METTL3-mediated m6A modification in glioma stem-like cells maintenance and radioresistance. Oncogene *37*, 522–533. https://doi.org/10.1038/onc.2017.351.

8. Li, F., Chen, S., Yu, J., Gao, Z., Sun, Z., Yi, Y., Long, T., Zhang, C., Li, Y., Pan, Y., et al. (2021). Interplay of m(6) A and histone modifications contributes to temozolomide resistance in glioblastoma. Clin. Transl. Med. *11*, e553. https://doi.org/10.1002/ctm2.553.

9. Liu, J., Yue, Y., Han, D., Wang, X., Fu, Y., Zhang, L., Jia, G., Yu, M., Lu, Z., Deng, X., et al. (2014). A METTL3–METTL14 complex mediates mammalian nuclear RNA N6-adenosine methylation. Nat. Chem. Biol. *10*, 93–95. https://doi.org/10.1038/nchembio.1432.

10. Xu, L., Peng, B., Wu, H., Zheng, Y., Yu, Q., and Fang, S. (2022). METTL7B contributes to the malignant progression of glioblastoma by inhibiting EGR1 expression. Metab. Brain Dis. *37*, 1133–1143.

11. Vinel, C., Rosser, G., Guglielmi, L., Constantinou, M., Pomella, N., Zhang, X., Boot, J.R., Jones, T.A., Millner, T.O., Dumas, A.A., et al. (2021). Comparative epigenetic analysis of tumour initiating cells and syngeneic EPSC-derived neural stem cells in glioblastoma. Nat. Commun. *12*, 6130. https://doi.org/10.1038/s41467-021-26297-6.

12. Yin, Y., Xie, C.M., Li, H., Tan, M., Chen, G., Schiff, R., Xiong, X., and Sun, Y. (2019). The FBXW2-MSX2-SOX2 axis regulates stem cell property and drug resistance of cancer cells. Proc. Natl. Acad. Sci. USA *116*, 20528–20538.

13. Bulstrode, H., Johnstone, E., Marques-Torrejon, M.A., Ferguson, K.M., Bressan, R.B., Blin, C., Grant, V., Gogolok, S., Gangoso, E., Gagrica, S., et al. (2017). Elevated FOXG1 and SOX2 in glioblastoma enforces neural stem cell identity through transcriptional control of cell cycle and epigenetic regulators. Genes Dev. *31*, 757–773. https://doi.org/10.1101/gad.293027.116.

14. Lattier, J.M., De, A., Chen, Z., Morales, J.E., Lang, F.F., Huse, J.T., and McCarty, J.H. (2020). Megalencephalic leukoencephalopathy with subcortical cysts 1 (MLC1) promotes glioblastoma cell invasion in the brain microenvironment. Oncogene *39*, 7253–7264. https://doi.org/10.1038/s41388-020-01503-9.

15. Wang, X., Prager, B.C., Wu, Q., Kim, L.J.Y., Gimple, R.C., Shi, Y., Yang, K., Morton, A.R., Zhou, W., Zhu, Z., et al. (2018). Reciprocal Signaling between Glioblastoma Stem Cells and Differentiated Tumor Cells Promotes Malignant Progression. Cell Stem Cell *22*, 514–528.e5. https://doi.org/10.1016/j.stem.2018.03.011.

16. Neftel, C., Laffy, J., Filbin, M.G., Hara, T., Shore, M.E., Rahme, G.J., Richman, A.R., Silverbush, D., Shaw, M.L., Hebert, C.M., et al. (2019). An Integrative Model of Cellular States, Plasticity, and Genetics for Glioblastoma. Cell *178*, 835–849.e21. https://doi.org/10.1016/j.cell.2019.06.024.

17. Jiang, Z., Yin, W., Zhu, H., Tan, J., Guo, Y., Xin, Z., Zhou, Q., Cao, Y., Wu, Z., Kuang, Y., et al. (2021). METTL7B is a novel prognostic biomarker of lower-grade glioma based on pan-cancer analysis. Cancer Cell Int. *21*, 383. https://doi.org/10.1186/s12935-021-02087-4.

18. Amodeo, V., A, D., Betts, J., Bartesaghi, S., Zhang, Y., Richard-Londt, A., Ellis, M., Roshani, R., Vouri, M., Galavotti, S., et al. (2017). A PML/Slit Axis Controls Physiological Cell Migration and Cancer Invasion in the CNS. Cell Rep. *20*, 411–426. https://doi.org/10.1016/j.celrep.2017.06.047.

19. Venkataramani, V., Yang, Y., Schubert, M.C., Reyhan, E., Tetzlaff, S.K., Wißmann, N., Botz, M., Soyka, S.J., Beretta, C.A., Pramatarov, R.L., et al. (2022). Glioblastoma hijacks neuronal mechanisms for brain invasion. Cell *185*, 2899–2917.e31. https://doi.org/10.1016/j.cell.2022.06.054.

20. Joseph, J.V., Magaut, C.R., Storevik, S., Geraldo, L.H., Mathivet, T., Latif, M.A., Rudewicz, J., Guyon, J., Gambaretti, M., Haukas, F., et al. (2022). TGF-β promotes microtube formation in glioblastoma through thrombospondin 1. Neuro Oncol. *24*, 541–553.

21. Wesolowska, A., Kwiatkowska, A., Slomnicki, L., Dembinski, M., Master, A., Sliwa, M., Franciszkiewicz, K., Chouaib, S., and Kaminska, B. (2008). Microglia-derived TGF-beta as an important regulator of glioblastoma invasion–an inhibition of TGF-beta-dependent effects by shRNA against human TGF-beta type II receptor. Oncogene *27*, 918–930.

22. Pietras, A., Katz, A.M., Ekström, E.J., Wee, B., Halliday, J.J., Pitter, K.L., Werbeck, J.L., Amankulor, N.M., Huse, J.T., and Holland, E.C. (2014). Osteopontin-CD44 signaling in the glioma perivascular niche enhances cancer stem cell phenotypes and promotes aggressive tumor growth. Cell Stem Cell *14*, 357–369.

23. Yu, C.-F., Chen, F.-H., Lu, M.-H., Hong, J.-H., and Chiang, C.-S. (2017). Dual roles of tumour cells-derived matrix metalloproteinase 2 on brain tumour growth and invasion. Br. J. Cancer *117*, 1828–1836. https://doi.org/10.1038/bjc.2017.362.

24. Venkatesh, H.S., Johung, T.B., Caretti, V., Noll, A., Tang, Y., Nagaraja, S., Gibson, E.M., Mount, C.W., Polepalli, J., Mitra, S.S., et al. (2015). Neuronal Activity Promotes Glioma Growth through Neuroligin-3 Secretion. Cell *161*, 803–816. https://doi.org/10.1016/j.cell.2015.04.012.

25. Wang, L., Jung, J., Babikir, H., Shamardani, K., Jain, S., Feng, X., Gupta, N., Rosi, S., Chang, S., Raleigh, D., et al. (2022). A single-cell atlas of glioblastoma evolution under therapy reveals cell-intrinsic and cell-extrinsic therapeutic targets. Nat. Cancer *3*, 1534–1552. https://doi.org/10.1038/s43018-022-00475-x.

26. Bergen, V., Soldatov, R.A., Kharchenko, P.V., and Theis, F.J. (2021). RNA velocity—current challenges and future perspectives. Mol. Syst. Biol. *17*, e10282. https://doi.org/10.15252/msb.202110282.

27. Zheng, S.C., Stein-O'Brien, G., Boukas, L., Goff, L.A., and Hansen, K.D. (2023). Pumping the brakes on RNA velocity by understanding and interpreting RNA velocity estimates. Genome Biol. *24*, 246. https://doi.org/10.1186/s13059-023-03065-x.

28. Wolf, F.A., Hamey, F.K., Plass, M., Solana, J., Dahlin, J.S., Göttgens, B., Rajewsky, N., Simon, L., and Theis, F.J. (2019). PAGA: graph abstraction reconciles clustering with trajectory inference through a topology preserving map of single cells. Genome Biol. *20*, 59. https://doi.org/10.1186/s13059-019-1663-x.

29. Dulken, B.W., Leeman, D.S., Boutet, S.C., Hebestreit, K., and Brunet, A. (2017). Single-Cell Transcriptomic Analysis Defines Heterogeneity and Transcriptional Dynamics in the Adult Neural Stem Cell Lineage. Cell Rep. *18*, 777–790. https://doi.org/10.1016/j.celrep.2016.12.060.

30. Kostović, I., Radoš, M., Kostović-Srzentić, M., and Krsnik, Ž. (2021). Fundamentals of the Development of Connectivity in the Human Fetal Brain in Late Gestation: From 24 Weeks Gestational Age to Term. J. Neuropathol. Exp. Neurol. *80*, 393–414. https://doi.org/10.1093/jnen/nlab024.

31. Liu, D.D., He, J.Q., Sinha, R., Eastman, A.E., Toland, A.M., Morri, M., Neff, N.F., Vogel, H., Uchida, N., and Weissman, I.L. (2023). Purification and characterization of human neural stem and progenitor cells. Cell *186*, 1179–1194.e15. https://doi.org/10.1016/j.cell.2023.02.017.

32. Uzquiano, A., Kedaigle, A.J., Pigoni, M., Paulsen, B., Adiconis, X., Kim, K., Faits, T., Nagaraja, S., Antón-Bolaños, N., Gerhardinger, C., et al. (2022). Proper acquisition of cell class identity in organoids allows definition of fate specification programs of the human cerebral cortex. Cell *185*, 3770–3788.e27. https://doi.org/10.1016/j.cell.2022.09.010.

33. Bhaduri, A., Andrews, M.G., Mancia Leon, W., Jung, D., Shin, D., Allen, D., Jung, D., Schmunk, G., Haeussler, M., Salma, J., et al. (2020). Cell stress in cortical organoids impairs molecular subtype specification. Nature *578*, 142–148. https://doi.org/10.1038/s41586-020-1962-0.

34. Torres-Peraza, J.F., Engel, T., Martín-Ibáñez, R., Sanz-Rodríguez, A., Fernández-Fernández, M.R., Esgleas, M., Canals, J.M., Henshall, D.C., and Lucas, J.J. (2013). Protective neuronal induction of ATF5 in endoplasmic reticulum stress induced by status epilepticus. Brain *136*, 1161–1176. https://doi.org/10.1093/brain/awt044.

35. Yang, J., Qi, M., Fei, X., Wang, X., and Wang, K. (2021). Long non-coding RNA XIST: a novel oncogene in multiple cancers. Mol. Med. *27*, 159. https://doi.org/10.1186/s10020-021-00421-0.

36. Hermosilla, V.E., Hepp, M.I., Escobar, D., Farkas, C., Riffo, E.N., Castro, A.F., and Pincheira, R. (2017). Developmental SALL2 transcription

factor: a new player in cancer. Carcinogenesis *38*, 680–690. https://doi.org/10.1093/carcin/bgx036.

37. de Wit, J., and Verhaagen, J. (2003). Role of semaphorins in the adult nervous system. Prog. Neurobiol. *71*, 249–267. https://doi.org/10.1016/j.pneurobio.2003.06.001.

38. Lie, D.C., Colamarino, S.A., Song, H.J., Désiré, L., Mira, H., Consiglio, A., Lein, E.S., Jessberger, S., Lansford, H., Dearie, A.R., and Gage, F.H. (2005). Wnt signalling regulates adult hippocampal neurogenesis. Nature *437*, 1370–1375. https://doi.org/10.1038/nature04108.

39. Camargo Ortega, G., Falk, S., Johansson, P.A., Peyre, E., Broix, L., Sahu, S.K., Hirst, W., Schlichthaerle, T., De Juan Romero, C., Draganova, K., et al. (2019). The centrosome protein AKNA regulates neurogenesis via microtubule organization. Nature *567*, 113–117. https://doi.org/10.1038/s41586-019-0962-4.

40. Varn, F.S., Johnson, K.C., Martinek, J., Huse, J.T., Nasrallah, M.P., Wesseling, P., Cooper, L.A.D., Malta, T.M., Wade, T.E., Sabedot, T.S., et al. (2022). Glioma progression is shaped by genetic evolution and microenvironment interactions. Cell *185*, 2184–2199.e16. https://doi.org/10.1016/j.cell.2022.04.038.

41. Norden, A.D., Young, G.S., Setayesh, K., Muzikansky, A., Klufas, R., Ross, G.L., Ciampa, A.S., Ebbeling, L.G., Levy, B., Drappatz, J., et al. (2008). Bevacizumab for recurrent malignant gliomas. Efficacy, toxicity, and patterns of recurrence *70*, 779–787. https://doi.org/10.1212/01.wnl.0000304121.57857.38.

42. Wang, N., Jain, R.K., and Batchelor, T.T. (2017). New Directions in Anti-Angiogenic Therapy for Glioblastoma. Neurotherapeutics *14*, 321–332. https://doi.org/10.1007/s13311-016-0510-y.

43. Thompson, E.M., Frenkel, E.P., and Neuwelt, E.A. (2011). The paradoxical effect of bevacizumab in the therapy of malignant gliomas. Neurology *76*, 87–93. https://doi.org/10.1212/WNL.0b013e318204a3af.

44. Kim, M.M., Umemura, Y., and Leung, D. (2018). Bevacizumab and Glioblastoma: Past, Present, and Future Directions. Cancer J. *24*, 180–186. https://doi.org/10.1097/ppo.0000000000000326.

45. Pullen, N., Pickford, A., Perry, M., Jaworski, D., Loveson, K., Arthur, D., Holliday, J., Van Meter, T., Peckham, R., Younas, W., et al. (2018). Current insights into Matrix metalloproteinases and glioma progression: transcending the degradation boundary. Metalloproteinases In Medicine *Volume*. Metalloproteinases Med. *5*, 13–30. https://doi.org/10.2147/MNM.S105123.

46. Lucio-Eterovic, A.K., Piao, Y., and de Groot, J.F. (2009). Mediators of Glioblastoma Resistance and Invasion during Antivascular Endothelial Growth Factor Therapy. Clin. Cancer Res. *15*, 4589–4599. https://doi.org/10.1158/1078-0432.Ccr-09-0575.

47. Mukai, M., Kusama, T., Hamanaka, Y., Koga, T., Endo, H., Tatsuta, M., and Inoue, M. (2005). Cross Talk between Apoptosis and Invasion Signaling in Cancer Cells through Caspase-3 Activation. Cancer Res. *65*, 9121–9125. https://doi.org/10.1158/0008-5472.Can-04-4344.

48. Yin, L., Ma, C., Hou, S., and Ma, X. (2022). Methyltransferase-like (METTL) 14-mediated N6-methyladenosine modification modulates retinal pigment epithelial (RPE) activity by regulating the methylation of microtubule-associated protein (MAP)2. Bioengineered *13*, 4773–4785. https://doi.org/10.1080/21655979.2022.2032968.

49. Mikkelsen, T.S., Ku, M., Jaffe, D.B., Issac, B., Lieberman, E., Giannoukos, G., Alvarez, P., Brockman, W., Kim, T.-K., Koche, R.P., et al. (2007). Genome-wide maps of chromatin state in pluripotent and lineage-committed cells. Nature *448*, 553–560. https://doi.org/10.1038/nature06008.

50. Corley, M., and Kroll, K.L. (2015). The roles and regulation of Polycomb complexes in neural development. Cell Tissue Res. *359*, 65–85. https://doi.org/10.1007/s00441-014-2011-9.

51. von Schimmelmann, M., Feinberg, P.A., Sullivan, J.M., Ku, S.M., Badimon, A., Duff, M.K., Wang, Z., Lachmann, A., Dewell, S., Ma'ayan, A., et al. (2016). Polycomb repressive complex 2 (PRC2) silences genes responsible for neurodegeneration. Nat. Neurosci. *19*, 1321–1330. https://doi.org/10.1038/nn.4360.

52. Schuettengruber, B., and Cavalli, G. (2009). Recruitment of Polycomb group complexes and their role in the dynamic regulation of cell fate choice. Development *136*, 3531–3542. https://doi.org/10.1242/dev.033902.

53. Wang, S., Lv, W., Li, T., Zhang, S., Wang, H., Li, X., Wang, L., Ma, D., Zang, Y., Shen, J., et al. (2022). Dynamic regulation and functions of mRNA m6A modification. Cancer Cell Int. *22*, 48. https://doi.org/10.1186/s12935-022-02452-x.

54. Huang, H., Weng, H., Sun, W., Qin, X., Shi, H., Wu, H., Zhao, B.S., Mesquita, A., Liu, C., Yuan, C.L., et al. (2018). Recognition of RNA N(6)-methyladenosine by IGF2BP proteins enhances mRNA stability and translation. Nat. Cell Biol. *20*, 285–295. https://doi.org/10.1038/s41556-018-0045-z.

55. An, Y., and Duan, H. (2022). The role of m6A RNA methylation in cancer metabolism. Mol. Cancer *21*, 14. https://doi.org/10.1186/s12943-022-01500-4.

56. Zhao, Q., Zhao, Y., Hu, W., Zhang, Y., Wu, X., Lu, J., Li, M., Li, W., Wu, W., Wang, J., et al. (2020). m(6)A RNA modification modulates PI3K/Akt/mTOR signal pathway in Gastrointestinal Cancer. Theranostics *10*, 9528–9543. https://doi.org/10.7150/thno.42971.

57. He, L., Li, H., Wu, A., Peng, Y., Shu, G., and Yin, G. (2019). Functions of N6-methyladenosine and its role in cancer. Mol. Cancer *18*, 176. https://doi.org/10.1186/s12943-019-1109-9.

58. Kitange, G.J., Mladek, A.C., Carlson, B.L., Schroeder, M.A., Pokorny, J.L., Cen, L., Decker, P.A., Wu, W., Lomberk, G.A., Gupta, S.K., et al. (2012). Inhibition of histone deacetylation potentiates the evolution of acquired temozolomide resistance linked to MGMT upregulation in glioblastoma xenografts. Clin. Cancer Res. *18*, 4070–4079. https://doi.org/10.1158/1078-0432.CCR-12-0560.

59. Pincheira, R., Baerwald, M., Dunbar, J.D., and Donner, D.B. (2009). Sall2 is a novel p75NTR-interacting protein that links NGF signalling to cell cycle progression and neurite outgrowth. EMBO J. *28*, 261–273. https://doi.org/10.1038/emboj.2008.274.

60. Riffo, E., Palma, M., Hepp, M.I., Benítez-Riquelme, D., Torres, V.A., Castro, A.F., and Pincheira, R. (2022). The Sall2 transcription factor promotes cell migration regulating focal adhesion turnover and integrin β1 expression. Front. Cell Dev. Biol. *10*, 1031262. https://doi.org/10.3389/fcell.2022.1031262.

61. Ye, L., Lin, C., Wang, X., Li, Q., Li, Y., Wang, M., Zhao, Z., Wu, X., Shi, D., Xiao, Y., et al. (2022). Epigenetic silencing of SALL2 confers tamoxifen resistance in breast cancer. EMBO Mol. Med. *14*, e15618. https://doi.org/10.15252/emmm.202115618.

62. Suvà, M.L., Rheinbay, E., Gillespie, S.M., Patel, A.P., Wakimoto, H., Rabkin, S.D., Riggi, N., Chi, A.S., Cahill, D.P., Nahed, B.V., et al. (2014). Reconstructing and Reprogramming the Tumor-Propagating Potential of Glioblastoma Stem-like Cells. Cell *157*, 580–594. https://doi.org/10.1016/j.cell.2014.02.030.

63. Stewart, S.A., Dykxhoorn, D.M., Palliser, D., Mizuno, H., Yu, E.Y., An, D.S., Sabatini, D.M., Chen, I.S.Y., Hahn, W.C., Sharp, P.A., et al. (2003). Lentivirus-delivered stable gene silencing by RNAi in primary cells. Rna *9*, 493–501. https://doi.org/10.1261/rna.2192803.

64. Dobin, A., and Gingeras, T.R. (2015). Mapping RNA-seq Reads with STAR. Curr. Protoc. Bioinformatics *51*, 11.14.1–11.14.19. https://doi.org/10.1002/0471250953.bi1114s51.

65. Li, B., and Dewey, C.N. (2011). RSEM: accurate transcript quantification from RNA-Seq data with or without a reference genome. BMC Bioinf. *12*, 323. https://doi.org/10.1186/1471-2105-12-323.

66. Love, M.I., Huber, W., and Anders, S. (2014). Moderated estimation of fold change and dispersion for RNA-seq data with DESeq2. Genome Biol. *15*, 550. https://doi.org/10.1186/s13059-014-0550-8.

67. Korotkevich, G., Sukhov, V., Budin, N., Shpak, B., Artyomov, M.N., and Sergushichev, A. (2021). Fast gene set enrichment analysis. Preprint at bioRxiv. https://doi.org/10.1101/060012.

68. Maksimovic, J., Phipson, B., and Oshlack, A. (2016). A cross-package Bioconductor workflow for analysing methylation array data [version 3; peer review: 4 approved]. F1000Research *5*, 1281. https://doi.org/10.12688/f1000research.8839.3.

69. Hao, Y., Hao, S., Andersen-Nissen, E., Mauck, W.M., Zheng, S., Butler, A., Lee, M.J., Wilk, A.J., Darby, C., Zager, M., et al. (2021). Integrated analysis of multimodal single-cell data. Cell *184*, 3573–3587.e29. https://doi.org/10.1016/j.cell.2021.04.048.

70. Korsunsky, I., Millard, N., Fan, J., Slowikowski, K., Zhang, F., Wei, K., Baglaenko, Y., Brenner, M., Loh, P.-r., and Raychaudhuri, S. (2019). Fast, sensitive and accurate integration of single-cell data with Harmony. Nat. Methods *16*, 1289–1296. https://doi.org/10.1038/s41592-019-0619-0.

71. Finak, G., McDavid, A., Yajima, M., Deng, J., Gersuk, V., Shalek, A.K., Slichter, C.K., Miller, H.W., McElrath, M.J., Prlic, M., et al. (2015). MAST: a flexible statistical framework for assessing transcriptional changes and characterizing heterogeneity in single-cell RNA sequencing data. Genome Biol. *16*, 278. https://doi.org/10.1186/s13059-015-0844-5.

72. Linderman, G.C., Zhao, J., Roulis, M., Bielecki, P., Flavell, R.A., Nadler, B., and Kluger, Y. (2022). Zero-preserving imputation of single-cell RNA-seq data. Nat. Commun. *13*, 192. https://doi.org/10.1038/s41467-021-27729-z.

73. La Manno, G., Soldatov, R., Zeisel, A., Braun, E., Hochgerner, H., Petukhov, V., Lidschreiber, K., Kastriti, M.E., Lönnerberg, P., Furlan, A., et al. (2018). RNA velocity of single cells. Nature *560*, 494–498. https://doi.org/10.1038/s41586-018-0414-6.

74. Bergen, V., Lange, M., Peidli, S., Wolf, F.A., and Theis, F.J. (2020). Generalizing RNA velocity to transient cell states through dynamical modeling. Nat. Biotechnol. *38*, 1408–1414. https://doi.org/10.1038/s41587-020-0591-3.

75. Venteicher, A.S., Tirosh, I., Hebert, C., Yizhak, K., Neftel, C., Filbin, M.G., Hovestadt, V., Escalante, L.E., Shaw, M.L., Rodman, C., et al. (2017). Decoupling genetics, lineages, and microenvironment in IDH-mutant gliomas by single-cell RNA-seq. Science *355*, eaai8478. https://doi.org/10.1126/science.aai8478.

76. Badodi, S., Pomella, N., Zhang, X., Rosser, G., Whittingham, J., Niklison-Chirou, M.V., Lim, Y.M., Brandner, S., Morrison, G., Pollard, S.M., et al. (2021). Inositol treatment inhibits medulloblastoma through suppression of epigenetic-driven metabolic adaptation. Nat. Commun. *12*, 2148. https://doi.org/10.1038/s41467-021-22379-7.

77. Badodi, S., Pomella, N., Lim, Y.M., Brandner, S., Morrison, G., Pollard, S.M., Zhang, X., Zabet, N.R., and Marino, S. (2022). Combination of BMI1 and MAPK/ERK inhibitors is effective in medulloblastoma. Neuro Oncol. *24*, 1273–1285. https://doi.org/10.1093/neuonc/noac052.

78. Langmead, B., and Salzberg, S.L. (2012). Fast gapped-read alignment with Bowtie 2. Nat. Methods *9*, 357–359. https://doi.org/10.1038/nmeth.1923.

79. Zhang, Y., Liu, T., Meyer, C.A., Eeckhoute, J., Johnson, D.S., Bernstein, B.E., Nusbaum, C., Myers, R.M., Brown, M., Li, W., and Liu, X.S. (2008). Model-based Analysis of ChIP-Seq (MACS). Genome Biol. *9*, R137. https://doi.org/10.1186/gb-2008-9-9-r137.

STAR★METHODS

KEY RESOURCES TABLE

REAGENT or RESOURCE	SOURCE	IDENTIFIER
Antibodies		
METTL7B (WB + IF)	Abcam	ab243710; RRID: AB_3101746
GAPDH (WB)	Sigma	G8795; RRID: AB_1078991
DNMT3A	Cell Signaling	3598S; RRID:AB_2277449
DNMT3B	Abcam	ab227883; RRID: AB_3101747
H3K27me3	Cell signaling	9733; RRID:AB_2616029
H3K4me3	Cell Signaling	9751S; RRID:AB_2616028
Histone H3	Abcam	ab1791; RRID:AB_30261
SALL2	Abcam	ab244275; RRID: AB_3101748
beta III Tubulin	Abcam	ab7751; RRID:AB_306045
GFAP	Agilent (Dako Omnis)	GA52461; RRID:AB_2811722
Chemicals, peptides, and recombinant proteins		
Y-27632 Dihydrochloride (ROCK inhibitor)	Biogems, PeproTech	Cat.: 1293823
murine EGF	PeproTech	Cat.: 315-09
human FGF	PeproTech	Cat.: AF-100-18B
Critical commercial assays		
CellTiter-Glo® Luminescent Cell Viability Assay	Promega	Cat.: G7571
CellTox™ Green Cytotoxicity Assay	Promega	Cat.: G8741
Deposited data		
RNA sequencing, DNA methylation, Single-cell RNA sequencing, ChIP sequencing	GEO	GSE243132
Experimental models: Cell lines		
Glioblastoma initiating cells (GIC): GIC19, GIC61, NH18-437, NH18-556, NH18760, NH18-1181, NH18-1218	Vinel et al.[11] and this paper	N/A
Induced Neural stem cells (iNSC): iNSC19, iNSC61	Vinel et al.[11]	N/A
Expanded potential stem cells (EPSC): EPSC19, EPSC61	Vinel et al.[11]	N/A
Oligonucleotides		
GAPDH (qPCR) Forward primer CTGAGGCTCCCACCTTTCTC	SIGMA	N/A
GAPDH (qPCR) Reverse primer TTATGGGAAAGCCAGTCCCC	SIGMA	N/A
METTL7B (qPCR) Forward primer CTCCAATATGAGCGGTTTG	SIGMA	N/A
METTL7B (qPCR) Reverse primer GAAAAAGAGCACACCTCC	SIGMA	N/A
SALL2 (ChIP-qPCR) Amplicon1 Forward primer ATCCCAAGTAACAAGCGGGG	SIGMA	custom
SALL2 (ChIP-qPCR) Amplicon1 Reverse primer CCCCTAAGTCCAAGCCTGTG	SIGMA	custom
SALL2 (ChIP-qPCR) Amplicon2 Forward primer AGCACAAACCTAAGCCCCTC	SIGMA	custom
SALL2 (ChIP-qPCR) Amplicon2 Reverse primer TTTGGGGTGTGTATGCGTGT	SIGMA	custom

(Continued on next page)

Continued

REAGENT or RESOURCE	SOURCE	IDENTIFIER
Recombinant DNA		
pKAM-BFP-Puro	Unpublished	Archibald Perkins Addgene plasmid # 101864; RRID:Addgene_101864
METTL7B (NM_152637) Human Tagged ORF Clone	origene	Cat.: RC203838
pGIPZ SMART-shMETTL7B-Turbo-RFP (Custom pGIPZ SMART lentiviral vector shMETTL7B with Turbo-GFP)	Horizon Discovery, Dharmacon	Custom
pCMV-VSV-G	Stewart et al.[63]	Bob Weinberg Addgene plasmid # 8454; RRID:Addgene_8454
pCMV-dR8.2 dvpr (pCMV-HIV-1 (gag/pol))	Stewart et al.[63]	Bob Weinberg Addgene plasmid # 8455; RRID:Addgene_8455
Software and algorithms		
ImageJ/FIJI	N/A	https://fiji.sc/
GraphPad Prism 10	GraphPad	RRID:SCR_000306
R studio	N/A	N/A
Cytoscape	N/A	v.3.10
Other		
NOD SCID CB17-Prkdcscid/J mice	Charles River UK Limited	N/A

RESOURCE AVAILABILITY

Lead contact

Further information and requests for resources and reagents should be directed to and will be fulfilled by the lead contact, Silvia Marino (s.marino@qmul.ac.uk).

Materials availability

- Plasmids generated in this study can be shared upon request.
- This study did not generate new unique reagents.

Data and code availability

- RNAseq, DNA methylation, single-cell RNA-seq and ChIPseq data have been deposited at GEO (GSE243132) and are publicly available as of the date of publication. Accession number is listed in the key resources table.
- This paper does not report original code.
- Any additional information required to reanalyze the data reported in this paper is available from the lead contact upon request.

EXPERIMENTAL MODEL AND STUDY PARTICIPANT DETAILS

Patient-derived glioblastoma initiating cells (GIC)

Following informed consent, tumor samples were obtained from patients undergoing surgery, and the use of human tissue samples was approved by the National Research Ethics Service (NRES), University College London Hospitals NRES Project ref. 08/0077 (S Brandner); Amendment 1 17/10/2014. GIC were isolated and cultured on laminin-coated plates in NeuroCult NS-A Proliferation kit medium (StemCell Technologies, 05751), Pen/Strep (Gibco), Heparin (2μg/ml StemCell Technologies), murine EGF (20 ng/ml, PeproTech, 315-09) and human FGF (10 ng/ml, PeproTech, AF-100-18B). When cell confluency reached 70–80%, cells were passaged using Accutase for dissociation, frozen in Stem Cell Banker (Ambsio ZENOAQ, 11890), and stored in liquid nitrogen.

Patient	Gender	Age
GIC19 (NH15-2101)	Female	65
GIC61 (NH16-2806)	Female	66
NH18-437	Female	60
NH18-556	Male	58

(*Continued on next page*)

Continued

Patient	Gender	Age
NH18-760	Male	57
NH18-1181	Female	66
NH18-1218	Male	36

Expanded potential stem cells (EPSC)

Dura-derived fibroblasts were reprogrammed into EPSCs and characterized as previously described by Vinel et al., 2021.[11] Feeder-dependent EPSC were transitioned to feeder free conditions and were cultured in geltrex-coated plates in mTeSR Plus complete medium (StemCell Technologies, #100–0276). EPSC colonies were passaged using 0.5mM EDTA in DPBS or ReLeSR (StemCell Technologies, # 100–0483) and dissociated into single cells using the Gentle cell dissociation reagent (StemCell Technologies, # 100–0485). EPSC were frozen in Stem Cell Banker and stored in liquid nitrogen.

Induced neural stem cells (iNSC)

EPSCs were differentiated into iNSC using the commercially available kit (Gibco Cat. #A1647801). iNSC were cultured in geltrex-coated plates in Neural expansion medium (0.5X Advanced DMEM/F-12 GIBCO 12634, 0.5X Neurobasal medium, GIBCO 211103-049, 1X Neurobasal Induction supplement, GIBCO A1647801, and 1X pen/strep Sigma, P4458). iNSC were passaged using Accutase for dissociation, cryopreserved in Synth-a-Freeze Medium (Gibco, A1371301) and stored in liquid nitrogen.

METHOD DETAILS

Cerebral organoid generation

Cerebral organoids were generated and cultured as per manufacturer's instructions using the STEMdiff Cerebral Organoid Kit (StemCell Technologies, #08570) with minor adaptations. Briefly, for embryoid bodies (EB) generation, feeder-free EPSC were dissociated into single cells and 60,000 cells were plated into each well of a 96-well round-bottom ultra-low attachment plate (Corning) in seeding media (EB formation media+50 μM ROCK Inhibitor) and media was replenished every 2 days. When EB diameter $\geq$300 μm (day7–8), EBs were transferred to a 24-well ultra-low attachment plate (Corning) in a neural induction medium for 3–4 days. EBs were then embedded in Matrigel hESC-Qualified Matrix (Corning, #354277) droplets and transferred into a six-well ultra-low attachment plate (Corning) in a neural expansion medium for 3 days. After 3 days, the expansion medium was removed from the wells and replaced with a maturation medium. The plate was transferred on an orbital shaker at 37°C at 100 rpm.

Plasmid design and construction

pKAM-BFP-Puro (addgene, #101864) and METTL7B (NM_152637) Human Tagged ORF Clone (origene, RC203838) were purchased and used for overexpression of METTL7B. Custom pGIPZ SMART lentiviral vector shMETTL7B with Turbo-RFP (Horizon Discovery, Dharmacon) was used for silencing. Second generation lentiviral packaging vectors pCMV-VSV-G and pCMV-HIV-1 were used for lentiviral particle packaging and production.

Lentiviral packaging

Lentiviruses expressing pGIPZ SMART-shMETTL7B-Turbo-RFP, pKAM-BFP-Puro or pKAM-METTL7B-BFP-Puro were produced by transfecting 4x10^6 HEK293T cells per 100mm dish with the lentiviral packaging vectors and plasmids of interest using the Lipofectamine 3000 reagents. Conditioned medium 24h and 52h post-transfection was harvested and centrifuged at 2000rpm for 5 min to remove any cell debris. The supernatant was then filtered through a 0.45μm filter (PVDF filter, Sartorius) and collected into a labeled sterilised glass bottle, with 5x Polyethylene glycol (PEG) added in each bottle to 1X, to precipitate the lentiviral particles at 4C overnight. Lentiviral particles were concentrated by centrifugation (30 min, 1500 × g, 4°C), resuspended in sterile PBS, aliquoted and stored at −80°C. Transducing units were then determined by plating 0.5 × 10^5 HEK293T cells into 12-well dishes and exposing them to serial dilutions 10^{-2} to 10^{-5} of the lentiviral particles. 96h post-transduction, the percentage of fluorescent positive cells was determined using the BD FACS Canto II. The titration in transducing units per mL (TU/ml) was calculated according to the following formula: (%positive cells/100) × number of transduced cells)/volume of virus (mL). All cells were infected with multiplicity of infection (MOI) 2.

Animal procedures (Orthotopic xenografts)

All procedures were performed in accordance with licenses held under the UK Animals (Home Office Guidelines: animals Scientific Procedures Act 1986, PPL 70/6452 and P78B6C064 Scientific Procedures) Act 1986. Eight to twelve-week-old NOD SCID CB17-Prkdcscid/J mice were anesthetised through intraperitoneal (IP) injection with a cocktail of anesthetic/analgesic drugs (Propofol: 60 mg/kg, Medetomidine: 1 mg/kg, Fentanyl: 200ug/kg). 5x105 cells were resuspended in 10 μL of PBS and slowly injected with a 26-gauge Hamilton syringe needle into the right cerebral hemisphere (stereotaxic coordinates from the bregma suture: 2 mm

posterior, 2 mm lateral, 4 mm deep, 10° angle). The scalps were sutured with 4-0 Coated Vicryl Suture (Ethicon) and mice were subcutaneously injected with an antidote solution (Atipamezol: 1 mg/kg, Butorphanol: 2 mg/kg; diluted in PBS) able to reverse the anesthetic effect. Mice were allowed to recover from the surgery on a heatmap until they were fully awake. Post-operative checks were performed twice a day for seven days, then once a day and body weight was monitored once a week until appearance of first symptoms. For survival experiments, mice were kept on tumor watch until symptoms developed. Mice were euthanized by neck dislocation and brains were harvested for histology and immunohistochemistry.

RT-qPCR

RNA was extracted from cell pellets using the RNeasy Mini or Micro Kit (Qiagen 74104/74004) following the manufacturer's instructions. RNA concentrations and quality were then assessed using the Nanodrop 1000. 500ng of RNA were retrotranscribed using SuperScriptIII (Invitrogen, 18080093). Samples were then diluted at 1:10 ratio in RNAse-free water and cDNA template was then mixed at a final volume of 12μL with the PowerUp SYBR Green master mix, including forward and reverse primers for the gene of interest or housekeeping gene. The samples were mixed in a 96-PCR plate and run on StepOnePlus Real-Time PCR System (ThermoFisher). For METTL7B, forward primer: CTCCAATATGAGCGGTTTG reverse primer: GAAAAAGAGCACACCTCC, for GAPDH, forward primer:

CTGAGGCTCCCACCTTTCTC, reverse primer: TTATGGGAAAGCCAGTCCCC.

All gene of interest (GOI) samples cycle threshold (Ct) were normalised to the housekeeping gene Ct; a threshold of exponential point on the standard curve was adjusted for each gene to calculate the ΔCt. Using the EXP formula in Microsoft Excel, where the constant e equals 2.71828182845904; the logarithmic base, the ΔΔCt was calculated. In order to calculate the RNA expression fold change values, the normalised ΔΔCt was normalised to the control samples (e.g., METTL7B overexpressing EPSC were normalised to the PK EPSC). GraphPad Prism 8 was then used to plot and visualise the results.

Protein extraction and western blotting

Protein extraction from GIC, NSC and EPSC samples was carried out using the Radioimmunoprecipitation (RIPA) Lysis Buffer System (SantaCruz, sc-24948A) to obtain cell lysates. Lysis buffer composed of RIPA buffer, protease inhibitor cocktail, sodium orthovanadate and phenylmethylsulfonyl fluoride (PMSF) protease inhibitor, was added to cell pellets. Samples were incubated on ice for 30 min with frequent vortexing every 5 min, then centrifuged at maximum speed at 4°C for 15 min. Cell lysate protein was quantified using the Pierce Bicinchoninic Acid (BCA) Protein Assay Kit (ThermoFisher, 23225), a colorimetric detection of the cuprous cation (Cu1+). Lysate samples were mixed with NuPAGE LDS sample buffer (Invitrogen, NP007) and incubated at 70°C for 10 min. Samples were then loaded onto the NuPAGE Bis-Tris 4-12% gels (Invitrogen, NP0322BOX) along with a Precision Plus Protein Dual Color Standards ladder (Biorad, 1610374). Gels were placed into the mini gel electrophoresis tank (Invitrogen, A25977) filled with NuPAGE MOPS SDS Running Buffer (Invitrogen, NP0001) and ran for 30 min at 100V, then 25 min at 150V and finally 15 min at 200V. Proteins were transferred onto a Nitrucellulose or Polyvinylidene fluoride (PVDF) membrane (Life Technology, LC2005) using the mini blot module. 1x NuPAGE transfer buffer composed of NuPAGE transfer buffer (Invitrogen, NP0006), methanol and deionised water. Sponge pads, Whatman filter papers, gel and membrane were soaked in transfer buffer before assembled into the blot module. The cassette was then placed into the tank submerged in transfer buffer and ran at 15V for 2 h. Membranes were then transfer into a 5% milk blocking buffer consisted of milk powder (ChemCruz SC-2325) in tris-buffered saline tween 20 (TBST) for 1 h at room temperature on low-speed shaker. Primary antibodies were added overnight in 5% milk buffer and incubated overnight at 4°C on a shaker. Membranes were then washed with TBST for 3–5 times for 5 min and secondary antibodies were added at room temperature on low-speed shaker. Membranes were washed thoroughly in TBST and the ECL prime (GE Healthcare, RPN2106) was used to develop the membranes for 5 min at room temperature in the dark. The Chemidoc MP imaging system (BioRad) was used for image acquisition.

Functional assays

CellTiter-Glo luminescent Cell Viability Assay to assess proliferation

The Promega kit: CellTiter-Glo Cell Viability Assay (Promega Cat. #G7571) was used to assess the proliferation rate of GIC. The CellTiter Glo substrate was mixed with the CellTiter Glo buffer. Cells were plated at a density of 6000 cells per well of a 96 well plate in 100μL of media. On day 1, 3 and 5, 100μL of the CellTiter Glo reagent was added into each well. The reagent lyses the cells, resulting in release of cellular adenosine triphosphate (ATP) that is then catalyzed by the UltraGlo recombinant luciferase and produces a luminescent signal. The Biotek Synergy-HT plate reader was used with the following settings: Costar 96 white opaque, Luminescence Endpoint, Integration Time: 1.0 (SS.s), Filter Set 1, Emission: Hole, Optics: Top, Gain: 100, Read Speed: Normal, Read Height: 1 mm. The signal is an indicator of ATP presence and hence cell viability, and therefore reading of day 3 and day 5 were normalised to day 1 to create a proliferation rate curve. Proliferation was then assessed as area under the curve (AUC).

CellTox Green Cytotoxicity Assay to assess cell death

The Promega kit: CellTox Green Cytotoxicity Assay (Promega Cat. #G8741) was used to assess the cell death of GIC. Cells were plated at a density of 6000 cells per well of a 96 well plate in 100μL of media. 72hours post seeding, 4μL of lysis solution was added to toxicity control wells and incubated for 30 min. 20μL of concentrated CellTox Green reagent was then added to all wells and the plate was incubated at room temperature for 15 min, shielded from direct light. The assay uses a cyanide dye to measure

CellPress
OPEN ACCESS

the membrane integrity by binding dead cell's DNA leading to enhanced fluorescent signal produced by the dye. The fluorescent signal generated by binding on dead cells' DNA is proportional to cytotoxicity. The Biotek Synergy-HT plate reader was used with the following settings: Costar 96 white opaque, Fluorescence Endpoint, Filter Set 1, Excitation: 485/20, Emission: 528/20, Optics: Bottom, Gain: 35, Read Speed: Normal. Cell death was assessed as compared to the positive lysed toxicity controls.

Soft agar colony formation assay

7500 cerebral organoid cells were mixed with 0.3% noble agarose (BD #214220) in complete cerebral organoid maturation medium, plated on top of a solid field layer of 0.6% noble agarose in cerebral organoid maturation medium, per well of a 6-well plate. Media was replenished every 3 days. After 3–4 weeks, the colonies were fixed and dyed with Crystal Violet (0.005% in 4% formaldehyde, Sigma, C0775), washed with PBS, and imaged.

Differentiation assay

0.8 × 105 GIC scr and shM7B were plated on geltrex-coated cover slips in 24 well plates in NSC differentiation media (NeuroCult NS-A Differentiation Kit (Human), Stemcell Technologies # 05752). Cells were cultured for 10 days and media was replenished every 48 h.

Invasion/migration assays

Transwell inserts with 8.0 μm pores (Sarstedt Cat. #89.3932.800) were placed into wells of a 24 well plate and coated with 100 μL of geltrex for 1–3 h. 100,000 cells were seeded on the top chamber in 200 μL of media, and 700 μL of media was added to the bottom well. 24 h post-seeding, cells on the membrane on the top chamber of the transwell were removed using a cotton bud dampened with DPBS, and the bottom of the transwell was fixed in 4% PFA at room temperature for 15 min. The membrane was the washed with DPBS, cut and mounted onto a microscopy slide with mounting media including DAPI (Invitrogen P36931). Transwell membranes were then analyzed on the Zeiss 880 Confocal microscope and four-five representative images of nuclei using the 40× objective were captured for each membrane. The experiment was repeated three times (different passages of cells lines), two-three technical replicate membranes were used. The number of migrated cells across the membrane was counted as the number of nuclei in each image captured using the ImageJ/FIJI software.

Scratch-wound healing assay

96 well plates were coated with laminin for 3 h - overnight. 60,000 cells were seeded in each well and allowed to reach 100% confluency. To inhibit proliferation, cells were treated for 1 h at 37°C with fresh media containing 20 μg/mL Mitomycin-C. The bottom of the wells were vertically scratched with a 10 μL tip and media was replaced with fresh media containing laminin. Following the scratch (day 0), pictures were taken at marked location using the 10× objective on the EVOS, and the same location was captured after 24 h (day 1) incubation. The surface wound area (scratch area) was calculated using ImageJ/FIJI, normalized to day 0 scratch area and compared to the control (fold change). The experiment was repeated 2–3 times (different passages of cell lines) with 3–5 technical replicas each time.

Image analysis

Immunofluorescence and immunohistochemistry

iNSC and GIC plated on geltrex or laminin-coated cover slips respectively, and differentiated iNSC and GIC were fixed in 4% PFA and washed in PBS. Cells were permeabilised with 1% Donkey or Goat serum, 0.1% Triton X- in 0.1% BSA in PBS for 1 h at room temperature, and stained for METTL7B (Santa Cruz, sc-398626), beta III Tubulin (Abcam, ab7751), GFAP (Dako, 20025480) overnight at 4C. After three washes with PBS and 1 h incubation with secondary antibodies diluted in PBS, cells were washed again with PBS, counterstained with DAPI and mounted onto SUPERFROST slides (Thermoscientific, J1800AMNZ). Confocal microscope (Zeiss 710) was used to acquire the images at 40× magnification and images were analyzed on ImageJ/FIJI and FIJI/NeuronJ plugin.

Xenografts

Slides stained for hVIM, Ki67 and cleaved Caspase 3 (Roche Discovery XT platform) were scanned at 40× objective magnification using a Hamamatsu NanoZoomer S360 slide scanner. QuPath was used to analyze these whole-slide images (WSI) 55. Color deconvolution parameters were established based on the highest and lowest intensity of haematoxylin and DAB staining for each stain. Tissue sections were then segmented and annotated using a pixel classifier to distinguish the sections from glass. Subsequently, "Tissue" annotations were eroded by 35 μm to exclude most of the extracerebral tumors.

For hVIM images, hVIM detection objects were created using a pixel classifier on the DAB deconvolved channel. "Tumor core" annotations were created by eroding hVIM detection objects by 10 μm to smooth out tumor processes, followed by a 10 μm dilation to rsetore the original border size. "Tumor core" annotations with an area <10,000 μm2 were removed to exclude small islands. "Gross tumor" annotations were created by dilating hVIM detection objects by 75 μm to fill gaps between close objects, and then eroded by 75 μm to return to the original border distance. "Gross tumor" annotations without a "Tumor core" annotation were excluded. The resulting areas for "Tumor core" and "Gross tumor" annotations were measured and the following calculated: (a) % hVIM area = total area of hVIM/total area of Tissue annotations and (b) invasiveness index = gross tumor area/tumor core area.

For Ki67 and cleaved Caspase 3 images, DAB-positive cells were detected using StarDist 56 from the DAB deconvolved channel. An object classifier was used to identify Ki67-or cleaved Caspase 3-positive cells for their respective images. The positive cell density within tumor regions was calculated by: Cells per mm2 vimentin = number of positive cells/total hVIM area.

DNA methylation and RNA sequencing

DNA and RNA from iNSC and GIC were extracted using the RNA/DNA/Protein Purification Plus Kit (Norgen, #47700) following the manufacturer's protocol. DNA and RNA were then quantified with the Nanodrop.

RNA-Sequencing was performed at Novogene with paired-end, 150bp sequence reads. Library preparation (mRNA, poly-A enrichment) and sequencing was performed on a NovaSeq6000 platform, generating on average 48 million reads per sample. Reads were quality trimmed using trimgalore v0.6.5 and aligned to reference genome GRCh37 (hg19) using STAR v2.7.9a.[64] Counting of reads was performed with RSEM v1.3.1[65] using the ENSEMBL annotation GRCh37.87. Only genes that achieved at least one read count per million reads (CPM) in at least 25% of samples (i.e., 6) were kept. This led to 14,649 filtered genes in GIC19 and 14746 in NSC19 cell lines. Differential expression analysis was performed in DESeq2[66] using the Wald test. Differentially expressed genes were thresholded with padj $\leq$ 0.05. Gene set enrichment analysis was performed using R package fgsea[67] and the ranked DESeq2 Wald statistic of all sufficiently detected genes. The analysis was performed for Canonical Pathways (c2.cp.v7.4.symbols.gmt) and Gene Ontology Biological Processes (c5.go.bp.v7.4.symbols.gmt). Enrichment analysis of overlapping genes of interest (over-representation analysis) was performed with R packages msigdbr and clusterProfiler, on the C2 and C5 human MSigDB collections, using a hypergeometric test. Heatmaps were plotted with R package ComplexHeatmap; PCA was performed and plotted with R packages factoextra, FactoMiner, ggsci and tidyverse; Volcano plots with R package EnhancedVolcano; scatterplots with R packages gplots and ggplot2.

DNA methylation was assessed on the Illumina Infinium MethylationEpic kit at the UCL Genomics, Institute of Child Health. Illumina Human Methylation EPIC microarray data were analyzed using a cross-package Bioconductor workflow in R, employing the methylation specific packages minfi, IlluminaHumanMethylationEPICanno.ilm10b4.hg19, missMethyl and DMRcate as well as limma as described in.[68] Briefly, raw data were imported in R using minfi and quality control was performed by assessing mean detection *p*-values of all CpGs in every sample to identify and remove potential failed samples. Normalisation was performed using minfi's preprocessQuantile method. Poor performing probes that failed in one or more samples were filtered out prior to differential methylation analysis retaining only good quality probes with detection *p*-value <0.01, these were in total 835,820 probes. Probe-wise differential methylation analysis was performed using limma and probes with Benjamini and Hochberg adjusted *p*-value <0.05 were considered statistically significant. Differential methylation analysis of regions was performed using the dmrcate function of R package DMRcate based on the annotated with genomics position M-values matrix and limma.

ScRNAseq

Mouse xenografts preparation

Selected mice for scRNAseq were culled by neck dislocation and the brains were dissected and placed into HBSS (Gibco, 14175095) in petri dishes. Brains were cut into small pieces with a surgical blade and then dissociated into single cell suspension using the Brain Tumor Dissociation Kit (P) (Miltenyi Biotec,130-095-942) following the manufacturer's protocol. Cells were centrifuged at 300g x for 10 min, followed by cell debris removal using the Debris Removal Solution (Miltenyi Biotec, 130-109-398) as per protocol instructions. Mouse cells were removed using the Mouse Cell Depletion Kit (Miltenyi Biotec, 130-104-694) as per manufacturer instructions with minor adaptations. Cells were centrifuged and manually counted. 30000 cells were isolated, centrifuged again at 300 g × 5 min and re-suspended in 0.04% BSA in PBS at a concentration of 1000 cells/μL.

Cerebral organoid preparation

COs for single-cell RNA sequencing were processed with the same Neural Tissue Dissociation Kit (P) using the gentle MACS dissociator. 3–5 pooled day70 COs derived from gene-edited EPSC were harvested and washed with D-PBS without calcium and magnesium. The organoids were then transferred into a C-Tube containing the pre-heated enzyme mix 1 followed by the gentleMACS Program m_brain_01, 37°C incubations under slow continuous rotation, gentleMACS Program m_brain_02, addition of enzyme mix 2 and gentleMACS Program m_brain_03. Single-cell suspension was passed through a 70μm strainer and centrifuged at 300g x for 10 min. CO cells were then resuspended in 0.04% BSA in DPBS, and viability and cell number were assessed with the Countess II FL Automated Cell Counter using trypan blue. 100,000 cells were aliquoted into a new tube in 100μL to achieve a cell density of 1000 cells/μL as required by the 10X Genomics Single-cell RNA sequencing protocol, followed by a final count to ensure the correct number of cells.

Library preparation

Cells were then submitted for processing and library preparation at the Single Cell Genomics Facility (UCL, Cancer Institute). The Chromium Next GEM Single Cell 3′ Kit v3.1 (10X Genomics, 1000268) was used along with the Chromium Next GEM Chip G Single Cell Kit (10X Genomics, 1000120).

Cells were processed according to 10X Genomics User Guide (Chromium Next GEM Single Cell 3' Reagent Kits v3.1 (Dual Index)). Cells were resuspended with RT Master Mix and loaded onto Chip G along with the barcoded gel beads and partitioning oil in order to generate the gel bead-in-emulsion (GEM) on the Chromium Controller. Once complete, the GEMS were loaded onto a thermocycler to complete the RT inside each GEM to produce cDNA, which contain Illumina R1 primer sequence primer sequence, Unique Molecular Identifier (UMI) and the 10X Barcode. Silane Dynabeads were used to clean up the pooled barcoded cDNA. The cDNA was then amplified, and a quick QC was conducted to look at the fragment sizes and concentrations of the samples. 10ul of the amplified cDNA was taken forward for the library construction which involves 3 steps of fragmentation (to obtain fragments around 450bp), ligation (to add the Illumina p5 and p7 primers) and Indexing (to add unique sample index to each sample). These steps were followed

by an SPRI Select bead purification. Once Indexing was complete the libraries were pooled and submitted for sequencing on Illumina platform. 10,000 cells of each sample were loaded on the 10X Genomics apparatus with the aim of labeling and capturing 7,000 cells. Pooled samples libraries were sequenced with lane sequencing on Novaseq 6000 S4 (Novogene).

scRNAseq analysis

The Cell Ranger 2.0.1 pipeline was used to align reads to the GRCh38 human reference genome and produce count matrices for downstream preprocessing and analysis using the Seurat v4.0 R package.[69] For PDX samples, alignment was also performed against the Mm10 reference genome, and the number of aligning reads to each reference genome was calculated, with cells logFC _Hs_Mm < 1 excluded as contaminants. For quality filtration, minimum features thresholds of 400 and 750 genes, and percentage mitochondrial thresholds of 10% and 7.5% were used for PDX and CO datasets respectively. Expression values were library size corrected to 10,000 reads per cell and log1p transformed, with Principal component analysis (PCA) performed on the scaled data for the top 2000 variable genes. Batch correction was performed on principal components using Harmony.[70] Uniform Manifold Approximation Projection embeddings, Nearest Neighbors and cell clusters were then calculated in harmony-corrected PCA space using the default settings of Seurat's RunUMAP(), FindNeighbors(), and FindClusters() functions. Cluster marker genes were calculated using a Wilcoxon Rank-Sum test, and differential expression analysis across experimental conditions was performed using MAST.[71] For expression plots of concordant ChIP/RNAseq targets (Figure 6A), and invasion associated genes (Figure S3E) we used ALRA-imputed data.[72] For RNAvelocity analysis, velocyto[73] was used to calculate spliced and unspliced transcriptomes, before subsequent analysis using scVelo[74] and PAGA,[28] on Seurat-defined cell types and UMAP embeddings, using default parameters. For reference signature scoring, average gene module expression was calculated for each single cell, subtracted by the aggregated expression of a random control set of features selected from the same average expression bins as the query genes.[75] G/S and G2M gene modules are included in the Seurat v4.0 package; GSC cell state signatures were taken from and visualized as in Neftel et al.,[16] and invasiveness signatures were taken from.[19] For GSC state signature scoring in the reference cohort[25] we analyzed samples with at least 500 tumor cells as above, before separating samples into METTL7B-high (n = 27) or low METTL7B-low (n = 27) groups based on median expression and comparing cell state composition between groups.

For CO data scRNAseq cluster gene expression was compared against cell type signatures from two single cell resources of organoid development.[32,33] In each case we obtained both the raw count matrices and author-defined cluster meta data and used them to perform a Wilcoxon Rank-Sum test to obtain the list of cluster marker genes for their cell types. Then, for each dataset and marker gene, cluster specificity scores were computed (mean normalized counts per cluster/total mean normalized counts) – with gene signature specificity scores compared across studies by Pearson correlation. For each pair of datasets, we restricted analyses to the intersection of their full marker gene lists.

ChIPseq

H3K27me3 ChIP was performed using ChIP-IT High Sensitivity kit (active Motif) as described previously.[76,77] Briefly, cells were fixed with the formaldehyde-based fixing solution for 15 min at room temperature and lysed with provided lysis solution supplemented with proteases inhibitors. Next, nuclei pellets were lysed and chromatin sonicated with Bioruptor Plus sonication device (Diagenode) to obtain fragments within the recommended 200–1200 bp range. 5 μg of sheared chromatin was then incubated with 4 μg of antibody against H3K27me3 (Diagenode) overnight at 4°C with rotation. Following incubation with Protein G agarose beads, bound chromatin was washed, eluted and purified following the manufacturer's protocols. Validation by qPCR-ChIP on target genes was done before proceeding to sequencing. ChIPed DNA was end-repaired, A-tailed and adapter-ligated before size selection and amplification. The obtained libraries were QC'ed and multiplexed before 150-bp paired-end sequencing on NovaSeq6000 (Illumina) at Oxford Genomics with an average sequencing depth of 47 million reads per sample.

ChIP seq analysis

The quality of ChIPseq samples was assessed via FASTQC after merging the fastq files of the two lanes of sequencing. Adapter trimming was performed using Trimgalore v0.6.5 and alignment to hg19 reference genome was performed using Bowtie2 v2.4.5[78] with default parameters. Exploratory tools as deeptools, plotCorrelation, plotPCA and plotFingerprints were used to further asses sample characteristics and to address potential outliers. samtools v1.9 was used to obtain bam from sam files that were then sorted by coordinate and indexed. Bams were filtered to include only uniquely mapping reads (i.e., unmapped reads and multimapping were removed) using sambamba. Peak calling was performed with MACS2,[79] the shifting model was disabled to make different datasets comparable, the BAM mode was used (-f BAM and also -g 'hs') and and the "–broad" option was enabled for the analysis of the histone mark H3K27me3. (–broad and –broad-cutoff 0.05) and (300bp fragment size –extsize 300). Further quality assessment of peak calling was performed using R package ChIPQC. Differential peak analysis was performed with R package DiffBind. ChIP peak annotation was performed with R package ChIPseeker, Interval set operations and overlaps were identified with R package GenomicRanges. Pathway enrichment analysis of gene lists of interest (over-representation analysis) was performed as described in the RNAsequencing section of the methods.

ChIP-qPCR

For ChIP-qPCR, cells were cultured in laminin-coated 15cm dishes, and once 70–80% confluency was reached, chromatin was extracted from the cells as described in the ChIPseq section. 7-18 μg of sheared chromatin was then incubated with 4 μg of antibody against H3K27me3 (Diagenode), and the bound chromatin was washed and eluted. ChIP-qPCR on positive (EVX1) and negative (GAPDH) target genes was performed and compared to 1% Input (sheared chromatin) and mock (no antibody) samples. SALL2 primers targeting the ChIP peak promoter region were designed using primer BLAST.

QUANTIFICATION AND STATISTICAL ANALYSIS

All statistical analysis and generation of graphs was performed using GraphPad Prism 10. Data are presented as mean ± SEM. NS indicates non-significant with $p > 0.05$. $p < 0.05$ was considered statistically significant, with p values $p \leq 0.05$, $p \leq 0.01$, $p \leq 0.001$ and $p \leq 0.0001$ represented with *, **, ***, **** respectively. Differences between the two groups were analyzed by using the student's unpaired or paired t test. One-way or 2-way analysis of variance (ANOVA) was used to investigate more than two groups. Further information of the statistical analysis of specific datasets is indicated in the figure legends.

Cell Reports, Volume *43*

Supplemental information

Lineage specification in glioblastoma

is regulated by METTL7B

Myrianni Constantinou, James Nicholson, Xinyu Zhang, Eleni Maniati, Sara Lucchini, Gabriel Rosser, Claire Vinel, Jun Wang, Yau Mun Lim, Sebastian Brandner, Sven Nelander, Sara Badodi, and Silvia Marino

Constantinou et al. Supplementary Figures and Figure Legends

Constantinou et al. Figure S1

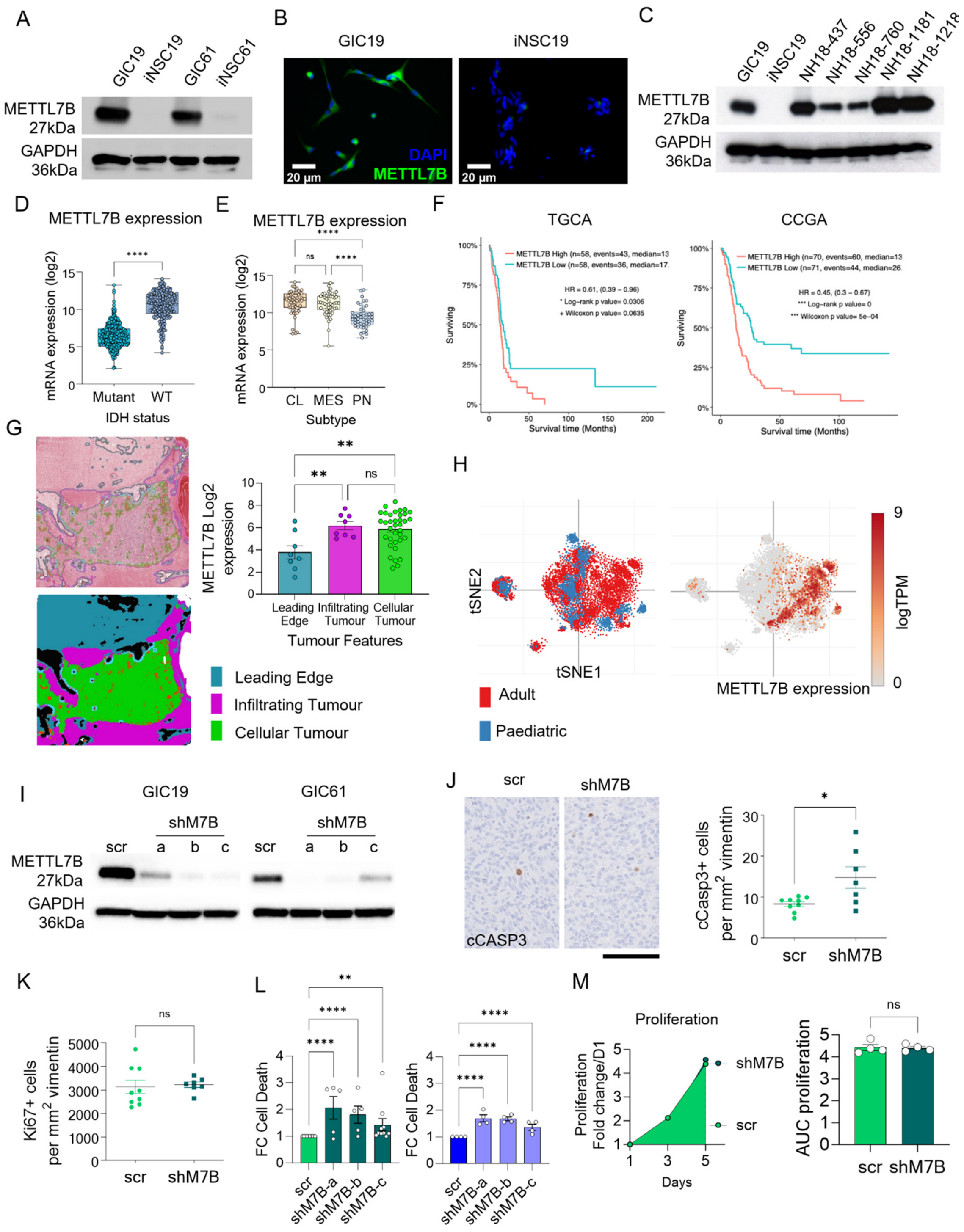

Figure S1 METTL7B is overexpressed in IDH-wildtype glioblastoma. Related to Figure 1

A. Western blot of METTL7B in syngeneic pairs GIC19/iNSC19, GIC61/iNSC61. GAPDH used as a loading control.

B. Immunofluorescence staining of METTL7B (green) in iNSC19 and GIC19 cells. Nuclei were counterstained with DAPI. Scale bar=20µm.

C. Western blot of METTL7B in 5 primary GIC lines from an independent cohort compared to iNSC19 and GIC19. GAPDH used as a loading control.

D. Analysis of *METTL7B* expression in IDH- mutant and wild-type (WT) gliomas. N= 429 mutant and 233 WT IDH independent glioma samples from TCGA. Two-tailed unpaired t-test, ****p<0.0001. Whiskers showing min and max points.

E. Analysis of *METTL7B* expression in glioblastoma subtypes, n= 56 Classical, 51 Mesenchymal and 46 Proneural independent glioma samples from TCGA. 2-way ANOVA, p-value of the pairwise comparisons is shown. ****p-value<0.0001, ns, not significant. Whiskers showing min and max points.

F. Survival curves of glioma, IDH-wildtype patients from TCGA cohort (M7B high n=58, M7B low n=58) and CGGA cohort (M7B high n=70, M7B low n=71).

G. Spatial expression of METTL7B in GBM bulk samples, analysed on Ivy (GAP). Left: example of histological anatomic structure identification in a sub-block. Right: Expression of *METTL7B* in RNAseq data from anatomic structures shown as log2 normalized gene expression. Leading Edge defined as the border of the tumour, where ratio of tumour to normal cells is 1-3 / 100. Infiltrating tumour defined as the intermediate zone between leading edge and cellular tumour, where ratio of tumour to normal cells is 10-20 /100. Cellular tumour defined as tumour core, where tumour to normal cells is 100-500 / 1. Graphs show mean ± SEM. 2-way ANOVA, p-value of the pairwise comparisons is shown. **p-value<0.001.

H. *METTL7B* expression in scRNAseq data of paediatric (blue) and adult (red) glioblastoma samples. (Clusters defined in Neftel et al, Cell 2019 [1], data accessed and visualized on Single Cell Portal of The Broad Institute of MIT and Harvard. METTL7B expression in tSNE, with logTPM expression ranging from light orange to dark.

I. Western blot analysis of protein expression levels of METTL7B upon silencing in GIC19 and GIC61 (shM7B) using three different shRNA (-a, -b, -c), as compared to the scramble control (scr).

J. IHC staining for cleaved-Caspase 3 (cCasp3) in brains of mice orthotopically injected with GICscr and shM7B and quantification of cCasp3-positive cells per mm^2 of tumour. Light green: scr n=9 animals, Dark green: shM7B n=7 animals. Welch's t test, *p-value=0.049. Scale bar = 100 µm.

K. Proliferation assessment of GIC in PDX model as quantitative assessment of Ki67+ cells in the xenografts, Welch's t test, p-value=0.77. Light green: scr n=9 animals. Dark green: shM7B n=7 animals

L. Cell death in GIC19 and GIC61 upon METTL7B silencing (GIC19 shM7B, dark green, GIC61 shM7B, light blue) as compared to control (GIC19 scr, light green, GIC61 scr, dark blue).For GIC19 (scr: N=10, n=3-5, shM7B-a: N=5, n=3-5, shM7B-b: N=5, n=3-5, shM7B-c: N=10, n=3-5). For GIC61 (N=4 for all groups, and n=4-10 technical replicas). Two-way ANOVA, error bars as SEM, p-values: **p-value<0.01, ***p-value<0.001, ****p-value<0.0001.

M. Proliferation rate of GIC19 upon METTL7B silencing (shM7B-c, dark green) as compared to control (scr, light green). Proliferation difference is shown as area under the curve over 5 days. (N=4 biological replicates., Two-tailed unpaired t-test, error bars as SEM, p-values: ***p-value<0.001, ns not significant.

Constantinou et al. Figure S2

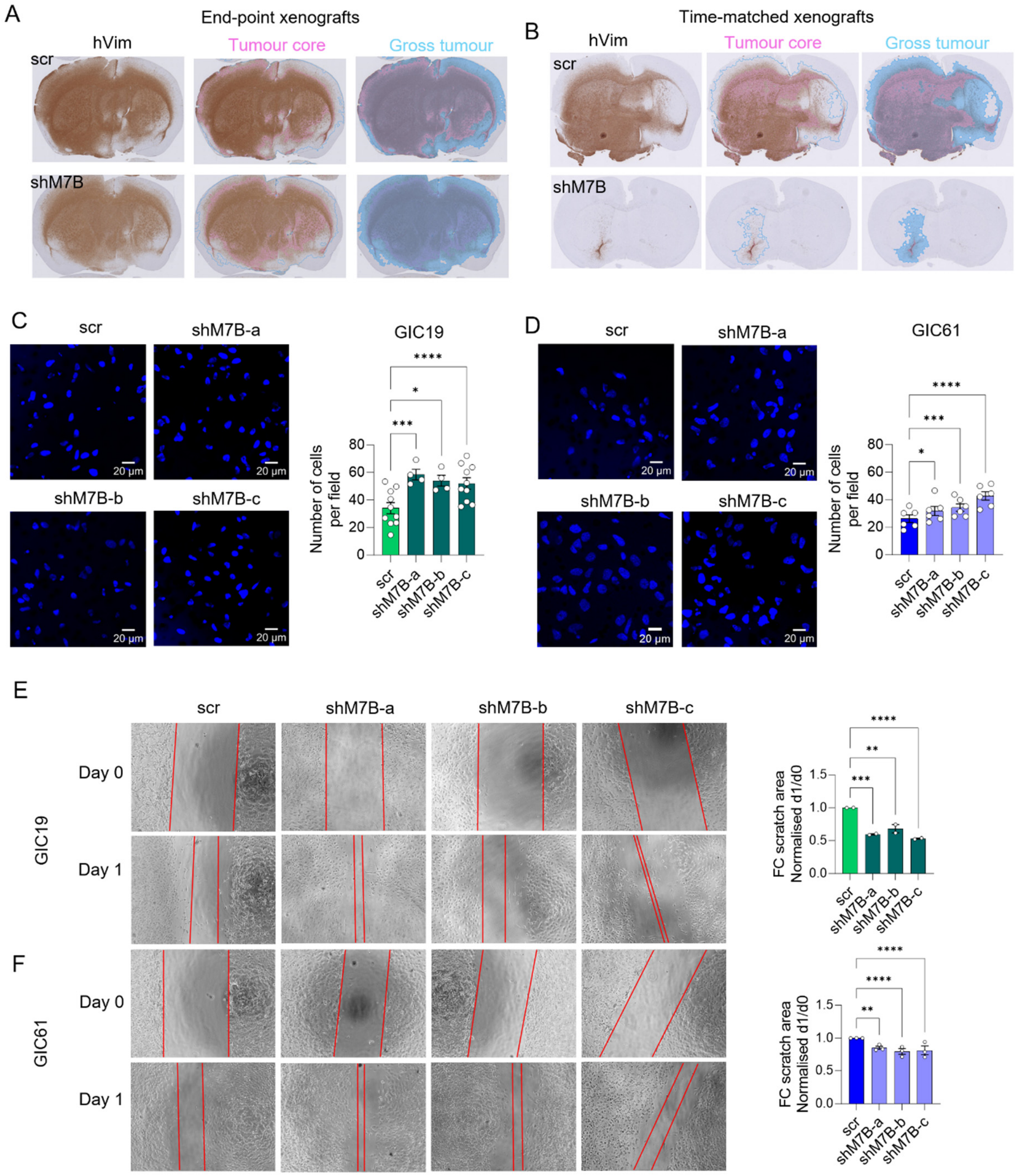

Figure S2 METTL7B silencing in GIC leads to increased invasion. Related to Figure 1

A. Vimentin stained sections of xenografts at end-point overlayed with the masks used to quantify the invasiveness index. The invasiveness index is defined as gross tumour area (blue) /tumour core area (pink).

B. Vimentin stained sections of time-matched xenografts overlayed with the masks used to quantify the invasiveness index. The invasiveness index is defined as gross tumour area (blue) /tumour core area (pink).

C. Transwell assay to assess invasion of GIC19 upon METTL7B silencing with three different shRNA (GIC19 shM7B, dark green) as compared to control (GIC19 scr, light green). Representative images of scr and shM7B captured at 40x magnification (scale bar at 20 µm). Graph shows the number of cells that migrated through the transwelll membrane per image field captured. N=3 independent experiments, 2-3 membranes per replica and 5-7 images per membrane captured at 40x. Two-Way ANOVA, error bars as SEM, p-values: *p-value<0.05, **p-value<0.01, ***p-value<0.001, ****p-value<0.0001.

D. Transwell assay to assess invasion of GIC61 upon METTL7B silencing with three different shRNA (GIC61 shM7B, light blue) as compared to control (GIC61 scr, dark blue). Representative images of scr and shM7B captured at 40x (scale bar at 20 µm). Graph shows the number of cells that migrated through the transwelll membrane per image field captured. N=3 independent experiments, 2-3 membranes per replica and 5-7 images per membrane captured at 40x magnification. Two-way ANOVA, error bars as SEM, p-values: *p-value<0.05, **p-value<0.01, ***p-value<0.001, ****p-value<0.0001.

E. Scratch-wound healing assay to assess migration of GIC19 upon METTL7B silencing with three different shRNA (GIC19 shM7B, dark green) as compared to control (GIC19 scr, light green). Representative images brightfield images of scr and shM7B captured at 10x. Red lines indicate the scratch area on day 0 and day 1. Graph shows fold change of scr and shM7B of the scratch area on day 1 normalised to day 0. N=2 independent experiments, 2-5 technical replicas (wells) per condition. Two-way ANOVA p-values, error bars as SEM, p-values: *p-value<0.05, **p-value<0.01, ***p-value<0.001, ****p-value<0.0001.

F. Scratch-wound healing assay to assess migration of GIC61 upon METTL7B silencing with three different shRNA (GIC61 shM7B, light blue) as compared to control (GIC61 scr, dark blue). Representative images brightfield images of scr and shM7B captured at 10x. Red lines indicate the scratch area on day 0 and day 1. Graph reports fold

change of scr and shM7B of the scratch area on day 1 normalised to day 0. N=3 independent experiments, 2-5 technical replicas (wells) per condition. Two-way ANOVA, error bars as SEM, p-values: *p-value<0.05, **p-value<0.01, ***p-value<0.001, ****p-value<0.0001.

Constantinou et al. Figure S3

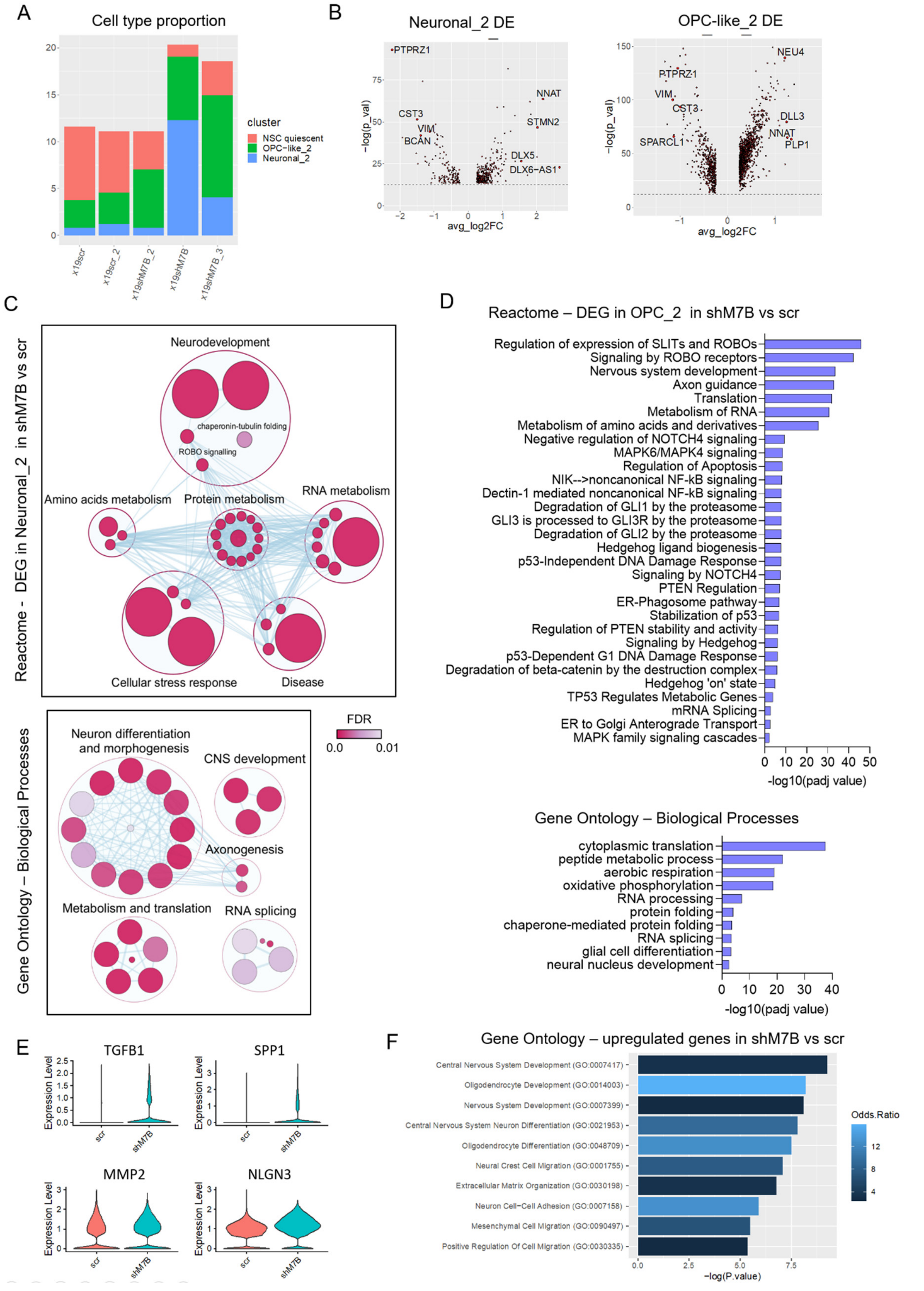

Figure S3 Single-cell RNAseq analysis and DE genes in GICshM7B derived xenografts and controls. Related to Figure 2

A. Cell type proportions of most affected clusters in scr and shM7B Cell cycle classification of NSC/NPC clusters.
B. Volcano plots showing DE genes in GICshM7B as compared to scr in Neuronal 2 (top) and OPC-like 2 (bottom) clusters. X axis: average log2 fold change (avg_log2FC), Y axis: -log(p_val). Upregulated genes in GICshM7B: avg_log2FC>0, Downregulated genes in GICshM7B: avg_log2FC<0.
C. Gene set enrichment analysis of Neuronal_2 DE genes in GICshM7B as compared to control scr, of significantly affected Reactome pathways (left) and gene ontology biological processes (GOBP, right) visualised on Cytoscape. Bubbles are coloured based on FDR values and size is proportional to the number of genes included in each Reactome or GO term.
D. Gene set enrichment analysis of OPC-like_2 DE genes in GICshM7B as compared to control scr, of the 30 most significantly affected Reactome pathways (top) and top 10 GOBP (bottom) visualised on Cytoscape. Bars showing –log10(padj).
E. Violin plots show expression levels of invasion/migration markers in the two groups (scr and shM7B).
F. GO analysis of upregulated genes in shM7B as compared to scr.

Constantinou et al. Figure S4

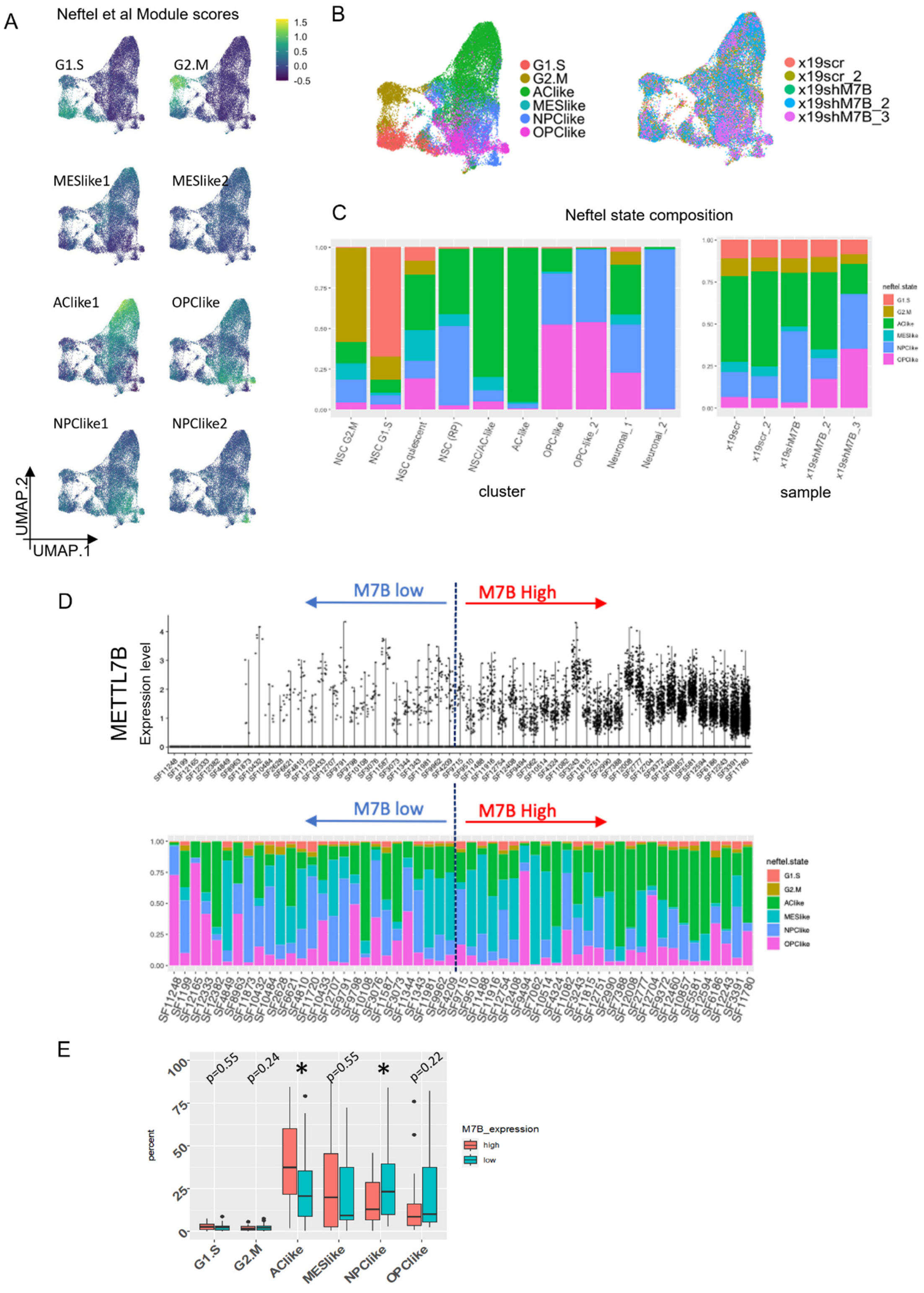

Figure S4 High METTL7B expression is associated with the AC-like state in scRNAseq datasets. Related to Figure 2

A. UMAPs showing single cell expression enrichment of patient-derived GIC cell state signature genes.

B. UMAP showing Neftel cell state identity (assigned by highest signature score) and plot of cell state proportions in each sample.

C. Bar plots showing composition of signature scoring assigned Neftel cell state identities in cell types (left) and samples (right) .

D. Violin plot showing High (n=27) and low (n=27) METTL7B expression groups in a cohort of patient GBM scRNAseq samples (Wang et al., 2022) (top panel), as compared to their signature scoring assigned Neftel cell state compositions (bottom panel)

E. Bar graphs showing the cell state composition in samples with high and low METTL7B expression.

Constantinou et al. Figure S5

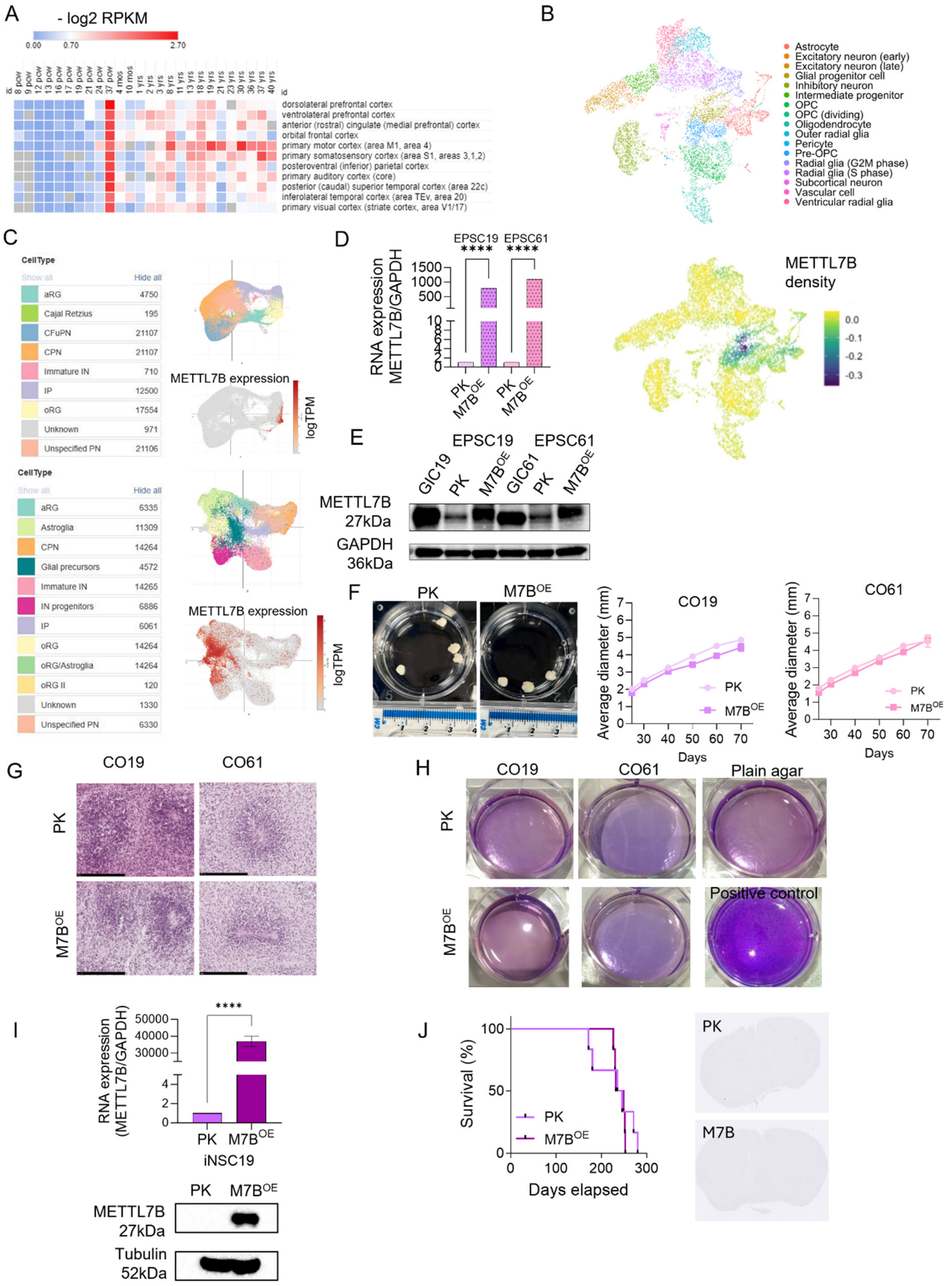

Figure S5 METTL7B is expressed during neurodevelopment. Related to Figure 3

A. Expression of METTL7B in the developing and adult human cortex, from 8pcw to 40 years, extracted from the BrainSpan Atlas. RNA seq data analysed for different developing regions of the developing cortex. Expression is shown as log2 RPKM (reads per kilobase per million) values of n=3 for each time point.
B. Single-cell RNAseq on 17-19GW fetal brain samples. Left shows UMAP of cells colored by cell type, right shows expression density plot of METTL7B.
C. Single-cell RNAseq on 3-month (top) and 6-month (bottom) COs. N=3-4 COs per timepoint. Left: Clusters annotations of UMAP (right top). Right bottom: METTL7B expression counts in the clusters, shown in a 2D plot as logTPM expression ranging from light orange to dark.
D. METTL7B relative RNA expression levels in METTL7B overexpressing EPSC19 and EPSC61 as compared to control (PK) (n=3, biological replicas, n=3 technical replicas). Ordinary one-way ANOVA, ****P<0.0001.
E. METTL7B protein expression levels in METTL7B overexpressing EPSC19 and EPSC61 as compared to control (PK), and syngeneic GIC pairs.
F. Cerebral organoids at day 70 of CO19 PK and M7B. The average diameter in mm was measured from day 25 to 70 of CO19 and CO61 PK and M7B. Quantification of average diameter of 3-4 COs per condition for each line.
G. H&E of day 70 COs PK and M7B in both lines. Scale bar is 250μm.
H. Soft agar assay at 3 weeks post seeding of dissociated PK and M7B COs from both lines. Plain agar: negative control, NSC overexpressing NFIX: positive control. Crystal violet stain at end point.
I. Left: METTL7B RNA expression in iNSC19 upon overexpression of METTL7B ($M7B^{OE}$, dark purple), as compared to the control (PK, light purple). N=4: independent lentiviral transductions. Right: Protein expression levels of METTL7B in iNSC19 upon overexpression of METTL7B, as compared to the PK control. Tubulin was used as a loading control.
J. Survival curves of mice injected with control PK (light purple) and iNSC19 $M7B^{OE}$ (dark purple). (n=6 for each group). Left: hVim immunostaining of mouse brains injected with iNSC19 PK and $M7B^{OE}$

Constantinou et al. Figure S6

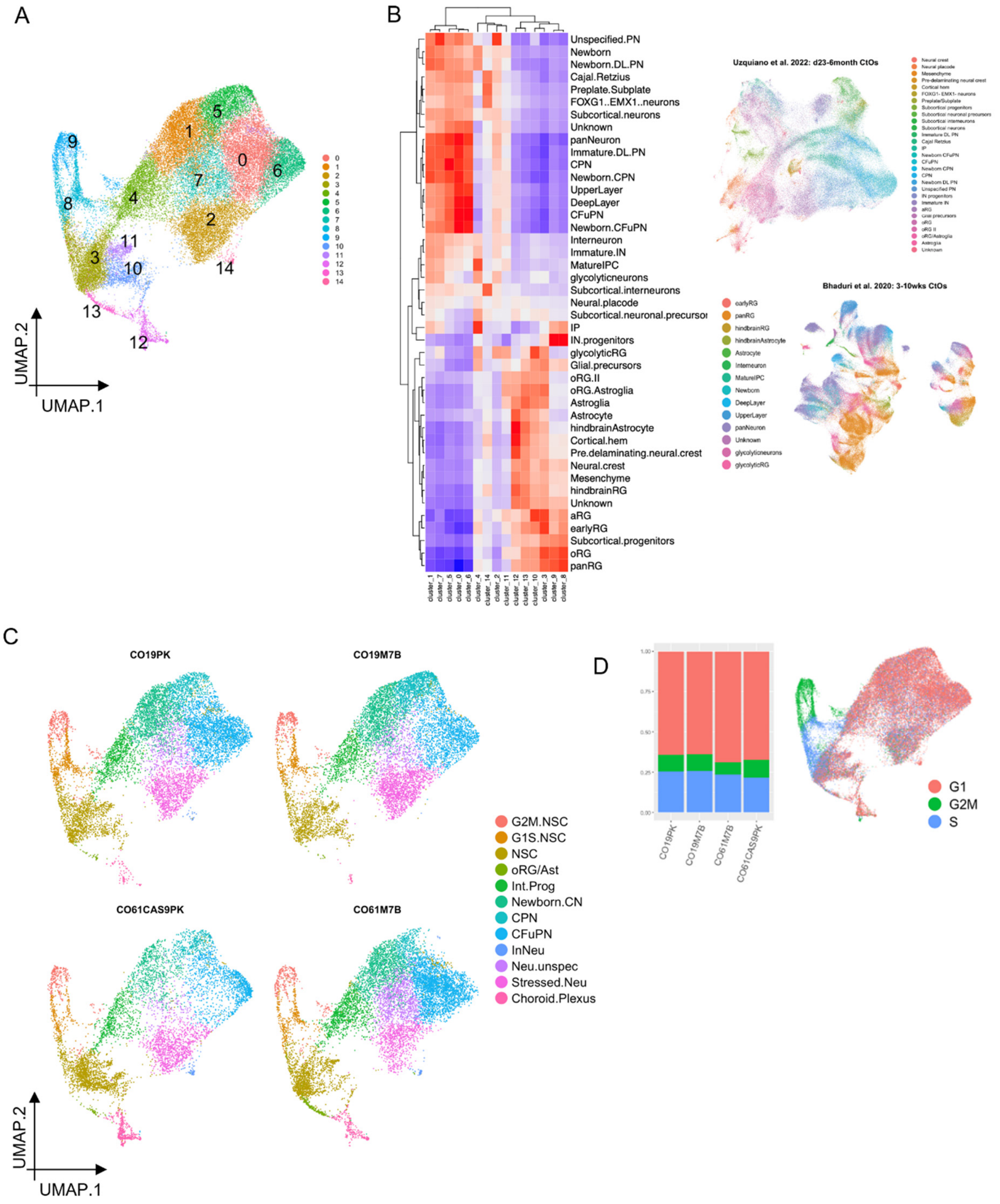

Figure S6 Single-cell RNAseq analysis and cell-type distribution in day70 CO derived from EPSC M7B^{OE}. Related to Figure 3

A. UMAP plot of CO samples coloured by Seurat clusters.
B. Heatmap showing Pearson correlation of cluster marker genes to reference cell type signatures.
C. UMAPs showing cell type annotations split by organoid sample.
D. UMAP and bar plot showing cell cycle state proportions assigned by module scoring.

Constantinou et al. Figure S7

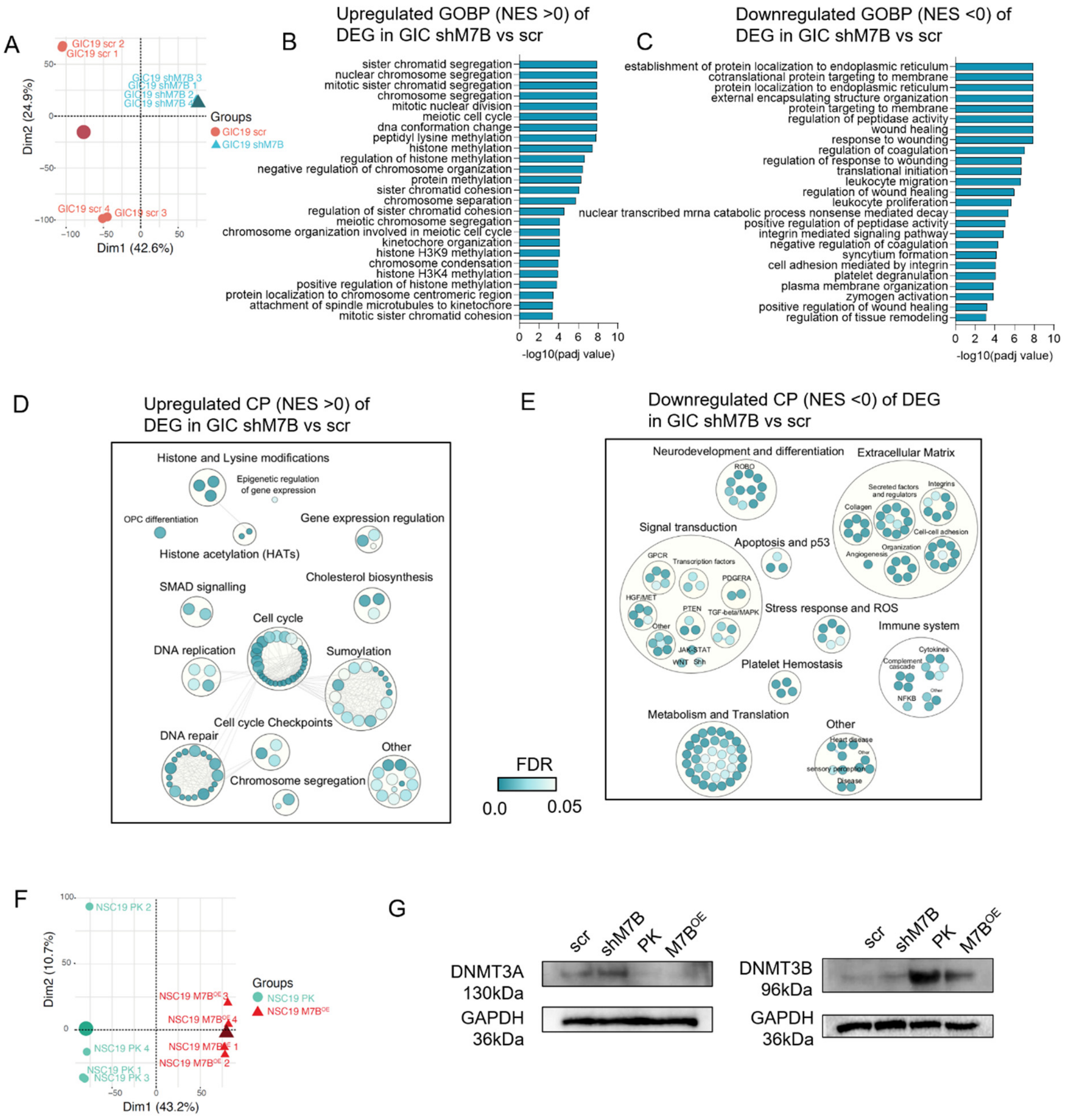

Figure S7 Bulk RNAseq analysis in GIC and NSC upon METTL7B modulation. Related to Figure 4

A. PCA plot of samples using gene expression of all detected genes, using gene expression log2 Reads per kilobase of transcript per Million reads (log2RPKM) of all sufficiently detected genes. Red circle: GIC19 scr replicas (n=4), dark red: centroid of GIC19 scr, Blue triangle: GIC19 shM7B replicas (n=4), dark blue: centroid of GIC shM7B.

B. Top 25 most significantly upregulated gene ontology biological processes (GOBP) (NES>0) of all DE genes identified in GIC19 shM7B as compared to scr. GSEA using differential expression statistic (t-statistic). Bars showing –log10(padj).

C. Top 25 most significantly downregulated gene ontology biological processes (GOBP) (NES<0) of all DE genes identified in GIC19 shM7B as compared to scr. GSEA using differential expression statistic (t-statistic). Bars showing –log10(padj).

D. Cytoscape visualisation of upregulated (NES>0) Canonical pathways (CP) of all DE genes identified in GIC19 shM7B as compared to scr. GSEA using differential expression statistic (t-statistic). Bubbles are coloured based on FDR values and size is proportional to the number of genes included in each CP term.

E. Cytoscape visualisation of downregulated (NES<0) Canonical pathways (CP) of all DE genes identified in GIC19 shM7B as compared to scr. GSEA using differential expression statistic (t-statistic). Bubbles are coloured based on FDR values and size is proportional to the number of genes included in each CP term.

F. PCA plot of samples using gene expression of all detected genes, using gene expression log2RPKM of all sufficiently detected genes. Green circle: iNSC19 PK replicas (n=4), dark green: centroid of iNSC19 PK, Red triangle: iNSC M7B^{OE} replicas (n=4), dark red: centroid of iNSC19 M7B^{OE}.

G. Protein expression levels of DNMT3A and DNMT3B upon silencing of METTL7B in GIC19, and overexpression of METTL7B in iNSC19. 20µg of protein lysate loaded for each sample. Representative images of n=3 replicas.

Constantinou et al. Figure S8

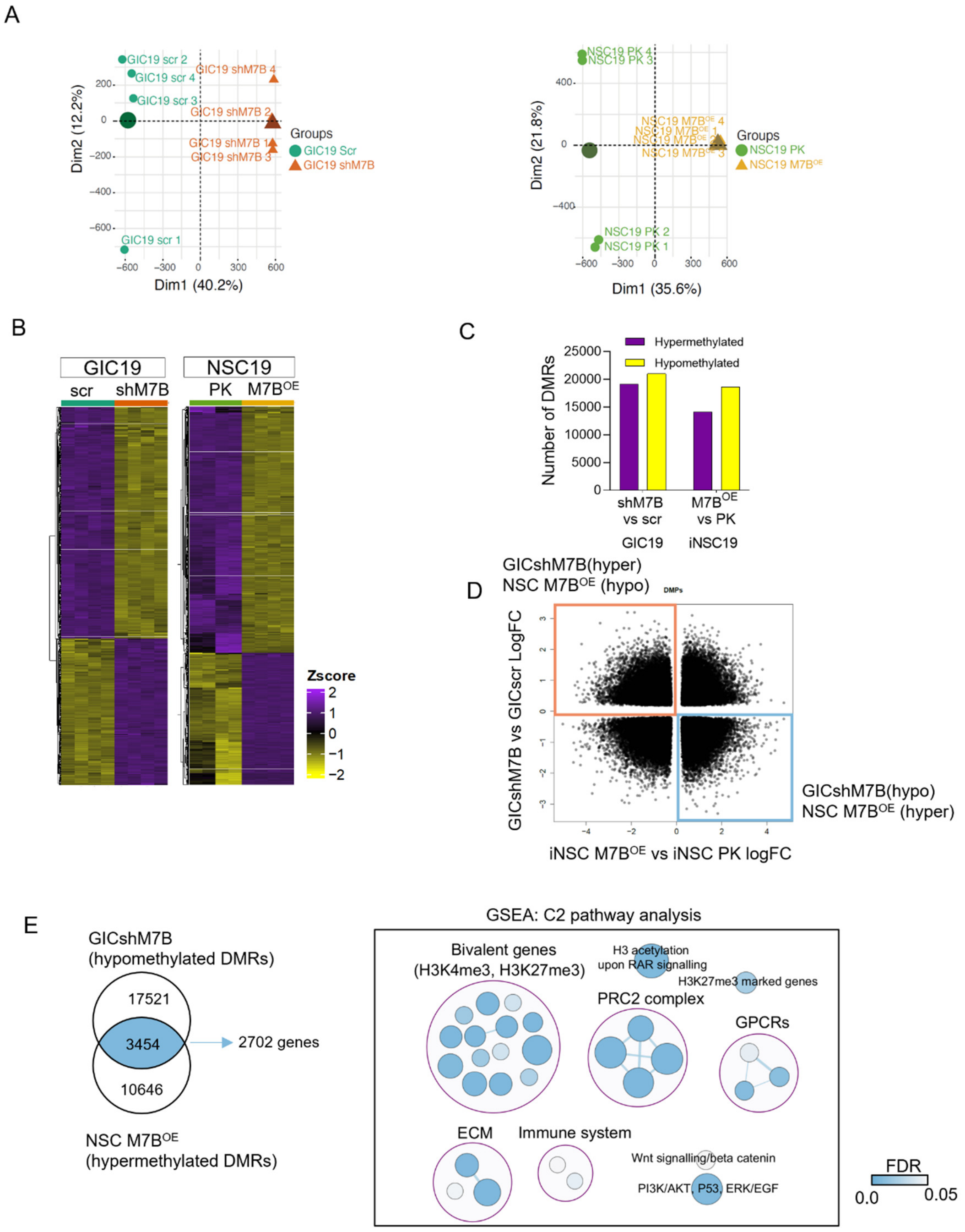

Figure S8 DNA methylation analysis in GIC and NSC upon METTL7B modulation. Related to Figure 4

A. PCA plots of samples based on M-values of all sufficiently detected probes. Top: Green circle: GIC19 scr replicas (n=4), dark green: centroid of GIC19 scr, Orange triangle: GIC19 shM7B replicas (n=4), dark orange: centroid of GIC19 shM7B, Bottom: Green circle: iNSC19 PK replicas (n=4), dark green: centroid of iNSC19 PK, Yellow triangle: iNSC19 M7B^{OE} replicas (n=4), dark yellow: centroid of iNSC19 M7B^{OE}

B. Heatmaps of beta-values of significantly affected Differentially Methylated Probes (DMPs) in the indicated contrasts. Left: GIC19 shM7B (green) vs GIC19 scr (dark orange), Right: iNSC19 M7B^{OE} (light green) vs iNSC19 control PK (light orange). DMPs with padj < 0.05 and logFC > |2| are illustrated on the heatmap. Colour range purple to yellow indicates high to low methylation.

C. Number of DMRs upon modulation of METTL7B in GIC19 (shM7B) and iNSC19 (M7B^{OE}) as compared to their respective controls (scr, PK).

D. Scatter plot of LogFCs of DMPs (padj<= 0.05). Intersect highlighted in orange: hypermethylated DMPs in GIC19 shM7B vs GIC19 scr and hypomethylated DMPs in iNSC19 M7B^{OE} vs iNSC19 PK. Intersect highlighted in blue: hypomethylated DMPs in GIC19 shM7B vs GIC19 scr and hypermethylated DMPs in iNSC19 M7B^{OE} vs iNSC19 PK

E. Venn diagram (left) of overlapping DMRs in the blue Intersect; hypomethylated DMRs in GICshM7B vs scr and hypermethylated DMRs in iNSC M7B^{OE} vs NSC PK. For hypermethylated regions: maxdiff > 0 & meandiff > 0 & Fisher <= 0.05; For hypomethylated regions: maxdiff < 0 & meandiff < 0 & Fisher <= 0.05. Right: Cytoscape visualization of GSEA: C2 pathway analysis of DMRs in the Intersect GIC hyper / iNSC hypo. Bubbles are coloured based on FDR values and size is proportional to the number of genes included in each C2 term.

Constantinou et al. Figure S9

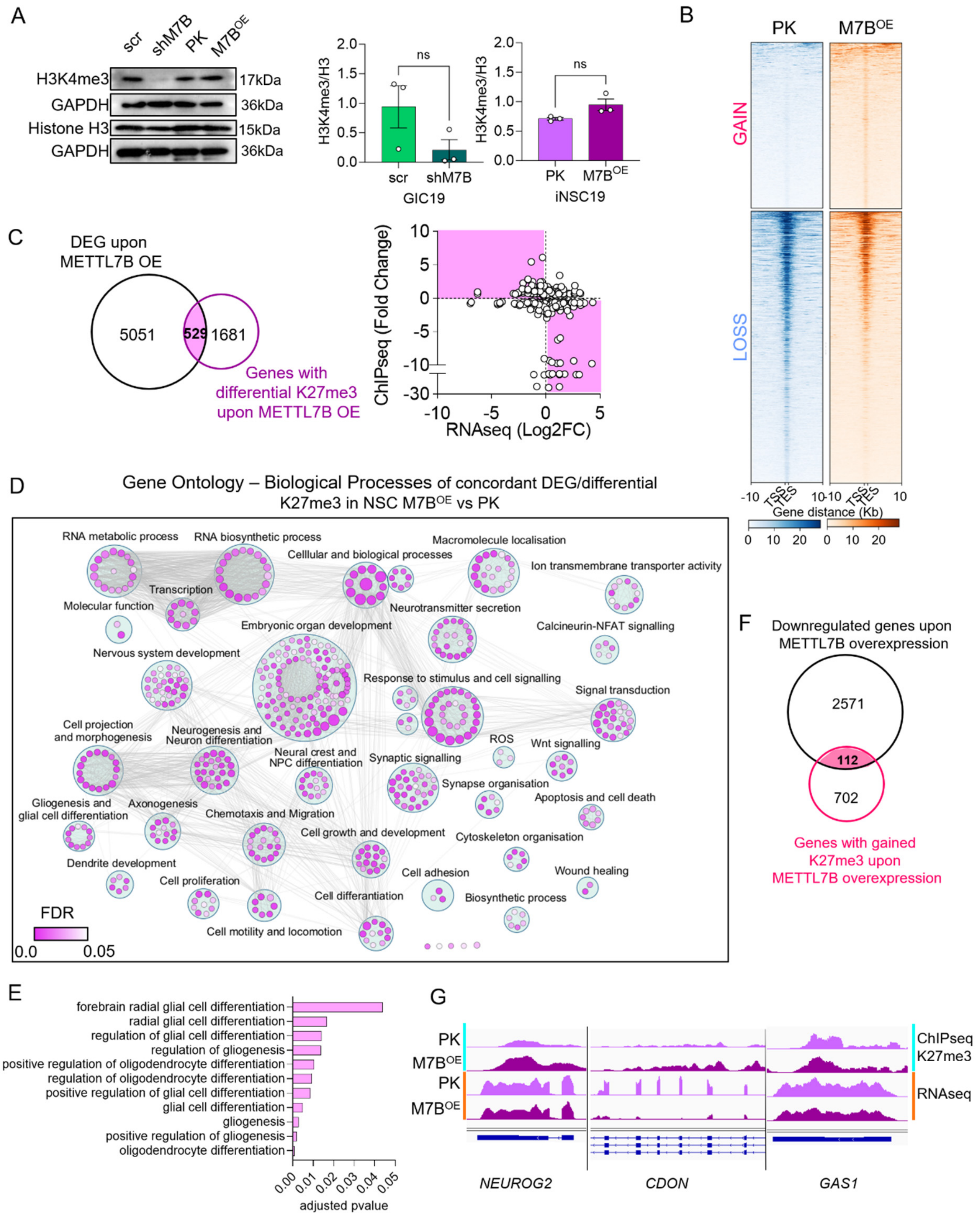

Figure S9 METTL7B epigenetically regulates genes involved in glial cell differentiation in iNSC M7B^{OE}. Related to Figure 5

A. Western blot (left) and quantification (right) of tri-methylation of H3K4/total H3 histone (H3K4me3) in scr (light green) and upon METTL7B silencing (dark green) in GIC19 or in control (PK, light purple) and in METTL7B overexpressing (M7B^{OE}, dark purple) iNSC19. GAPDH immunoreactivity was used to normalise protein loading. n=2 independent experiments, one-way ANOVA. All graphs report mean ± SEM of 3 blots. P values: *P < 0.05, **P < 0.01. Protein expression levels of H3K4me3 and Histone H3 upon silencing of METTL7B in GIC, and overexpression of METTL7B in iNSC
B. Heatmaps of H3K27me3 ChIP-Seq peak enrichments in control (PK, blue) and in METTL7B overexpressing (M7B^{OE}, orange) iNSC19. Heatmaps are centred at transcription start site (TSS) covering +/- 10Kb and ordered by H3K27me3 intensity. Signals were clustered based on the distribution of H3K27me3 surrounding the promoters and divided by genomic loci acquiring (GAIN) or loosing (LOSS) the H3K27me3 mark upon METTL7B overexpression.
C. Venn diagram showing the integration of DE gene from RNAseq and genes presenting differential H3K27me3 peaks from ChIPseq data (left). Volcano plot representing fold changes of expression for DE genes (RNAseq(Log2FC)) and fold change of H3K27me3 peak enrichment (ChIPseq (Fold Change)) upon METTL7B overexpression in iNSC19 (right). Highlighted genes with concordant gain/loss of H3K27me3 peaks and down/upregulation.
D. Bubble plot showing GO biological processes significantly enriched for the 333 concordant genes in iNSC19 upon METTL7B overexpression (highlighted in D, right panel) identified in ChIPseq and RNAseq integration. Bubble are coloured based on FDR values and size is proportional to the number of genes included in each GO term.
E. Histograms showing glial-related GO biological processes significantly enriched for the 333 concordant genes identified in iNSC19 upon METTL7B overexpression in ChIPseq and RNAseq integration.
F. Venn diagram showing the overlap between genes downregulated and with gain of H3K27me3 in iNSC19 upon METTL7B overexpression.
G. Genome browser view showing H3K27me3 ChIP-Seq and RNAseq peaks in NEUROG2, CDON, GAS1 genomic loci in GIC scr (light green) and upon METTL7B silencing (shM7B, dark green) or in iNSC control (PK, light purple) and upon METTL7B overespression (METTL7B^{OE}, dark purple).

Constantinou et al. Figure S10

A

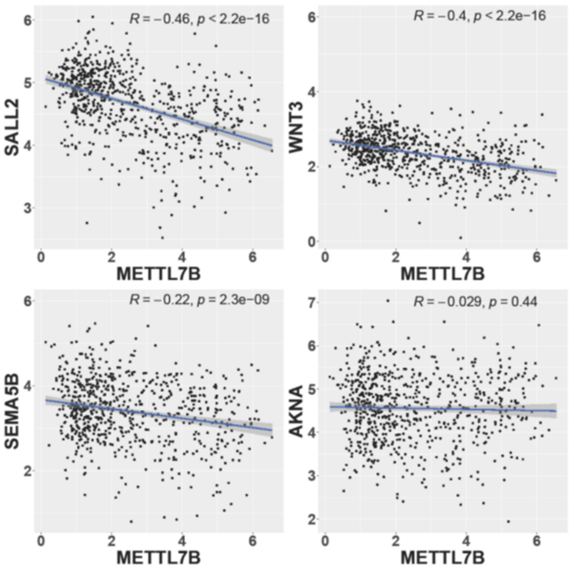

Figure S10 METTL7B expression negatively correlates with neuronal genes in TCGA glioma samples. Related to Figure 5 and 6

A. Correlation of METTL7B and SALL2, SEMA5B, WNT3 and AKNA in TCGA glioma samples.
